# Systematic Review of Non-Coding Genomic Variants in Globin and Non-Globin Clusters and Their Impact on Phenotypic Severity in Thalassemia and Sickle Cell Disease

**DOI:** 10.3390/jcm15041345

**Published:** 2026-02-09

**Authors:** Abeer M. Al-Subaie, J. Francis Borgio

**Affiliations:** 1 Department of Clinical Laboratory Sciences, College of Applied Medical Sciences, Imam Abdulrahman Bin Faisal University, Dammam 31411, Saudi Arabia; 2 Department of Genetic Research, Institute for Research and Medical Consultations (IRMC), Imam Abdulrahman Bin Faisal University, Dammam 31441, Saudi Arabia

**Keywords:** missing heritability, phenotypic severity, thalassemia, sickle cell disease, non-coding variants, foetal haemoglobin, globin gene cluster, emerging modifiers

## Abstract

**Background:** Haemoglobinopathies such as beta-thalassemia (β-thal), alpha-thalassemia (α-thal) and sickle cell disease (SCD) are characterised by pathogenic gene variations (mutations) in the globin genes. Patients with haemoglobinopathies have the same disease-causing coding variations with very different disease phenotypes, from requiring blood transfusions to being non-symptomatic. The gap between the expected clinical outcomes based on primary coding mutations (the genotype) and the actual observed symptoms (the phenotype) often remains unexplained. We refer to the contribution of secondary genetic modifiers—specifically, non-coding variants of the genome that alter globin gene expression and pathophysiology—as the “missing heritability” of the clinical presentation [Primary Mutation + Missing Heritability (Non-Coding Variants) = Actual Clinical Phenotype]. **Objectives:** This systematic review aims to find evidence connecting genetic differences outside of the protein-coding region, as in promoters, enhancers or untranslated regions (UTRs), to the clinical severity (phenotype) of beta-thalassemia, alpha-thalassaemia and SCD. We summarise the molecular basis of phenotypic variation among haemoglobinopathy patients with identical variations to reveal their missing heritability and to enhance our understanding of prognostic strategies. **Methods:** This systematic review was performed in accordance with the PRISMA 2020 guidelines. We used search terms related to haemoglobinopathies, non-coding variation, SNP, promoters, enhancers and clinical severity to search major databases (PubMed and Google Scholar) as of October 2025. A total of 527 (out of 572) abstracts were fit for initial screening to identify the eligible reports. Due to heterogeneity in study designs and reported outcomes, findings were synthesised descriptively and grouped by variant mechanism (*cis*-acting and *trans*-acting). The final analysis included 89 articles that demonstrated a direct association between a non-coding genomic variant and a quantitative measure of clinical severity. **Results:** Two main groups of non-coding variants (NCVs) that modulate foetal haemoglobin (HbF) induction were identified. The first major group comprises *cis*-acting variants within globin gene clusters (*HBG2* promoter *XmnI* polymorphism, *HBB* promoter mutations and α-globin enhancer variants), while the second major group comprises *trans*-acting quantitative trait loci (QTLs) (*BCL11A* and *HBS1L-MYB* loci). Non-globin NCVs in the UGT1A1 promoter were also found to influence the severity measures in β-thal and SCD. NCVs primarily alter the binding of transcription factors and the looping dynamics of chromatin, modulating the α/β chain balance ratio and γ-globin repression. The *XmnI* polymorphism is the most prominent cis-acting modifier associated with β-thal intermedia. The promoter polymorphisms in *TNF-α* and *VCAM1* are associated with vascular complications in SCD. **Conclusions:** NCVs are fundamental when determining the clinical measures of haemoglobinopathies, in addition to coding variants. NCV screening should be integrated for clinical prognosis for the accurate prediction of haemoglobinopathy severity and associated high-risk complications. NCVs may represent promising targets for next-generation gene editing and therapeutic intervention strategies aimed at modifying the severity of β-thal, α-thal and SCD.

## 1. Introduction

### 1.1. The Clinical Paradox of Haemoglobinopathies

Haemoglobinopathies are a group of Mendelian inherited disorders resulting from specific genetic defects in the globin genes [1]. Beta-thalassemia (β-thal) and sickle cell disease (SCD) are caused by mutations in the *HBB* gene, while alpha-thalassemia (α-thal) results from defects in the *HBA1* and *HBA2* genes. Even though haemoglobinopathies are monogenic, their clinical symptoms are remarkably diverse, with a wide spectrum of severity that ranges from asymptomatic to severe life-threatening conditions that require immediate medical support [2,3]. Individuals who are homozygous for the sickle cell mutation with mild SCD are characterised by sporadic vaso-occlusive crises (VOCs), low rates of organ damage and severe disease, leading to frequent hospitalisations, stroke and mortality in early SCD patients [4,5]. The difference between expected clinical outcomes based on primary coding mutations (the genotype) and actual observed symptoms (the phenotype) often remains unexplained in monogenic disorders. In haemoglobinopathies, this gap suggests that primary mutations alone do not dictate disease characteristics. We define the contribution of secondary genetic modifiers, particularly non-coding variants that modulate globin gene expression and pathophysiology—as the “missing heritability” of the clinical presentation, where this relationship is conceptualised as [Primary Mutation + Missing Heritability (Non-Coding Variants) = Actual Clinical Phenotype]. Identifying these modifiers is critical for refining prognostic accuracy and developing targeted therapeutic interventions. These unresolved associations have long necessitated the identification of additional genetic modifiers [6]. The most well-characterised genetic modifier pathways influencing the severity of SCD and β-thal are associated with the expression levels of foetal haemoglobin (HbF) and the co-inheritance of α-thal [7]. High levels of HbF significantly ameliorate both β-thal and SCD by substituting for deficient β-like chains and inhibiting HbS polymerisation [8]. Similarly, reduced α-globin chain synthesis, typically due to co-inherited α-thalassemia deletions, corrects the α/β chain imbalance characteristic of β-thal [2]. This systematic review aims to summarise and curate the evidence regarding inherited variations within the non-coding regions of the genome that modulate these modifier pathways and contribute to the clinical spectrum of severity.

### 1.2. Non-Coding Regions in Globin Switching

The expression of the β-globin gene cluster is regulated through a complex system known as globin switching. This is controlled by numerous *cis*-acting elements, including the β-globin locus control region (LCR), promoters, enhancers and silencers situated both upstream and downstream of the coding sequences [9]. NCVs are hypothesised to influence alpha and beta gene expression by altering the binding of erythroid transcription factors or by disrupting the three-dimensional looping architecture of the chromatin and impacting the efficiency of gene transcription. NCVs can also influence post-transcriptional control by affecting mRNA splicing, stability or translation within untranslated regions (UTRs) [10]. Understanding the specific NCVs on these regulatory elements is very important for dissecting the mechanisms underlying the quantitative differences in globin production that are associated with SCD and β-thal severity.

### 1.3. Objectives of the Systematic Review

This systematic review aims to achieve the following three primary goals. The first is to exhaustively catalogue the specific NCVs identified across HBB, HBA and non-globin QTLs that demonstrate a clear association with quantifiable clinical severity measures in β-thal, α-thal and SCD. The second is to categorise these NCVs based on their functional genomic location (promoter, enhancer, UTRs, intron/splice) and their primary associated pathway (HbF regulation and complication-specific modification). The last is to integrate functional and association data to identify a comprehensive model of phenotypic modification and high-priority non-coding targets for therapeutic gene manipulation.

## 2. Methods

### 2.1. Search Strategy and Data Sources

A systematic search was conducted across the PubMed and Google Scholar databases up to 2025, conducted in compliance with the PRISMA 2020 guidelines. The search strategy integrated medical subject headings (MeSH). The associated words refer to the three target diseases (β-thalassemia, α-thalassemia and sickle cell disease), alongside terminology associated with non-coding genetic elements (SNP, polymorphism, promoter, enhancer, intron, UTR, QTL) and clinical severity (HbF, phenotype, severity, transfusion).

### 2.2. Article Selection Criteria

Inclusion criteria

1. Original research articles (cohort studies, case–control studies and family studies).
2. Studies reporting a direct association between a non-coding genetic variant, defined as any variant/mutation outside of the coding or CDS (Coding DNA Sequence) region of the *HBA1, HBA2* and *HBB* genes or trans-acting factors and studies analysing quantitative measures of clinical severity (HbF level, haemoglobin level, transfusion requirement, VOC rate and organ damage).
3. Articles written in English.

Exclusion criteria

1. Review articles, meta-analyses, editorials and conference abstracts.
2. Studies concentrating exclusively on coding mutations (e.g., *β*^0^ or *β^+^* mutations).
3. Articles that only discuss drug mechanisms (e.g., hydroxyurea), pharmacological induction or in silico modelling without any patient data.
4. Studies mainly focused on the mechanism of gene editing (CRISPR/Cas9), unless this mechanism was utilised to confirm the specific non-coding target identified in association with clinical severity in humans.

### 2.3. Study Selection and Data Extraction

**Figure 1:**
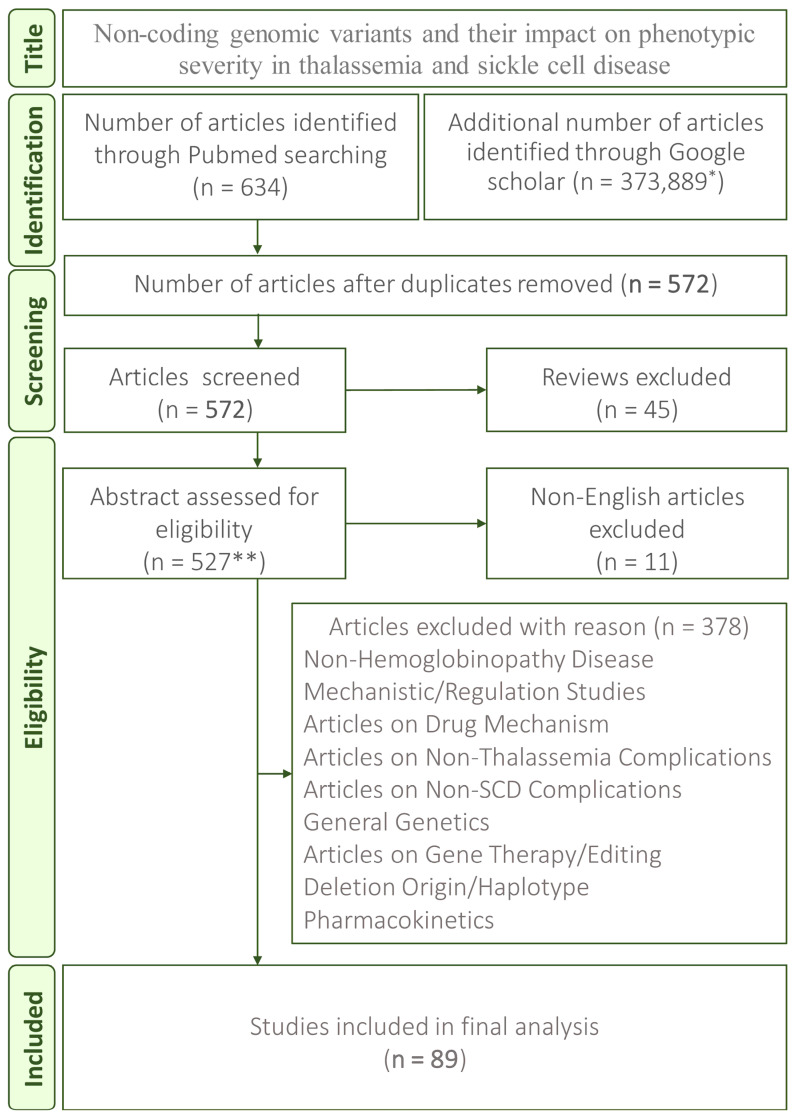
PRISMA flow diagram illustrating the selection process for articles reporting non-coding genomic variants and their impact on phenotypic severity in thalassemia and sickle cell disease. ** The full list of 527 screened articles is available in Supplementary Data S1. * For Google Scholar, the top 100 articles ranked by relevance were prioritised for screening. Additional methodological details are presented in Supplementary Figure S1. Checklist provided in Supplementary Data S2.

The initial search found 634 articles from PubMed and 373,889 from Google Scholar (the first 100 were reviewed). We ultimately identified 572 articles after excluding reviews and duplicates. After further screening the titles, more reviews were excluded (*n* = 45), and 527 were assessed for eligibility. The final screening of the titles and abstracts identified 89 articles for final analysis (Figure 1) [10,11,12,13,14,15,16,17,18,19,20,21,22,23,24,25,26,27,28,29,30,31,32,33,34,35,36,37,38,39,40,41,42,43,44,45,46,47,48,49,50,51,52,53,54,55,56,57,58,59,60,61,62,63,64,65,66,67,68,69,70,71,72,73,74,75,76,77,78,79,80,81,82,83,84,85,86,87,88,89,90,91,92,93,94,95,96,97,98]. From these articles, study designs, patient cohort characteristics, variant names, variant locations (promoter/enhancer/UTR/intron), associated phenotypes and disease impact severity data were extracted.

## 3. Non-Coding Variants and Mechanisms of Severity Modulation

### 3.1. Overview of Included Studies and Variant Classification

The systemic analysis of the 89 [10,11,12,13,14,15,16,17,18,19,20,21,22,23,24,25,26,27,28,29,30,31,32,33,34,35,36,37,38,39,40,41,42,43,44,45,46,47,48,49,50,51,52,53,54,55,56,57,58,59,60,61,62,63,64,65,66,67,68,69,70,71,72,73,74,75,76,77,78,79,80,81,82,83,84,85,86,87,88,89,90,91,92,93,94,95,96,97,98] selected research articles reveals that phenotypic severity in haemoglobinopathies is a polygenic trait, showing modulation of phenotypic severity caused by NCVs. The variants identified in the systematic analysis can be categorised into three functional classes. The first class of NCVs comprises *cis*-acting regulatory variants within the globin gene clusters (HBB, HBG and HBA). The second class comprises *trans*-acting quantitative trait loci, primarily regulating HbF. The third class comprises non-globin genes affecting related pathological pathways such as inflammation, coagulation and haemolysis. Promoter mutations and SNPs are the most frequent and functionally validated NCV types across the selected research studies.

### 3.2. Cis-Acting NCVs Within Globin Gene Clusters (HBB/HBG/HBD)

#### 3.2.1. γ-Globin Promoter Variants and Amelioration

**Figure 2:**
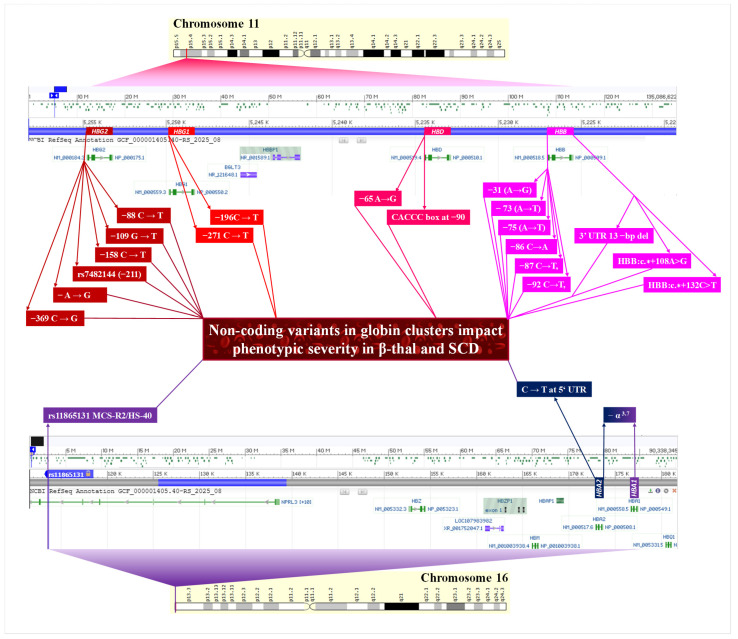
The most impactful non-coding variants in globin clusters significantly influence the phenotypic severity of β-thal and SCD.

NCVs in the foetal globin gene promoters (*HBG1* and *HBG2*) are well documented as the most direct influencing modifiers, resulting in the hereditary persistence of foetal haemoglobin (HPFH) and ameliorating the severity of SCD and β-thal [17,25,55,59,63,66,68,71,78,79,80,81,90,94,95,96,97]. The clinical impact of the beta-plus mutation can be significantly modulated by the NCV in XmnI. For instance, a case report of a single 63-year-old asymptomatic individual, homozygous for the −29 (A to G) β^+^ mutation, demonstrated an elevated HbF level of 83% in the presence of the XmnI polymorphism, underlining how specific non-coding interactions can lead to an extremely mild/asymptomatic β-thal phenotype [85]. The XmnI polymorphism (rs7482144)—a C → T substitution −158 base pairs upstream of the *HBG2* gene, is the most commonly validated modifier across both SCD and β-thal cohorts [63,68]. The presence of the homozygous T allele is strongly associated with significantly elevated HbF levels [63,68,97]. This finding is particularly notable in β-thal intermedia patients, where XmnI polymorphism seems to be the most determinant modulating factor, surpassing the effect of other genetic variants (Table 1). The link between a γ-globin gene promoter mutation (a non-coding variant) and reduced β-thal severity (transfusion independence) specifically underlines the XmnI polymorphism’s role as a modifier in patients with homozygous β^0^-thal [97]. Patients with homozygous β^0^-thal, a genotype usually indicating a severe major phenotype, have been observed with a mild or intermediate clinical type if they are homozygous for the XmnI polymorphism owing to high HbF levels. Homozygous non-deletion β^0^-thal and the co-inheritance of a γ-globin promoter mutation (−196 C → T) result in a symptomless clinical phenotype with very high (99.8%) HbF (Figure 2) [80]. The NCV *HBG2*:g. −109 G → T disrupts a binding site for key transcriptional repressors such as BCL11A or NF-E3. This loss of repression allows for the persistence of high γ-globin transcription into adulthood. Evidence suggests that the −109 G → T mutation may also concurrently activate both gamma globin *HBG1* and *HBG2* genes and be associated with high HbF, ameliorating the severity [59].

#### 3.2.2. HBB Promoter and UTR Variants

NCVs within the β-globin promoter (Table 1) are frequently characterised as mild β^+^-thal alleles, directly correlating with a less severe clinical presentation compared with severe splicing mutations (IVS-I-110) [20,44,81]. Mutations in critical consensus regions—such as the β-globin gene promoter mutation −73 (A → T) in CCAAT box [59,65] or the CACCC box (−87 C → T, −86 C → A) [30,88,92], reduce the binding affinity of essential erythroid transcription factors like EKLF, resulting in reduced but functional β-globin output. Patients who are compound heterozygous for these promoter NCVs and a severe β^0^ mutation often manifest with β-thal intermedia rather than major transfusion-dependent β-thal disease (Figure 2) [59,65]. A cohort analysis of HbS/β^+^ patients specifically shows that those whose β^+^ allele includes a promoter mutation experience a significantly lower median number of severe SCD-related events (VOC, ACS and stroke) than patients with the severe IVS-I-110 splicing mutation [20].

Variants in the UTRs of the *HBB* gene also function as NCVs. A 13 bp deletion in the 3′ UTR of the β-globin gene results in a β-thal major phenotype when compounded with a severe splicing mutation, confirming its role as a functional β^+^-thal allele that decreases the stability of mRNA [50]. Other 3′ UTR mutations (*HBB*:c.*+108A>G and *HBB*:c.∗+132C>T) are associated with milder-phenotype β-thal intermedia [44]. One study observed that NCV C33G SNPs in the 5′ UTR of HBB, associated with β-thal, may act as “RiboSNitches,” altering the structural ensemble of the mRNA transcript and affecting translation, leading to the β-thal phenotype [10].

#### 3.2.3. α-Globin Regulation and Chain Balance

Beyond their role as primary causes of α-thalassemia, variants within the α-globin cluster function as critical genetic modifiers of β-thal and SCD. These non-coding and structural variants modulate clinical severity primarily by regulating the ratio of α-chains to non-α-chains. The most common NCV is the α-thal gene deletion (−α^3.7^ deletion) [22,36,83,84]. Co-inheritance of the −α^3.7^ deletion in β-thal reduces the toxic excess of free α-globin chains, shifting the phenotype toward a milder presentation or intermedia status [83]. In SCD, the −α^3.7^ deletion is associated with reduced chronic haemolysis parameters, including lower total bilirubin and reticulocyte counts [36]. The regulatory control of α-globin is primarily mediated by a powerful enhancer: the multispecies conserved sequence (MCS) R2, also known as hypersensitive site-40 (HS-40) [22,73]. A gene-editing study directly established a functional link between deleting a non-coding element (enhancer) and correcting the pathology/severity of β-thal [24]. Functional validation using CRISPR/Cas9 genome editing to delete the MCS-R2 enhancer in primary human hematopoietic stem cells successfully reduced α-globin expression and corrected the pathological globin chain imbalance observed in β-thal cells, confirming the non-coding element as a direct therapeutic target for β-thal severity correction [24]. However, the influence of α-globin NCVs can be genetically complex. A regulatory SNP (rs11865131) located within the MCS-R2 enhancer was found to significantly interact with the −α^3.7^ deletion in SCD patients. While α-thal is generally protective against complications like stroke, the co-inheritance of this MCS-R2 NCV significantly negates this protective effect [22]. This shows the precise quantitative level of α-globin output determined by the interaction between the deletion NCV and specific enhancer NCVs.

### 3.3. Trans-Acting Non-Coding Quantitative Trait Loci (QTLs)

#### 3.3.1. NCVs in the BCL11A Locus

*BCL11A* encodes a crucial erythroid transcription factor that acts as a major repressor of γ-globin. It achieves this by directly binding to a conserved motif in the γ-globin promoters, which disrupts the chromatin looping required for the locus control region to activate γ-gene expression. This mechanism effectively shifts the transcriptional machinery toward the β-globin gene during the foetal-to-adult haemoglobin switch [99]. Polymorphisms within the *BCL11A* gene, predominantly intronic SNPs (rs4671393; rs11886868), constitute a highly significant quantitative trait locus, influencing adult HbF levels [19,40,55]. These intronic NCVs modulate the expression or function of *BCL11A,* and their HbF-boosting alleles are consistently associated with a milder clinical phenotype in β-thal and SCD [19,38].

An advanced analysis of SCD cohorts revealed that the ameliorating effect of NCVs on *BCL11A* extends beyond HbF mediation. Carriers of minor *BCL11A* alleles (rs9402686) showed a lower transfusion incidence rate, and this protective effect remains statistically significant even after adjusting for the direct effect of HbF concentrations [19]. These observations from various studies suggest that NCVs in the *BCL11A* influence physiological pathways in erythropoiesis, potentially affecting erythroid cell maturation or survival characteristics independent of their primary role.

#### 3.3.2. NCVs in the HBS1L-MYB Intergenic Region

The HBS1L-MYB intergenic region contains another major QTL controlling HbF levels [5,100]. NCVs within this 56 kb segment, including specific SNPs (rs9399137) and a 3 bp deletion (rs66650371), are associated with elevated HbF levels and ameliorate disease severity in β-thal and SCD [19,23,33,60]. The function of these intergenic variants is attributed to their location within conserved distal enhancer elements (HMIP-2) that regulate the expression of the *MYB* gene [28,33].

NCVs in the HMIP-2 region reduce the activity of the *MYB* core enhancer, leading to lower *MYB* expression levels. This decrease in MYB protein concentration promotes HbF induction through two distinct mechanisms. The first prolongs the erythroid maturation programme, which expands the pool of F-cells. The second reduces the expression of *BCL11A*, for which *MYB* is a required transcriptional activator. This synergistic effect effectively de-represses the γ-globin genes, ameliorating the clinical severity of haemoglobinopathies [19,28,34]. The critical nature of this regulatory element has been functionally validated, showing that the targeted in vivo deletion of the *MYB* distal enhancer (84 kb enhancer in humans/-81 kb in mice) acts on globin switching and corrects clinical erythroid parameters in a β-thal model [12].

Studies on the HBS1L-MYB locus have revealed the role of non-coding RNA pathways [23], regions that transcribe novel, long non-coding RNA (HMI-LNCRNA; chr6:135,096,362–135,097,644; hg38). Functional studies have demonstrated that downregulation of HMI-LNCRNA significantly upregulates *HBG* expression (by as much as 200-fold at the mRNA level), showing that this lncRNA is an important intermediary regulator linking intergenic NCVs to the foetal haemoglobin synthesis pathway [23]. This observation indicates that targeting ncRNAs in this region may offer a potent therapeutic approach for HbF induction in β-thal and SCD.

### 3.4. Non-Globin Non-Coding Variants

NCVs in genes outside the globin cluster contribute substantially to disease heterogeneity by modulating pathways related to chronic complications, independent of HbF levels. Table 2 summarises the most critical trans-acting NCVs in non-globin genes and their quantitative impact on specific complication endpoints in β-thal and SCD.

#### 3.4.1. Haemolysis and Hepatobiliary Complications (UGT1A1)

Chronic haemolysis in SCD and β-thal leads to high unconjugated bilirubin, predisposing patients to pigment gallstone formation (cholelithiasis). This secondary severity outcome is influenced by a non-coding short tandem repeat polymorphism (A(TA)nTAA) in the promoter of the *UGT1A1* gene, which encodes UDP Glucuronosyltransferase Family 1 Member A1, a critical enzyme for bilirubin conjugation [35,38,42,43,45,57,61,69,72,76]. The variant alleles, particularly (TA)7 and (TA)8 repeats, decrease *UGT1A1* expression in SCD and β-thal intermedia coexisting with Gilbert’s syndrome. Analyses of the A(TA)nTAA repeat polymorphism (a non-coding variant) in the *UGT1A1* promoter in patients with β-thal intermedia and SCD are directly linked to this non-coding [75]. Patients with homozygous (TA)7/(TA)7 or heterozygous (TA)7/(TA)8 genotypes in the *UGT1A1* gene exhibit significantly higher steady state bilirubin levels and an elevated risk of developing cholelithiasis, often at an earlier age [43,45,72]. This association demonstrates high confidence across diverse populations and in both SCD and β-thal patients [35,75]. The polymorphic *UGT1A1* gene acts as a pharmacogenetic biomarker for patients carrying high-risk genotypes, who often fail to achieve normalised bilirubin levels even under maximum tolerated doses of hydroxyurea, confirming the importance of NCVs in clinical management [42,57].

#### 3.4.2. Vascular, Inflammatory and Coagulation Complications

SCD severity related to acute events is modified by NCVs in vascular and inflammatory pathway genes. The TNF-α promoter polymorphism (*TNF*(−308)G/A) is strongly associated with large-vessel stroke risk in children with SCD. The GG genotype is linked with higher TNF-α expression and associated with a greater-than-threefold increased risk of large-vessel stroke (a major SCD complication) [64]. Similarly, non-coding haplotypes in the endothelial adhesion molecule *VCAM1* promoter increase promoter activity and are linked with cerebral vasculopathy and severe haemolysis in SCD [16,36].

Variability in the GT dinucleotide repeat in the *HMOX1* (heme oxygenase−1) gene promoter is associated with clinical outcomes where shorter alleles (≤25 repeats) lead to lower rates of hospitalisation for acute chest syndrome, suggesting a protective role via enhanced heme catabolism and antioxidant defence [48]. One study identified promoter and intronic SNPs in the coagulation factor *Protein Z* as potential modifiers with specific haplotypes (rs3024718A/rs3024719G/rs3024731T/rs3024735G and rs3024718G/rs3024719G/rs3024731T/rs3024735G), which are significantly associated with an increased frequency of VOCs (Table 2) [46].

#### 3.4.3. Emerging and Non-Canonical Non-Coding Modifiers

The investigation of NCVs has extended beyond established modifier pathways to reveal non-canonical effects. A novel intronic splice variant (c.307+1G>A in SUPT5H), identified in the non-globin transcriptional elongation factor gene *SUPT5H*, causes a β-thal trait phenotype (hypochromic microcytosis and increased HbA_2_) identical to that caused by a β-globin gene mutation, occurring in the absence of any defect in the β-globin gene itself [11]. This phenotypic mimicry occurs because the non-coding splice mutation leads to a loss of function in *SUPT5H*, a protein essential for normal globin transcription. Deficiency in this trans-acting machinery results in a mild globin gene defect, demonstrating that NCVs in regulatory genes can fully explain cases of haemoglobinopathy-like traits.

### 3.5. Resolving Phenotypic Heterogeneity via Non-Coding Regulation Network

**Figure 3:**
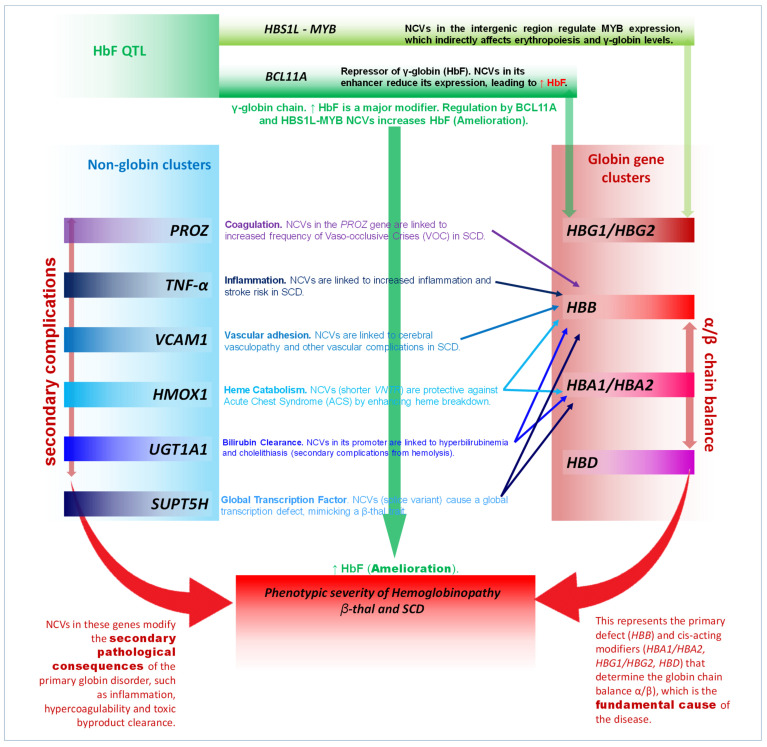
Conceptual network of non-coding variants modulating severity of haemoglobinopathy.

Our comprehensive analysis of 89 studies [10,11,12,13,14,15,16,17,18,19,20,21,22,23,24,25,26,27,28,29,30,31,32,33,34,35,36,37,38,39,40,41,42,43,44,45,46,47,48,49,50,51,52,53,54,55,56,57,58,59,60,61,62,63,64,65,66,67,68,69,70,71,72,73,74,75,76,77,78,79,80,81,82,83,84,85,86,87,88,89,90,91,92,93,94,95,96,97,98] from various countries reveals the influence of phenotypic variability in β-thal and SCD, as observed in the polygenic network of NCVs (Supplementary Table S1). This network impacts three hierarchical levels (Figure 3). The first level is the primary globin gene stoichiometry governed by α-globin deletion and *HBB* promoter/UTR NCVs. The second is the fundamental compensatory response from the induction of HbF controlled by *HBG cis*-elements and *trans*-QTLs BCL11A/HBS1L-MYB. Finally, the third comprises specialised complications controlled by non-globin NCVs in inflammatory/vasculature genes. This quantitative relationship suggests a hierarchy of modification. In individuals with extremely severe genotypes, such as homozygous β^0^ or HbSS, significant amelioration leading to a mild or silent phenotype requires the highest-impact NCVs, such as HPFH-level HbF induction (XmnI homozygosity) or the co-inheritance of α-thal. Conversely, *BCL11A* and HBS1L-MYB *trans*-QTLs often modulate the moderate spectrum of this disease by controlling the percentage of HbF, which influences β-thal and SCD severity within a predefined genotypic background (Figure 3). Evidence shows that SNPs in the X chromosome influence the number of F-cells and HbF levels, explaining previously described differences between males and females regarding HbF levels [101]. Most of this evidence was obtained from observational case–control and cohort studies, leading to a high risk of selection and confounding bias. Limitations include the observational nature of the included studies and significant heterogeneity in phenotype definitions, which cannot be excluded while considering observations, thus restricting the certainty of the evidence.

### 3.6. Molecular Convergence and Regulatory Epistasis

Most NCVs apply their effects by disrupting transcription factor binding and altering chromatin arrangements, serving as a central point of molecular convergence. NCVs in the globin promoter directly influence the affinity of erythroid-specific factors (*EKLF; GATA-1*) and quantitatively alter β-globin synthesis [65]. Similarly, NCVs from the γ-globin promoter inhibit the associations of repressor complexes that include BCL11A, resulting in persistent HbF [13]. The effectiveness of these NCVs is not completely understood, underlining the importance of more studies on the principle of regulatory epistasis. For example, some of the polymorphisms in *BCL11A* are generally potent HbF boosters, but they play a diminished role in determining the phenotype of β-thal patients when they carry an unfavourable LCR, such as homozygosity for the “A” allele at the 5′HS4-LCR palindromic polymorphic site [40]. This indicates that we need a deeper understanding of the molecular mechanisms and population-specific effects by which NCVs functionally prioritise transcription regulation across the full range of phenotypic severity.

A study of the HBS1L-MYB locus revealed another layer of complexity involving non-coding RNA. The genomic association of SNPs in the distal enhancer is not solely mediated by protein–DNA interactions that regulate *MYB* mRNA. This association also generates a transcribed element, HMI-LNCRNA, while functional depletion significantly upregulates *HBG* expression [23]. This regulatory cascade involving NCV modulates the expression or stability of non-coding RNA, which acts as a trans-regulator of the γ-globin genes and provides a deeper understanding of this mechanism. This explains the aforementioned genetic association and offers a new class of pharmacological targets.

### 3.7. Translational and Clinical Relevance of Non-Coding Variants

These NCVs provide immediate and long-term translational benefits for haemoglobinopathy management.

#### 3.7.1. Personalised Prognostic Genotyping

Accurate prediction of clinical severity requires moving beyond primary coding mutations to a comprehensive NCV panel. Refined risk stratification can be achieved by genotyping NCVs such as the XmnI polymorphism, *BCL11A* and HBS1L-MYB SNPs; the α-thal deletion; and non-globin NCVs like *TNF*-α and *VCAM1* promoter haplotypes. For example, identifying the high-risk *TNF*-α GG genotype provides important information for stroke surveillance and preventative management in young SCD patients [64]. Similarly, simultaneous genotyping of the α-thal deletion and the *MCS-R2* enhancer SNP (rs11865131) is necessary for accurate stroke risk prediction, as the enhancer NCV can negate the protective benefits of the α-thal deletion [22].

#### 3.7.2. Pharmacogenetics and Monitoring

The UGT1A1 promoter VNTR exemplifies a crucial pharmacogenetic application. The (TA)7/(TA)7 genotype can identify a subset of patients who will experience chronic hyperbilirubinemia refractory to the bilirubin-lowering effects of hydroxyurea [76]. This genotypic knowledge is vital for proactive monitoring and intervention in these patients to prevent gallstone-related complications.

#### 3.7.3. Non-Coding Targets for Gene Editing

The functional validation of specific NCVs has led to the development of highly precise therapeutic strategies beyond the α-globin cluster. The feasibility of correcting β-thal pathology by targeting this cluster is demonstrated by the successful use of CRISPR/Cas9 editing to delete the MCS-R2 enhancer [24]. This approach effectively reduces α-globin production, correcting the pathological imbalance without needing to correct the primary β-globin mutation. Furthermore, the molecular elucidation of the erythroid-specific enhancer of *BCL11A* (specifically, the +58 kb DNase I hypersensitive site) has led to the first regulatory-approved gene-editing therapies. By disrupting the GATA1-binding motif within this non-coding element, *BCL11A* expression is selectively silenced in erythroid cells, resulting in reactivation of HbF [13,23]. Recent studies have focused on directly targeting γ-globin promoters to mimic naturally occurring HPFH mutations, using CRISPR/Cas9 to disrupt the binding sites of repressors such as BCL11A or LRF (ZBTB7A). This strategy permanently activates HbF expression by preventing the developmental silencing of the foetal globin genes [99].

## 4. Conclusions

This systematic review demonstrated that non-coding genomic variants are among the principal architects of phenotypic heterogeneity in β-thal, α-thal and SCD. The haemoglobinopathies β-thal, α-thal and SCD are fundamentally primary monogenic disorders, but their variable clinical severity and manifestations are determined by polygenic modifications, including NCVs. The successful delineation and functional validation of NCVs—ranging from the γ-globin promoters and *BCL11A*/HBS1L-MYB$ QTLs to complication-specific non-coding variants in genes like *UGT1A1* and TNF-α, marks an advanced phase in haemoglobinopathy genetics. The evidence from this review necessitates a shift in clinical practice toward integrated polygenic risk stratification. By adding genotype classifications of comprehensive NCV profiles, clinicians can achieve significantly enhanced prognostic accuracy and offer more precise genetic counselling. The functional characterisation of non-coding elements would provide a robust foundation for next-generation therapeutic developments in gene editing and the highly specific manipulation of endogenous gene regulation pathways.

## Figures and Tables

**Table 1:** Cis-acting non-coding variants in globin loci and association with clinical severity.

Haemoglobinopathy	Variant Location (Gene)	NCV Type	Molecular Mechanism	Severity Measure/Outcome	Observed Phenotypic Effect	References
SCD and β-thal	*HBG2* promoter (−158 C → T XmnI)	SNP/promoter	Putative alteration of promoter accessibility (exact mechanism under investigation)	HbF level, transfusion needed	Ameliorated severity (β-thal intermedia phenotype) and SCD; high HbF levels	[63,68,97]
β-Thal	*HBB* promoter [−73 (A → T) in CCAAT box, CACCC box (−87 C → T, −86 C → A]	SNP/promoter	Reduced EKLF/GATA-1 binding; decreased β-chain transcription	Anaemia severity, β^+^-thal classification	Mild/intermedia phenotype; lower severity than splicing mutants	[20,30,59,65,88,92]
α-Thal/β-Thal	*HBA* gene cluster (−α^3.7^ deletion)	Deletion	Gene dosage reduction (α-chain)	Globin chain balance, haemolysis/bilirubin	Ameliorating effect on β-thal; reduced bilirubin/haemolysis in SCD	[22,36,83,84]
α-Thal	α-Globin enhancer (MCS-R2/HS-40)	Deletion/SNP	Loss of enhancer function	α-Globin expression, stroke risk (SCD)	Severe downregulation of α-globin; enhancer SNP (rs11865131) negates protective effect against stroke	[22]
δ-Thal/SCD	*HBD* promoter (CACCC box at −90 in δ-globin)	Defective element	Absent EKLF binding	HbA_2_ level, sickling inhibition	Contributes to low HbA_2_ in SCD, potentially exacerbating severity	[88]
β-Thal	*HBB* 3′ UTR deletion (+90 del 13 bp)	Deletion/UTR	Reduced β-globin mRNA stability	β-Thal major/intermedia	Functionally mild β^+^-thal allele, leading to major phenotype owing to compound heterozygosity with IVSII-1 (G → A)	[50]

**Table 2:** Trans-acting non-globin non-coding variants modifying clinical outcomes in SCD and β-thal.

Haemoglobinopathy	Modifier Gene/Locus	NCV Type/Location	Molecular Pathway	Clinical Outcome Measure	Observed Association	References
SCD and β-thal	*UGT1A1*	VNTR (A(TA)nTAA)/promoter	Bilirubin glucuronidation	Hyperbilirubinemia, cholelithiasis risk	(TA)7/(TA)7 genotype significantly increases complication risk and reduces hydroxyurea efficacy in bilirubin	[43,45,72]
SCD	*TNF-* *α*	SNP (−308 G/A)/promoter	Inflammation regulation	Large-vessel stroke risk	GG genotype associated with >3-fold increased risk of stroke	[64]
SCD	*VCAM1*	Promoter haplotypes/SNPs	Endothelial adhesion/inflammation	Cerebral vasculopathy, chronic haemolysis	Risk haplotypes increase promoter activity, linked to stroke and severe haemolysis	[16,36]
SCD	*HMOX1*	VNTR (GT repeat)/Promoter	Heme catabolism, antioxidant response	Acute chest syndrome (ACS) incidence	Shorter alleles associated with lower rates of hospitalisation for ACS	[48]
SCD	*Protein Z (PZ)*	Promoter/intron SNPs	Coagulation, thrombotic state	VOC	Specific SNPs/haplotypes linked to increased frequency of VOC	[46]

## Data Availability

The original contributions presented in the study are included in the article/Supplementary Materials.

## Supplementary Materials

The following supporting information can be downloaded at https://www.mdpi.com/article/10.3390/jcm15041345/s1, Supplementary Data S1: List of articles screened in the study. Supplementary Data S2: PRISMA 2020 Checklist and PRISMA 2020 for Abstracts Checklist. Supplementary Table S1: Summary of non-coding variant types and their correlation with phenotypic severity of sickle cell disease (SCD) and thalassemia. Supplementary Figure S1: PRISMA 2020 flow diagram for studies on non-coding genomic variants in thalassemia and SCD.
