# Supplementary material for: Systematic Review of Non-Coding Genomic Variants in Globin and Non-Globin Clusters and Their Impact on Phenotypic Severity in Thalassemia and Sickle Cell Disease
Source: J Clin Med. 2026 Feb 9;15(4):1345. doi: 10.3390/jcm15041345 (PMC12941110; doi:10.3390/jcm15041345)
Supplement: Supplementary file 1 [file jcm-15-01345-s001.zip › jcm-4040495-supplementary.pdf]

Supplementary Table 1: Summary of non-coding variant types and their correlation with phenotypic severity of Sickle Cell Disease (SCD) and Thalassemia.

PMID	Article Summary	Non-coding Variant Type	Phenotypic Severity Measure/Link	Observations
1379474	Haplotypes in SS patients... associated with elevated HB F.	Locus Control Region (LCR) HS−2 sequences and Gγ promoter within a hybrid haplotype.	Elevated HbF in SCD.	Links a non-coding LCR/promoter element to the key modifier HbF level in SCD.
1380206	β-thalassemia intermedia: relationship to mutations in the β-gene promoter.	Promoter mutations (−88 C→T, −87 C→A, −29 A→G).	High HbA2 beta-thalassemia intermedia phenotype	β-thalassemia intermedia (anemia severity) and high HbA2 levels.
1419783	Australian β0-thalassaemia... due to a 12 kb deletion commencing 5′ to the β-globin gene.	Large 5′ deletion removing sequences 5′ to the promoter.	Unusually high HbA2 phenotype.	Links a non-coding deletion/flanking region loss to a measure of severity.
1515647	δ-thalassemia caused by disruption of the GATA−1 site in the δ-globin gene promoter.	Promoter mutation (−77) abolishing GATA−1 binding.	Close association with the thalassemia phenotype	δ-thalassemia phenotype (impaired gene expression/synthesis).
1550780	Promoter mutations producing mild β-thalassaemia.	Promoter mutations (−87 C→T, −86 C→A).	Mild β-thalassaemia (thalassemia intermedia to late-presenting major).	Links promoter variants to the spectrum of phenotypic severity.
1577473	βs haplotypes in various world populations..	Promoter mutations (Mutations in the promoter sequences of the Gγ- and Aγ-globin genes).	Milder expression of the disease (inferred from the study's	Non-coding promoter mutations) associated with a milder expression

			objective) and Hematological data (average values) for SS patients grouped by βs haplotype.	of the disease. By providing Hb data by haplotype, it directly correlates the presence of the haplotypic non-coding variants with a measure of severity/disease outcome.
1698102	Observations on the levels of Hb A2 in patients with different β-thalassemia mutations and a delta chain variant... The −88 C→T promoter mutation is linked to increased Hb A2 and Hb B2 levels.	Promoter mutation (−88 C→T β-globin gene promoter mutation).	Increased Hb A2 and Hb B2 levels (by 200% and 80% respectively) in the presence of a delta chain variant (Hb B2).	Direct link between a promoter mutation (non-coding variant) and a significant quantitative change in the levels of Hb A2 and its variant.
1709779	Gγ and Aγ globin genes are identical from −471 of the promoter midway through γIVSII... Three new mutations were found in γ IVSII.	Intronic mutations found in γ IVSII.	Elevated Fetal Hemoglobin (Hb F) (≥12%) in Sickle Cell Disease (SS) patients.	Direct link between multiple intronic mutations (non-coding variants) in the γ-globin gene and the elevated Hb F levels, which is a measure of reduced phenotypic severity in SCD.
1725229	Molecular basis of β-thalassemia intermedia in a southern Italian region... The C→T substitution at position −158 5′ of the Gγ-globin gene is linked to attenuated forms.	Promoter mutation (C→T at −158 5′ of Gγ gene).	Attenuated β-thalassemia (transfusion-independent thalassemia inte rmedia or late-presenting thalassemia maj or).	Direct link between a γ-globin gene promoter mutation (a non-coding variant) and reduced disease severity (transfusion independence), specifically noting its role as a modifier in patients with homozygous β0-thalassemia.
8438884	Mild type of Hb S−β+-thalassemia... −92 C→T of the β promoter.	β promoter mutation (−92 C→T).	Mild type of Hb S−β+-thalassemia (clinical severity).	Direct link between a promoter mutation (−92 C→T) and milder disease severity.
8571932	Two mutations in the LCR HS-2 of haplotype 19 βs chromosomes alter binding of trans-acting	Locus Control Region (LCR) SNP (A→G at 8598 in 5’ HS-2); Locus Control Region (LCR) SNP (A→T at 9114 in	SNP (A→G at 8598 in 5’ HS-2) Increased binding of Sp1 which is linked	SNP (A→G at 8598 in 5’ HS-2) has link between a non-coding LCR variant (enhancer element)

	factors	5’ HS-2)	to the Benin haplotype's low HbF level (1-10%); low HbF is a measure of increased SCD severity. SNP (A→T at 9114 in 5’ HS-2) sharply decreased binding of an unknown trans-acting factor which is linked to the Benin haplotype's low HbF level (1-10%); low HbF is a measure of increased SCD severity.	and altered transcription factor binding, proposing a molecular mechanism for the observed differences in HbF (a key severity measure) among SCD patients with the same βS mutation.; SNP (A→T at 9114 in 5’ HS-2) has link between a non-coding LCR variant and altered transcription factor binding, proposing a molecular mechanism for the observed differences in HbF (a key severity measure) among SCD patients with the same βS mutation.
8916973	Activation of δ-globin gene expression... for gene therapy of Sickle Cell Disease	Defective Promoter Element (CACCC box at -90 in δ-globin promoter)	Low HbA2 levels (a measure of δ-globin expression) which fails to inhibit HbS polymerization, thereby exacerbating Sickle Cell Disease severity.	Directly links a defective non-coding promoter element to low expression of a globin chain (δ-globin), which acts as a known anti-sickling agent. This demonstrates how a non-coding variant modulates the molecular basis of SCD severity.
8943885	An IVS1-116 (A→G) acceptor splice site mutation in the α2 globin gene causing α+ thalassaemia	Intronic Splice Site Mutation (IVS1-116 A→G, Acceptor site)	Causes the α+ thalassaemia phenotype in carriers, resulting from the absence of functional mRNA (a measure of gene function/anemia severity).	Direct link between a non-coding intronic splice site mutation and the resulting thalassemia phenotype. This falls under the objective of characterizing studies linking non-coding variations to the clinical severity

				of α-Thalassemia.
9056561	Molecular basis of asymptomatic β-thalassemia major (describes modification of severe β-thalassemia)	HBB Promoter Mutation (-29); Polymorphism (Xmn I SNP) upstream of the Gγ-globin gene	HBB Promoter Mutation (-29) contributes to the β-thalassemia phenotype but, in combination, results in an asymptomatic/extremely mild β-thalassemia phenotype (reduction in severity/avoidance of transfusion dependence). Xmn I SNP associated with elevated Hemoglobin F (HbF) level of 83%; HbF elevation confers an extremely mild/asymptomatic phenotype.	Direct link between a promoter mutation (-29) (non-coding) and the overall clinical severity of β-thalassemia major. Link between a non-coding upstream Xmn I SNP (modifier) and the primary mechanism of amelioration (high HbF), explaining the missing heritability in a severe genotype.
9140720	A significant β-thalassemia heterogeneity in the United Arab Emirates	α-Globin Gene Deletion (-α 3.7 kb deletion)	Ameliorating effect on the phenotype of β-thalassemia (reduction in severity/improved clinical outcome).	This study directly links a modifier variant (α-thalassemia deletion), which is a non-β-globin coding change, to the phenotypic severity of β-thalassemia. This demonstrates the modification of a primary mutation's severity by a secondary genomic variant.
9143928	Scanning method... of δ-globin gene (detection of novel promoter substitutions)	Promoter mutation (e.g., -65 A→G)	Responsible for δ-thalassemia, linked to altered δ-globin gene expression and slightly increased HbA2 levels (a key hematological	Direct finding of a novel non-coding promoter mutation that alters globin gene expression and is responsible for a thalassemia phenotype.

			measure in thalassemias).	
9158662	Relative levels of α-, β-, and γ-mRNA... β-thalassemia major (links α/β-mRNA ratio differences to variable severity)	Hypothesized Promoter, Enhancer, and/or Locus Control Region (LCR) mutations	Explains variability in phenotypic severity (Thalassemia Intermedia vs. Transfusion-Dependent Severe Conditions) for patients with otherwise identical primary β-thalassemia mutations.	Mutations in promoter, enhancer, and/or locus control region sequences are likely responsible for the differences in disease severity observed for patients with the same primary β-globin gene mutation.
9234571	Silent thalassemias: genotypes and phenotypes (analysis of mild α- and β-thalassemia)	Promoter mutation (-101 C→T). Intronic mutation (IVS II 844 C→G)	Promoter mutation (-101 C→T) linked normal hematological picture with slightly raised Hb A2; classified as silent β+ thalassemia. Intronic mutation (IVS II 844 C→G) linked phenotype is even closer to normal; classified as silent β+ thalassemia.	Direct link between a promoter mutation (non-coding -101 C→T) and a measure of mild/silent phenotype. Direct link between an intronic mutation (non-coding IVS II 844 C→G) and a measure of very mild/silent phenotype.
12093744	An embryonic/fetal β-type globin gene repressor contains a nuclear receptor TR2/TR4 heterodimer.	Promoter Mutation (DR1 site mutation in γ-globin promoter)	Elevated γ-globin transcription (HPFH phenotype/high HbF) which ameliorates SCD.	References a natural non-coding DR1 site mutation in the γ-globin promoter that causes HPFH (high HbF), a powerful modifier of disease severity.
12176917	Homozygosity for nondeletion delta-β0 thalassemia resulting in a silent clinical phenotype. (Case of severe β-thalassemia	Aγ-globin Promoter mutation (-196C>T)	Silent clinical phenotype; High Hb F output (99.8%Hb F)	Direct link between a γ-globin promoter SNP and a severe β-thalassemia genotype resulting in a

	genotype resulting in a symptomless phenotype due to very high HbF)			symptomless/mild clinical phenotype due to very high HbF levels.
12210807	Spectrum of β-thalassemia mutations and their association with allelic sequence polymorphisms at the β-globin gene cluster in an Eastern Indian population. (Study investigating polymorphisms in cis-acting regulatory regions to understand genotype-phenotype correlations)	β-globin Locus Control Region (LCR HSs), γ-globin Promoter region, 3′ flanking region (Allelic Sequence Polymorphisms/Haplotypes)	Varying disease severity in β-thalassemia (genotype-phenotype relationship)	Explicitly studies the association of polymorphisms in cis-acting regulatory regions (LCR HSs, promoter regions, 3′ flanking) with the genotype-phenotype relationship and varying disease severity in β-thalassemia.
12393616	Variants in the VCAM1 gene and risk for symptomatic stroke in sickle cell disease. (Study analyzing non-coding SNPs in VCAM1 as genetic risk factors for a major clinical outcome in SCD)	Promoter SNPs and intronic regions (VCAM1 gene)	Risk for symptomatic stroke (a measure of phenotypic severity in SCD)	Investigated promoter SNPs and intronic regions of the VCAM1 gene for association with a major phenotypic severity measure in SCD: symptomatic stroke.
12614224	De novo deletion within the telomeric region flanking the human α globin locus as a cause of α thalassaemia. (Identification of a large deletion removing key regulatory elements, causing α-thalassemia)	Regulatory Element Deletion (HS-40 and HS-33/Enhancers)	Severely downregulated α-globin expression (causing α-thalassemia)	Direct link between a major non-coding deletion removing regulatory elements (enhancers HS-40, HS-33) and the resulting severe downregulation of α-globin expression, causing α-thalassemia.
12677174	UGT1A promoter polymorphisms influence bilirubin response to hydroxyurea therapy in sickle cell anemia. (UGT1A promoter variants affect bilirubin levels, a marker of hemolysis/severity, in SCA)	UGT1A Promoter polymorphism (e.g., 6/7 or 7/7 genotypes)	Serum Bilirubin levels (and influence on cholelithiasis/gallstone formation)	Direct link between a promoter polymorphism (UGT1A promoter) and a measure of clinical severity in SCA: serum bilirubin levels and the risk of gallstone formation.
12779270	Analysis of β-thalassemia mutations	β-globin Promoter mutations (-31 A>G and	β-thal major phenotype (a	Reports β-globin promoter mutations

	in northern Thailand using an automated fluorescence DNA sequencing technique. (Reports the identification of novel β-globin promoter mutations)	-87 C>A)	measure of anemia severity/transfusion dependence)	(-31 A>G and -87 C>A) that, when compound heterozygous with a β0-thal mutation, result in the β-thal major phenotype (a measure of severity).
12850481	Analysis of the A(TA)nTAA configuration in the promoter region of the UGT1A1 gene in Greek patients with thalassemia intermedia and sickle cell disease. (Analysis of a promoter polymorphism known to affect bilirubin in both patient groups)	UGT1A1 Promoter polymorphism (A(TA)n TAA repeat)	Thalassemia intermedia and Sickle Cell Disease (analyzing a modifier variant in these patient groups)	Analyzes the A(TA)nTAA repeat polymorphism (a non-coding variant) in the UGT1A1 promoter in patients with β-thalassemia intermedia and SCD, directly linking a non-coding variant to a known severity modifier locus.
16829478	[β0/β0 thalassemia with a mild phenotype]. (A severe β-thalassemia major patient has a mild course due to an XmnI promoter polymorphism)	Gγ gene Promoter polymorphism (XmnI -158, C>T)	Mild/intermediate phenotype (alleviated severity); High level of synthesis of foetal hemoglobin (HbF)	Direct link between the homozygous XmnI -158 C>T promoter polymorphism and the amelioration of a severe β-thalassemia major phenotype to a milder form, due to high HbF levels.
17178667	Molecular pathogenesis and clinical variability of homozygous β0-thalassemia in populations of Jammu region of $\text{J}&\text{K}$ state (India).	γ-globin gene non-coding SNP (XmnI polymorphism)	Lesser severity of β0-thalassemia (transfusion dependence vs. non-dependence/intermedia phenotype)	Direct link between a known non-coding SNP modifier (XmnI) and clinical severity/transfusion dependency in β-thalassemia
17516066	A novel mutation of −73(A→T) in the CCAAT box of the β-globin gene identified in a patient with the mild β-thalassemia intermedia.	β-globin gene promoter mutation (novel −73(A→T) in CCAAT box)	Mild β-thalassemia intermedia phenotype, transfusion independent	Direct link between a novel β-globin promoter mutation (non-coding) and a reduced-severity clinical phenotype
17593033	The linear effects of α-thalassaemia, the UGT1A1 and HMOX1 polymorphisms on	UGT1A1 gene promoter polymorphism ([TA]n repeat)	Increased risk of SCD complication: cholelithiasis (gallstones) and	Direct link between a non-globin promoter polymorphism and a major SCD

	cholelithiasis in sickle cell disease.		elevated serum bilirubin levels	complication
17600229	Confirmation of an association between the TNF(−308) promoter polymorphism and stroke risk in children with sickle cell anemia.	TNF−α gene promoter polymorphism (TNF(−308)G/A)	Increased risk of large vessel stroke (major SCD complication)	Direct link between a non-globin promoter polymorphism and a critical measure of SCD phenotypic severity
17894837	β-globin gene cluster polymorphisms are strongly associated with severity of HbE/β-thalassaemia.	XmnI polymorphism located 158-bp upstream to the G gamma gene	Modulation of HbF levels and HbE/β-thalassaemia severity	Explicit association of non-coding SNPs (polymorphisms) in XmnI 158-bp upstream to the G gamma gene to clinical disease severity and HbF levels
17916081	The β+IVS−I−6T→C mutation is the most frequent cause for the atypical β-thalassemia carrier phenotype in Portugal.	β-globin gene intronic splice site mutation (β+IVS−I−6T→C)+C94	Atypical β-thalassemia carrier phenotype	Direct link between an intronic (non-coding) mutation and an atypical, milder presentation of β-thalassemia
18697826	The HBS1L−MYB intergenic region on chromosome 6q23 is a quantitative trait locus controlling fetal haemoglobin level in carriers of β-thalassaemia.	HBS1L−MYB intergenic region QTL (non-coding SNPs)	Control of fetal haemoglobin (HbF) level (a severity modifier)	Direct link between a well-known non-coding QTL (defined by SNPs) in an intergenic region and the HbF modifier in β-thalassemia
20172753	Association of variant alleles of MBL2 gene with vasoocclusive crisis in children with sickle cell anemia.	MBL2 gene promoter -221 (alleles Y/X) and exon polymorphisms	Modification of SCA phenotype related to vasoocclusive crisis (VOC)	Direct link between non-coding MBL2 promoter polymorphisms and a major measure of SCD severity (VOC)
20351105	Placenta growth factor (PlGF), a novel inducer of plasminogen activator inhibitor-1 (PAI−1) in sickle cell disease (SCD).	PAI−1 gene promoter (HRE−1,HRE−2, and AP−1 sites).	SCD phenotype link: Increased PAI−1 (a prothrombotic state) due to non-coding PAI−1 promoter sites.	Although indirect, it links a major SCD complication (prothrombotic state) to the activity of a non-coding promoter region in a non-globin gene.
20472475	The XmnI (G)γ polymorphism influences hemoglobin F synthesis contrary to BCL11A and	Gγ-globin gene promoter SNP (XmnI) and intergenic/intronic SNPs (BCL11A and HBS1L−MYB QTLs).	HbF level (a major severity modifier) in β-thalassemia intermedia.	Direct comparison and finding a strong correlation between the non-coding XmnI SNP and a key severity

	HBS1L−MYB SNPs in a cohort of 57 β-thalassemia intermedia patients.			measure (HbF).
20709051	The effect of UGT1A1 promoter polymorphism on bilirubin response to hydroxyurea therapy in hemoglobinopathies.	UGT1A1 gene promoter polymorphism.	Bilirubin levels (a measure of hemolysis severity) and response to therapy.	Direct link between a non-globin promoter polymorphism and a measurable clinical severity factor (bilirubin/hemolysis) in hemoglobinopathy patients.
21144779	Xmn I polymorphism associated with concomitant activation of Gγ and Aγ globin gene transcription on a β0-thalassemia chromosome.	Gγ-globin gene promoter polymorphism (Xmn I C→T).	Moderately severe β0-thalassemia phenotype (despite total HbA absence).	Direct link between the non-coding XmnI SNP and the overall clinical severity (moderately severe phenotype).
21250885	β+-Thalassemia trait due to a novel mutation in the β-globin gene promoter: −26 (A>C) [HBB c.−76A>C].	β-globin gene promoter mutation (novel −26 (A>C)).	β+-Thalassemia trait (mild phenotype).	Direct link between a novel non-coding promoter mutation and the resultant, relatively mild β-thalassemia phenotype.
21757944	A 13-bp deletion in the 3′ untranslated region of the β-globin gene causes β-thalassemia major in compound heterozygosity with IVSII−1 mutation.	β-globin gene 3′ untranslated region (UTR) deletion (13-bp deletion).	β-Thalassemia major (severe phenotype).	Direct link between a UTR (non-coding) variant and the most severe clinical classification (β-thalassemia major).
21801233	The β-globin promoter −71 C>T mutation is a β+ thalassemic allele.	β-globin gene promoter mutation (novel −71 C>T).	β+ thalassemic allele (a classification of β-thalassemia severity/function).	Direct link between a non-coding promoter mutation and its effect on gene expression/severity classification.
22217218	Identification of a novel mutation in the β-globin gene 3′ untranslated region [+1,506 (A>C)] in a Japanese male with a heterozygous β-thalassemia phenotype.	β-globin gene 3′ untranslated region (UTR) mutation (novel +1,506 (A>C)).	Heterozygous β-thalassemia phenotype (severity classification).	Direct link between a novel UTR mutation (non-coding) and the resulting clinical phenotype.

22576309	Protein Z polymorphisms associated with vaso-occlusive crisis in young sickle cell disease patients.	Protein Z promoter SNPs (rs3024718,rs3024719,rs3024731) and intron SNP (rs3024735).	SCD complication: Vaso-occlusive crisis (VOC) (major severity measure).	Direct link between non-globin, non-coding SNPs (promoter and intronic) and a key measure of SCD phenotypic severity (VOC frequency).
22862814	Two novel mutations in the 3′ untranslated region of the β-globin gene that are associated with the mild phenotype of β-thalassemia.	β-globin gene 3′ untranslated region (UTR) mutations (HBB:c.∗+108A>G and HBB:c.∗+132C>T).	β-Thalassemia mild phenotype.	Direct link between novel non-coding UTR mutations and a specific, less severe clinical outcome.
22966170	Heme oxygenase-1 gene promoter polymorphism is associated with reduced incidence of acute chest syndrome among children with sickle cell disease.	Heme oxygenase−1 (HMOX1) gene promoter polymorphism (GT repeat SNP).	SCD complication: Acute chest syndrome (ACS) (a major severity measure).	Direct link between a non-globin promoter SNP (non-coding) and a critical, major measure of SCD phenotypic severity (ACS incidence).
23425204	Association in cis of the mutations +20 (C>T) in the 5′ untranslated region and IVS−II−745 (C>G) on the β-globin gene.	β-globin gene 5′ untranslated region (UTR) mutation (+20 (C>T)).	Association with mild β-thalassemia (severity classification).	Direct link between a UTR mutation (non-coding) and the resulting milder clinical phenotype.
23541515	The influence of the BCL11A polymorphism on the phenotype of patients with β-thalassemia could be affected by the β-globin LCR...	Intronic SNPs (in BCL11A); Locus Control Region (LCR) polymorphism.	Phenotype (Severity) and HbF variation in β-thalassemia.	Directly links BCL11A intronic SNPs and an LCR polymorphism to β-thalassemia phenotype and HbF levels, which modifies disease severity.
23556445	Genomic variation in the MAP3K5 gene is associated with β-thalassemia disease severity and hydroxyurea treatment efficacy.	Promoter short tandem repeat in MAP3K5; Intronic variants in MAP3K5.	Low HbF levels and a severe disease phenotype in β-hemoglobinopathy.	Directly links non-coding variants (promoter/intronic) in a modifier gene to HbF levels and disease severity.
23606168	Genetic modifiers of sickle cell anemia in the BABY HUG cohort: influence on laboratory and clinical phenotypes.	Intronic SNPs (BCL11A, HBS1L-MYB, XmnI); UGT1A1 promoter polymorphisms; α-thalassemia deletion.	Baseline HbF, serum bilirubin, and pain phenotype in SCD.	Links multiple classic non-coding variants (intronic, promoter, gene deletion) to HbF, bilirubin, and clinical phenotype (pain) in SCD.

23619273	Early complication in Sickle Cell Anemia children due to A(TA)nTAA polymorphism at the promoter of UGT1A1 gene.	Promoter polymorphism (A(TA)nTAA repeat) in UGT1A1.	Hyperbilirubinemia and gallstones formation (cholelithiasis) in SCD.	Directly links a promoter polymorphism to a measure of severity/complication (gallstones/hyperbilirubinemia) in SCD.
24060717	UGT1A1 promoter polymorphism associated with serum bilirubin level in Saudi patients with sickle cell disease.	Promoter polymorphism (A(TA)n repeat) in UGT1A1.	Serum bilirubin level and gallstones (cholelithiasis) in SCD.	Directly links a promoter polymorphism to hemolysis biomarkers (bilirubin) and cholelithiasis in SCD.
24065537	Association between clinical expression and molecular heterogeneity in β-thalassemia Tunisian patients.	Intronic SNP (XmnI polymorphism at-158 of the Gγ gene	Clinical expression (β-thalassemia major vs. intermedia).	Directly links the XmnI intronic polymorphism (a known HbF modifier) to the key phenotypic severity measure (β-thalassemia major vs. intermedia).
24168396	Genetic variation in CD36, HBA, NOS3 and VCAM1 is associated with chronic haemolysis level in sickle cell anaemia: a longitudinal study.	VCAM1 promoter haplotype; α-globin gene deletion (non-coding modifier).	Haemolysis biomarkers (LDH, total bilirubin, reticulocyte count).	Links non-coding variants (promoter haplotype, α-thalassemia deletion) to chronic hemolysis (a measure of severity) in SCD.
24204915	The effect of UGT1A1 promoter polymorphism in the development of hyperbilirubinemia and cholelithiasis in hemoglobinopathy patients.	Promoter polymorphism ((TA)n repeat) in UGT1A1.	Hyperbilirubinemia and cholelithiasis development in SCD and β-thalassemia.	Confirms the direct link between a promoter polymorphism and severity (complication/bilirubin) in both β-thalassemia and SCD.
24401016	Identification of three new nucleotide substitutions in the β-globin gene: laboratoristic approach and impact on genetic counselling for beta-thalassaemia.	Promoter/CAP site (HBB: c.-35 A>G); Intron 2 (HBB: c.316-373); 3' UTR (HBB: c.*+182 G>A).	Phenotypic expression in β-thalassemia.	Reports new non-coding variants (Promoter, Intronic, 3' UTR) in the β-globin gene and discusses their correlation with phenotypic expression/severity.
24614105	HBS1L-MYB intergenic variants modulate fetal	Intergenic variant rs66650371in Enhancer elements in HBS1L-	Elevated fetal hemoglobin (HbF) levels	Directly links intergenic/enhancer non-coding variants

	hemoglobin via long-range MYB enhancers.	MYB region.	and ameliorate sickle cell and β-thalassemia disease severity.	to HbF levels, a major modifier of severity in β-thalassemia and SCD.
24620945	Role of co-inherited Gilbert syndrome on hyperbilirubinemia in Indian beta thalassemia patients.	Promoter region (TA)n motifs of the bilirubin UGT1A1 gene.	Serum bilirubin levels in β-thalassemia (transfusion-dependent and intermedia).	Directly links a promoter polymorphism to a measure of severity (hyperbilirubinemia) in β-thalassemia.
25069958	Erythroid Krüppel-like factor-1, an iron-sensing protein, is a critical regulator of placenta growth factor, which is implicated in the vasculopathy of sickle cell disease.	PlGF promoter region (via EKLF binding). rs9376090, rs66650371, rs9399137, rs35786788, rs4895441, rs9389269, rs9402686, rs9494142, rs9494145 and rs948378 are associated with High-HbF.	Early mortality and iron overload markers in SCD.	Links gene regulation through its promoter region (a non-coding element) to a clinical severity measure (early mortality).
25657036	A novel promoter mutation (HBB: c.-75G>T) was identified as a cause of β(+)-thalassemia.	Promoter mutation (HBB: c.-75G>T).	β-thalassemia intermedia (β-TI) phenotype.	Directly links a novel promoter mutation (non-coding) to the overall phenotypic severity classification (β-TI).
25876995	Erythropoietin-mediated expression of placenta growth factor is regulated via activation of hypoxia-inducible factor-1α and post-transcriptionally by miR-214 in sickle cell disease.	Intronic microRNA (miR-214); PlGF promoter HREs.	Pulmonary hypertension (PHT) (SCD complication/severity).	Links a non-coding RNA (miR-214, located in an intron) and promoter HREs to a gene product (PlGF) and a major severity outcome (PHT) in SCD.
25928412	Genetic association of fetal-hemoglobin levels in individuals with sickle cell disease in Tanzania maps to conserved regulatory elements within the HBS1L-MYB intergenic region.	Intergenic/Enhancer regulatory elements within the HBS1L-MYB region.	Fetal-hemoglobin (HbF) levels in SCD.	Directly links non-coding regulatory elements in the intergenic region to HbF levels, a key measure of disease severity.
26291972	First Description of a β-Thalassemia Mutation, -86 (C>G) (HBB: c.-136C>G), in a Chinese Family.	Promoter mutation (HBB: c.-136C>G, in the CACCC box).	β(+)-thalassemia phenotype (a measure of severity).	Directly links a mutation in the promoter (CACCC box) of the β-globin gene to a resulting thalassemic

				phenotype/severity.
28871148	Editing an α-globin enhancer in primary human hematopoietic stem cells as a treatment for β-thalassemia.	Enhancer deletion (MCS-R2 α-globin enhancer).	Ameliorating the clinical severity (β-thalassemia corrected globin chain imbalance).	Although a gene-editing study, it directly establishes the functional link between deleting a non-coding element (enhancer) and correcting the pathology/severity of β-thalassemia.
28879539	Synergistic effect of two β globin gene cluster mutations leading to the hereditary persistence of fetal hemoglobin (HPFH) phenotype.	Promoter mutation (Novel Gγ promoter 5bp deletion)	Hereditary Persistence of Fetal Hemoglobin (HPFH) phenotype (High HbF levels)	Directly links a promoter deletion (non-coding) to significantly elevated HbF levels, which ameliorates β-thalassemia severity.
29227829	A long noncoding RNA from the HBS1L-MYB intergenic region on chr6q23 regulates human fetal hemoglobin expression.	Intergenic/Enhancer lncRNA (HMI-LNCRNA); Intergenic 3-bp deletion polymorphism (rs66650371).	Fetal hemoglobin (HbF) expression (A key modifier of disease severity).	Links a non-coding RNA transcribed from the HBS1L−MYB enhancer region to the regulation of HBG expression/HbF levels, directly impacting β-thalassemia and SCD severity.
29590102	Common α-globin variants modify hematologic and other clinical phenotypes in sickle cell trait and disease.	Enhancer SNP (rs11865131 in MCS-R2 α-globin enhancer); α-thalassemia deletion (-α3.7 deletion).	Stroke risk (major SCD complication/severity); Anemia; Chronic Kidney Disease (CKD).	Links a regulatory variant (SNP) in the α-globin enhancer and the α-thalassemia deletion (non-coding modifier) to major SCD clinical complications and anemia severity.
31688634	New Deletion at Promoter of HBG1 Gene in Sickle Cell Disease Patients With High HbF Level.	Promoter deletion (GCAG deletion at -273 in HBG1 promoter).	High HbF Level (a key modifier of SCD severity).	Directly links a novel promoter deletion (non-coding variant) to elevated HbF levels, which naturally ameliorates SCD severity.
31788855	HbS/β+ thalassemia: Really a mild disease? A National survey	Promoter mutation (A class of β+ mutations including promoter	Clinical Severity (SCD-related events	Links the β+ promoter mutation class to a milder

	from the AIEOP Sickle Cell Disease Study Group with genotype-phenotype correlation.	types).	like VOC, ACS, Stroke); Correlates with lower severity vs. IVS-I-110 group.	clinical phenotype (fewer severe events) compared to the IVS-I-110 splicing mutation.
32142096	Three Mexican Families with β thalassemia intermedia with different molecular basis.	3' UTR mutation (HBB: c.*132C>A).	β-thalassemia intermedia (β-TI) phenotype.	Directly links a novel 3' UTR mutation (non-coding variant) with the mutation c.118C > T (p.Gln39*) and a new duplication of the alpha globin gene cluster to the β-TI phenotype, a measure of reduced severity/transfusion independence.
34999313	VCAM1, HMOX1 and NOS3 differential endothelial expression may impact sickle cell anemia vasculopathy.	Promoter Haplotypes (VCAM1 promoter haplotypes).	Cerebral vasculopathy and severe hemolysis (major complication/severity measures) in SCA.	Directly links promoter haplotypes (non-coding variants) in a modifier gene (VCAM1) to the risk of a major SCD complication (vasculopathy) and severe hemolysis.
35095995	Whole Blood Transcriptome Analysis in Children with Sickle Cell Anemia.	Non-coding RNA SNPs (in RYR2 gene); Non-coding RNA (JHDM1D-AS1).	Fetal hemoglobin (HbF) regulation (key severity modifier).	Identifies SNPs in a novel non-coding RNA (RYR2) and links another non-coding RNA (JHDM1D-AS1) to the regulation of HBG mRNA levels/HbF expression, which influences SCD severity.
37857886	Iron overload induces dysplastic erythropoiesis and features of myelodysplasia in Nrf2-deficient mice.	Promoter SNP (NRF2 promoter SNP rs35652124).	Erythroid dysplasia (indicated by increased MCV); Iron overload (severity measure) in Hemochromatosis patients.	Links a promoter SNP in a modifier gene (NRF2) to clinical/anemia severity measures (MCV) in a related hemoglobinopathy patient group (HFE-Hemochromatosis) that shares pathophysiology (ineffective erythropoiesis/IOL)

				with β-thalassemia.
38293057	Structural Insights into the DNA-Binding Mechanism of BCL11A: The Integral Role of ZnF6.	Promoter mutations (γ-globin gene promoter mutations).	Increased HbF in adults (HPFH phenotype).	Directly mentions that rare γ-globin promoter mutations (non-coding variants) naturally perturb BCL11A binding, leading to increased HbF and the Hereditary Persistence of Fetal Hemoglobin (HPFH) phenotype, which ameliorates β-thalassemia/SCD severity.
38674403	Genetic Modifiers of Sickle Cell Anemia Phenotype in a Cohort of Angolan Children.	Intronic SNPs (Two SNPs in the intronic region of BCL11A on 2q16.1).	Decreasing HbF levels and Vaso-Occlusive Crises (VOC) (key severity measures).	Directly links intronic SNPs in the major modifier gene BCL11A to the major clinical severity outcome, VOC, and the primary biomarker, HbF, in a patient cohort.
40089598	In vivo deletion of a GWAS-identified Myb distal enhancer acts on Myb expression, globin switching, and clinical erythroid parameters in β-thalassemia.	Enhancer deletion (Myb distal enhancer, -84kb enhancer in human / -81kb in mouse).	Globin switching and clinical erythroid parameters in β-thalassemia (Severity model).	Directly establishes the in vivo function of a GWAS-identified non-coding enhancer in regulating a modifier gene (MYB), confirming its role in β-thalassemia severity (phenotype). This provides strong functional validation for natural enhancer variants.
40159794	Unusual Causes of β Thalassemia Trait: Discovery of another Three Novel SUPT5H Variants.	Intronic variant (c.307+1G>A in SUPT5H).	β thalassemia trait phenotype (hypochromic microcytic indices, increased HbA2 without globin gene mutation).	Directly links a novel intronic variant that alters splicing in the non-globin gene SUPT5H to the β-thalassemia phenotype/severity, which is outside the globin cluster.
20602015	Promoter region sequence differences	γ-globin gene Promoter SNPs (HBG2: -396_-	HbF levels.	Direct link between cis-acting promoter

	in the A and G γ globin genes of Brazilian sickle cell anemia patients.	391 del, -369 SNP; HBG1: -271 SNP).		variants (SNPs and a deletion) and the primary severity modifier (HbF) in SCD.
20808897	Disease-associated mutations that alter the RNA structural ensemble.	C33G SNP in the 5′ UTR of HBB	β-Thalassemia disease phenotype.	Identifies SNPs in UTRs that alter mRNA structure, suggesting a mechanism for phenotypic outcome in β-Thalassemia.
21264913	Severe sickle cell anemia is associated with increased plasma levels of TNF−R1 and VCAM−1.	SNPs (GWAS-identified) in VCAM1 (rs1041163) and ARFGEF2 (rs2273102).	SCA severity (mild vs. severe). VCAM1 (rs1041163) significantly associated with SCA severity (Bayes Factor = 3.5). ARFGEF2 (rs2273102)involved in endothelial release of TNF-R1 into circulation (Bayes Factor = 70	GWAS association linking SNPs in non-globin genes to the overall clinical severity of SCD.
26372199	Hereditary Persistence of Fetal Hemoglobin Caused by Single Nucleotide Promoter Mutations in Sickle Cell Trait and Hb SC Disease.	γ-globin gene Promoter Point Mutation.	High HbF levels (e.g., 38.0%).	Direct evidence of a naturally occurring γ-globin promoter mutation leading to HPFH and high HbF, a major ameliorating factor for SCD severity.
26977272	Impact of Mannose-Binding Protein Gene Polymorphisms in Omani Sickle Cell Disease Patients.	MBL2 Promoter SNPs and Exonic Haplotype.	VOC episodes (Vaso-occlusive crises).	Links non-globin gene promoter variants to a core clinical severity measure (VOCs) in SCD.
32447424	Functional polymorphisms of BCL11A and HBS1L−MYB genes affect both HbF level and clinical outcomes in a cohort of children with sickle cell anemia.	Noncoding SNPs independently associated with HbF levels: rs4671393 in BCL11A, rs9399137 in HMIP-2A, and rs4895441 in HMIP-2B	HbF level and clinical outcomes (severity).	Comprehensive association study directly linking established non-coding genetic modifier SNPs to HbF and overall clinical severity in SCD.

15710570	Association of UGT1A1 polymorphism with prevalence and age at onset of cholelithiasis in sickle cell anemia.	UGT1A1 promoter polymorphism ((TA)n repeats)	Major genetic risk factor modifying the frequency and age-at-onset of cholelithiasis (a major complication/severity measure) in SCA patients.	Non-globin non-coding variant modifying the complication-related severity of SCD.
15813858	Co-inheritance of Hb Sun Prairie mutation with a point mutation at 5'-UTR in the eastern Indian population.	5'-UTR point mutation (C→T)	Depression of translation due to conserved base in the 5'-UTR might explain the clinical severity	Direct link between a non-coding 5′−UTR mutation and clinical severity in α-globinopathy.
16044458	Nucleotide -88 (C→T) promoter mutation is a common β-thalassemia mutation in India	Promoter mutation (-88 C→T) and Polymorphism (XmnI Gγ)	Homozygotes are clinically mild with high Hb F (38.1-68.6%). Milder compound heterozygote phenotype associated with XmnI Gγ polymorphism.	Confirms the ameliorating role of a β-globin promoter mutation and the XmnI SNP on β-thalassemia severity.
16628735	UGT1A1 polymorphism outweighs the modest effect of deletional (-3.7 kb) α-thalassemia on cholelithogenesis in SCA.	UGT1A1 promoter polymorphism coinherited wiith α-Globin Gene Deletion (-3.7 kb)	α-thalassemia is associated with modest reduction in hemolysis (SCD severity measure).	Directly links the non-coding α-thalassemia deletion (modifier) to a reduction in SCD severity (hemolysis).
16704446	α-haemoglobin stabilising protein is a quantitative trait gene that modifies the phenotype of β-thalassaemia.	Promoter homopolymer variant (Tn) in the AHSP gene	Alters AHSP expression, a "relevant contributory factor in the haematological phenotype" of β-thalassaemia.	Non-globin non-coding variant modifying β-thalassemia severity (phenotype). Addresses "missing heritability".
19050890	The Hellenic type of nondeletional hereditary persistence of fetal hemoglobin results from a novel mutation (g.−109G→T) in the HBG2 gene promoter.	Promoter mutation HBG2:g.-109G>T	Results in elevated Hb F in adults (a measure that ameliorates β-thalassemia/SCD severity).	Direct link between a γ-globin promoter mutation and the severity-ameliorating factor HbF.

Supplementary Data 1: List of articles screened in the study. [1–527]

[1] J. Zeng, M.A. Nguyen, P. Liu, L.F. da Silva, S. Levesque, L.Y. Lin, D.G. Justus, K. Petri, K. Clement, S.N. Porter, A. Verma, N.R. Neri, T. Rosanwo, M.-F. Ciuculescu, D. Abriss, E. Mintzer, S.A. Maitland, S. Demirci, H.J. Cha, S.H. Orkin, J.F. Tisdale, D.A. Williams, L.J. Zhu, S.M. Pruett-Miller, L. Pinello, J.K. Joung, V. Pattanayak, J.P. Manis, M. Armant, D. Pellin, C. Brendel, S.A. Wolfe, D.E. Bauer, Gene editing without ex vivo culture evades genotoxicity in human hematopoietic stem cells, Cell Stem Cell 32 (2025) 191-208.e11. https://doi.org/10.1016/j.stem.2024.11.001.

[2] S. Xu, D. Liang, Q. Wang, Y. Cheng, D. Xie, Y. Gui, H. Zhang, C. Feng, F. Zhao, W. Ren, G. Sun, Y. Yang, L. Li, Y. Lai, B. Fu, Y. Lu, Z.J. Wang, Y. Wu, In vivo genome editing of human haematopoietic stem cells for treatment of blood disorders using mRNA delivery, Nat Biomed Eng (2025). https://doi.org/10.1038/s41551-025-01480-y.

[3] C. Wongborisuth, P. Innachai, C. Saisawang, A. Tubsuwan, N. Jearawiriyapaisarn, P. Kaewprommal, J. Piriyapongsa, W. Chiangjong, U. Anurathapan, D. Songdej, A. Tangprasittipap, S. Hongeng, Disrupting ZBTB7A or BCL11A binding sites reactivates fetal hemoglobin in erythroblasts from healthy and β0-thalassemia/HbE individuals, Sci Rep 15 (2025) 25580. https://doi.org/10.1038/s41598-025-10791-8.

[4] S. Panyasai, P. Prayalaw, K. Singha, S. Fucharoen, Molecular and hematological characteristics of two different δ-globin promoter variants, δ-276(A>G) and δ-77(T >C) among Thai, Burmese, and Laotian subjects, PeerJ 13 (2025) e19636. https://doi.org/10.7717/peerj.19636.

[5] C.D. Palani, A. Smith, X. Cao, B. Li, B.S. Pace, A. Starlard-Davenport, Cholesterol-conjugated miR-29b induces fetal haemoglobin expression via γ-globin promoter demethylation in the Townes mouse model for sickle cell anaemia, Br J Haematol 206 (2025) 1786–1795. https://doi.org/10.1111/bjh.20107.

[6] N.F.F. Nik Mohd Hasan, A. Achour, T. Koopmann, A. van Gammeren, J. van der Leeuw, H. Ceelie, D. Stieber, F. Baas, C.L. Harteveld, Unusual Causes of β Thalassemia Trait: Discovery of another Three Novel SUPT5H Variants, Hemoglobin 49 (2025) 145–148. https://doi.org/10.1080/03630269.2025.2484230.

[7] N. Maroofi, M.S.M. Maleki, M. Tahmasebi, H.R.K. Khorshid, Y. Modaberi, R. Najafipour, M. Banan, Detection of CRISPR/Cas9-Mediated Fetal Hemoglobin Reactivation in Erythroblasts Derived from Cord Blood-Hematopoietic Stem Cells, Mol Biotechnol 67 (2025) 1695–1706. https://doi.org/10.1007/s12033-024-01155-0.

[8] E. Marco, P. Sousa, T. Janoudi, E. de Dreuzy, J.M. Heath, R. Viswanathan, J.A. Zuris, G.M. Gotta, G. Giannoukos, S. Hansen, D.K. Wood, M.C. Walters, J.F. Tisdale, C.J. Wilson, K.-H. Chang, Nonclinical evaluation of renizgamglogene autogedtemcel for SCD and TDT, Mol Ther (2025) S1525-0016(25)00760–9. https://doi.org/10.1016/j.ymthe.2025.09.031.

[9] J. Khor, Y.L. Boo, SPTA1-Related Hereditary Spherocytosis: Novel Compound Heterozygous Mutations With Severe Clinical Manifestation, Cureus 17 (2025) e83724. https://doi.org/10.7759/cureus.83724.

[10] Y. Ilboudo, N. Brosseau, K.S. Lo, H. Belhaj, S. Moutereau, K. Marshall, M. Reid, A. Kutlar, A.E. Ashley-Koch, M.J. Telen, P. Joly, F. Galactéros, P. Bartolucci, G. Lettre, A replication study of novel fetal hemoglobin-associated genetic variants in sickle cell disease-only cohorts, Hum Mol Genet 34 (2025) 699–710. https://doi.org/10.1093/hmg/ddaf015.

[11] Y. Han, B. Gudmundsdottir, K.O. Gudmundsson, K.R. Roy, J. Tisdale, Y. Du, MLL1 complex is a critical regulator of fetal hemoglobin repression, bioRxiv (2025) 2025.03.24.645036. https://doi.org/10.1101/2025.03.24.645036.
[12] S.L. Grimm, M. Karki, K.A. Blum, J.-P. Bertocchio, R. He, D.N. Tripathi, N.M. Zacharias, J.M. Lebenthal, R.A. Sheth, P. Rao, G. Genovese, Z. Lu, R.C. Bast, D.R. Ingram, R. Lazcano, K.M. Wani, W.-L. Wang, A.J. Lazar, N.M. Tannir, C.L. Walker, C. Coarfa, P. Msaouel, CA-125 as a Biomarker in Renal Medullary Carcinoma: Integrated Molecular Profiling, Functional Characterization, and Prospective Clinical Validation, Clin Cancer Res 31 (2025) 1057–1068. https://doi.org/10.1158/1078-0432.CCR-24-3324.
[13] J. Gong, X. Xu, J. Zhu, Molecular genotyping of multi-system rare blood types in foreign blood donors based on DNA sequencing and its clinical significance, Open Med (Wars) 20 (2025) 20251234. https://doi.org/10.1515/med-2025-1234.
[14] Z.-Y. Gao, Z.-X. Yang, D.-H. Deng, F. Zhang, B. Yu, Y.-Y. Zhang, X. Lv, Y. Nakamura, A. Gong, T. Cheng, J.-P. Zhang, X.-B. Zhang, G2B: an optimized lentiviral vector with enhanced titer and β-globin expression for improved β-thalassemia gene therapy, Blood Sci 7 (2025) e00253. https://doi.org/10.1097/BS9.0000000000000253.
[15] L. Fontana, P. Martinucci, S. Amistadi, T. Felix, M. Mombled, A. Tachtsidi, G. Corre, A. Chalumeau, G. Hardouin, J. Martin, O. Romano, M. Amendola, P. Antoniou, A. Miccio, Multiplex base editing of BCL11A regulatory elements to treat sickle cell disease, Cell Rep Med 6 (2025) 102376. https://doi.org/10.1016/j.xcrm.2025.102376.
[16] E. Federti, D. Mattoscio, A. Recchiuti, A. Matte, M. Monti, F. Cozzolino, M. Iezzi, M. Ceci, A. Ghigo, E. Tolosano, A. Siciliano, J. Ceolan, V. Riccardi, E. Gremese, C. Brugnara, L. De Franceschi, 17(R)-Resolvin D1 protects against sickle cell-related inflammatory cardiomyopathy in humanized mice, Blood 145 (2025) 1915–1928. https://doi.org/10.1182/blood.2024024768.
[17] S. Demirci, J. Zeng, R. Palchaudhuri, C. Wu, D.M. Abraham, T.B. Hayal, K. Essawi, M.A. Nguyen, U. Stasula, R. Chu, A. Leonard, S.N. Porter, M.B.N. Khan, G. Hinojosa, N. Uchida, S. Hong, C.R. Lazzarotto, N.R. Neri, L.F. da Silva, D. Pellin, A. Verma, L. Lanieri, A. Bhat, K. Hammond, T. Tate, S.A. Maitland, F. Sheikhsaran, A.C. Bonifacino, A.E. Krouse, N.S. Linde, T. Engels, J. Golomb, S.Q. Tsai, S.M. Pruett-Miller, D.T. Scadden, C.E. Dunbar, S.A. Wolfe, R.E. Donahue, L.M. Olson, D.E. Bauer, J.F. Tisdale, BCL11A +58/+55 enhancer-editing facilitates HSPC engraftment and HbF induction in rhesus macaques conditioned with a CD45 antibody-drug conjugate, Cell Stem Cell 32 (2025) 209-226.e8. https://doi.org/10.1016/j.stem.2024.10.014.
[18] V. Deleuze, T. Stephen, M. Salma, C. Orfeo, R. Jorna, A. Maas, V. Barroca, M.-L. Arcangeli, C.-H. Lecellier, C. Andrieu-Soler, F. Grosveld, E. Soler, In vivo deletion of a GWAS-identified Myb distal enhancer acts on Myb expression, globin switching, and clinical erythroid parameters in β-thalassemia, Sci Rep 15 (2025) 8996. https://doi.org/10.1038/s41598-025-94222-8.
[19] Y. Amri, S.H. Fredj, R. Dabboubi, R. Othmani, C. Sahli, I. Baccouche, F. Ouali, T. Messaoud, Revealing silent alpha-thalassemia: characterization of novel HBA1 deletion and missense mutation in Tunisian families, Ann Hematol 104 (2025) 3173–3182. https://doi.org/10.1007/s00277-025-06320-2.
[20] S. Amistadi, L. Fontana, C. Magnoni, T. Felix, M.K. Charvin, P. Martinucci, C. Gautier, L. Greau, B. Bessières, P. Antoniou, O. Romano, E. Allemand, C. Mussolino, A. Miccio, Dissecting the epigenetic regulation of the fetal hemoglobin genes to unravel a novel therapeutic approach for β-hemoglobinopathies, Nucleic Acids Res 53 (2025) gkaf637. https://doi.org/10.1093/nar/gkaf637.

[21] C. Xi, C. Palani, M. Takezaki, H. Shi, A. Horuzsko, B.S. Pace, X. Zhu, Simvastatin-Mediated Nrf2 Activation Induces Fetal Hemoglobin and Antioxidant Enzyme Expression to Ameliorate the Phenotype of Sickle Cell Disease, Antioxidants (Basel) 13 (2024) 337. https://doi.org/10.3390/antiox13030337.
[22] Y. Wang, G. Myers, L. Yu, K. Deng, G. Balbin-Cuesta, S.A. Singh, Y. Guan, R. Khoriaty, J.D. Engel, TR4 and BCL11A repress γ-globin transcription via independent mechanisms, Blood 144 (2024) 2762–2772. https://doi.org/10.1182/blood.2024024599.
[23] T. Viennet, M. Yin, A. Jayaraj, W. Kim, Z.-Y.J. Sun, Y. Fujiwara, K. Zhang, D. Seruggia, H.-S. Seo, S. Dhe-Paganon, S.H. Orkin, H. Arthanari, Structural Insights into the DNA-Binding Mechanism of BCL11A: The Integral Role of ZnF6, bioRxiv (2024) 2024.01.17.576058. https://doi.org/10.1101/2024.01.17.576058.
[24] T. Viennet, M. Yin, A. Jayaraj, W. Kim, Z.-Y.J. Sun, Y. Fujiwara, K. Zhang, D. Seruggia, H.-S. Seo, S. Dhe-Paganon, S.H. Orkin, H. Arthanari, Structural insights into the DNA-binding mechanism of BCL11A: The integral role of ZnF6, Structure 32 (2024) 2276-2286.e4. https://doi.org/10.1016/j.str.2024.09.022.
[25] K. Prasad, N. Devaraju, A. George, N.S. Ravi, J. Paul, G. Mahalingam, V. Rajendiran, L. Panigrahi, V. Venkatesan, K. Lakhotiya, Y. Periyasami, A.A. Pai, Y. Nakamura, R. Kurita, P. Balasubramanian, S. Thangavel, S.R. Velayudhan, G.A. Newby, S. Marepally, A. Srivastava, K.M. Mohankumar, Precise correction of a spectrum of β-thalassemia mutations in coding and non-coding regions by base editors, Mol Ther Nucleic Acids 35 (2024) 102205. https://doi.org/10.1016/j.omtn.2024.102205.
[26] C.D. Palani, X. Zhu, M. Alagar, O.C. Attucks, B.S. Pace, Bach1 inhibitor HPP-D mediates γ-globin gene activation in sickle erythroid progenitors, Blood Cells Mol Dis 104 (2024) 102792. https://doi.org/10.1016/j.bcmd.2023.102792.
[27] K.A. Nguyen, A. Matte, R. Foresti, E. Federti, L. Kiger, C. Lefebvre, H. Hocini, Y. Pelinski, H. Kitagishi, L. Bencheikh, F. Pirenne, L. de Franceschi, R. Motterlini, P. Bartolucci, An oral carbon monoxide-releasing molecule protects against acute hyperhemolysis in sickle cell disease, Blood 143 (2024) 2544–2558. https://doi.org/10.1182/blood.2023023165.
[28] M. Mormin, L. Rigonnot, A. Chalumeau, A. Miccio, C. Fournier, S. Pajanissamy, M. Dewannieux, A. Galy, Cyclosporin H Improves the Transduction of CD34+ Cells with an Anti-Sickling Globin Vector, a Possible Therapeutic Approach for Sickle Cell Disease, Hum Gene Ther 35 (2024) 896–903. https://doi.org/10.1089/hum.2024.098.
[29] S. Lessard, P. Rimmelé, H. Ling, K. Moran, B. Vieira, Y.-D. Lin, G.M. Rajani, V. Hong, A. Reik, R. Boismenu, B. Hsu, M. Chen, B.M. Cockroft, N. Uchida, J. Tisdale, A. Alavi, L. Krishnamurti, M. Abedi, I. Galeon, D. Reiner, L. Wang, A. Ramezi, P. Rendo, M.C. Walters, D. Levasseur, R. Peters, T. Harris, A. Hicks, Zinc finger nuclease-mediated gene editing in hematopoietic stem cells results in reactivation of fetal hemoglobin in sickle cell disease, Sci Rep 14 (2024) 24298. https://doi.org/10.1038/s41598-024-74716-7.
[30] H.D. Langlands, D.K. Shoemark, A.M. Toye, Modulation of Antioxidant Enzyme Expression of In Vitro Culture-Derived Reticulocytes, Antioxidants (Basel) 13 (2024) 1070. https://doi.org/10.3390/antiox13091070.
[31] V. Katta, K. O’Keefe, Y. Li, T. Mayuranathan, C.R. Lazzarotto, R.K. Wood, R.M. Levine, A. Powers, K. Mayberry, G. Manquen, Y. Yao, J. Zhang, Y. Jang, N. Nimmagadda, E.A. Dempsey, G. Lee, N. Uchida, Y. Cheng, F. Fazio, T. Lockey, M. Meagher, A. Sharma, J.F. Tisdale, S. Zhou, J.S. Yen, M.J. Weiss, S.Q. Tsai, Development and IND-enabling studies of a novel Cas9 genome-edited autologous

CD34+ cell therapy to induce fetal hemoglobin for sickle cell disease, Mol Ther 32 (2024) 3433–3452. https://doi.org/10.1016/j.ymthe.2024.07.022.
[32] Y. Jiang, Y. Ye, X. Zhang, Y. Yu, L. Huang, X. Bao, X. Xu, Identification and characterization of CHD4-associated eRNA as a novel modulator of fetal hemoglobin levels in β-thalassemia, Biochem Biophys Res Commun 701 (2024) 149555. https://doi.org/10.1016/j.bbrc.2024.149555.
[33] C. Ginete, M. Delgadinho, B. Santos, A. Miranda, C. Silva, P. Guerreiro, E.R. Chimusa, M. Brito, Genetic Modifiers of Sickle Cell Anemia Phenotype in a Cohort of Angolan Children, Genes (Basel) 15 (2024) 469. https://doi.org/10.3390/genes15040469.
[34] G. Frati, M. Brusson, G. Sartre, B. Mlayah, T. Felix, A. Chalumeau, P. Antoniou, G. Hardouin, J.-P. Concordet, O. Romano, G. Turchiano, A. Miccio, Safety and efficacy studies of CRISPR-Cas9 treatment of sickle cell disease highlights disease-specific responses, Mol Ther 32 (2024) 4337–4352. https://doi.org/10.1016/j.ymthe.2024.07.015.
[35] H. Frangoul, F. Locatelli, A. Sharma, M. Bhatia, M. Mapara, L. Molinari, D. Wall, R.I. Liem, P. Telfer, A.J. Shah, M. Cavazzana, S. Corbacioglu, D. Rondelli, R. Meisel, L. Dedeken, S. Lobitz, M. de Montalembert, M.H. Steinberg, M.C. Walters, M.J. Eckrich, S. Imren, L. Bower, C. Simard, W. Zhou, F. Xuan, P.K. Morrow, W.E. Hobbs, S.A. Grupp, CLIMB SCD-121 Study Group, Exagamglogene Autotemcel for Severe Sickle Cell Disease, N Engl J Med 390 (2024) 1649–1662. https://doi.org/10.1056/NEJMoa2309676.
[36] T.L. Duarte, M. Lopes, M. Oliveira, A.G. Santos, C. Vasco, J.P. Reis, A.R. Antunes, A. Gonçalves, S. Chacim, C. Oliveira, B. Porto, M.J. Teles, A.C. Moreira, A.M.N. Silva, R. Schwessinger, H. Drakesmith, R. Henrique, G. Porto, D. Duarte, Iron overload induces dysplastic erythropoiesis and features of myelodysplasia in Nrf2-deficient mice, Leukemia 38 (2024) 96–108. https://doi.org/10.1038/s41375-023-02067-9.
[37] S. Demirci, M.B.N. Khan, G. Hinojosa, A. Le, A. Leonard, K. Essawi, B. Gudmundsdottir, X. Liu, J. Zeng, Z. Inam, R. Chu, N. Uchida, D. Araki, E. London, H. Butt, S.A. Maitland, D.E. Bauer, S.A. Wolfe, A. Larochelle, J.F. Tisdale, Ex vivo culture resting time impacts transplantation outcomes of genome-edited human hematopoietic stem and progenitor cells in xenograft mouse models, Cytotherapy 26 (2024) 641–648. https://doi.org/10.1016/j.jcyt.2024.02.011.
[38] L.-Z. Chen, T.-Z. Yan, J. Huang, Q.-Y. Zhong, X. Qin, N. Tang, S.-Q. Luo, [Molecular Diagnosis and Pedigree Analysis of Rare Mutations in Non-coding Region of HBA2 Gene], Zhongguo Shi Yan Xue Ye Xue Za Zhi 32 (2024) 940–944. https://doi.org/10.19746/j.cnki.issn.1009-2137.2024.03.044.
[39] P.B. Chandraprabha, M.K.K. Azhagiri, V. Venkatesan, W. Magis, K. Prasad, S. Suresh, A.A. Pai, S. Marepally, A. Srivastava, K.M. Mohankumar, D.I.K. Martin, S. Thangavel, Enhanced fetal hemoglobin production via dual-beneficial mutation editing of the HBG promoter in hematopoietic stem and progenitor cells for β-hemoglobinopathies, Stem Cell Res Ther 15 (2024) 504. https://doi.org/10.1186/s13287-024-04117-0.
[40] S.K. Appiah, C. Nkansah, G. Abbam, F. Osei-Boakye, K. Mensah, S.B. Bani, S. Chemogo, L. Sarpong, T.G. Addae, D.B. Sefa, R.A. Croffien, L. Adom, R.O.A. Rauf, F. Boadu, G.A. Amoah, E.F. Chukwurah, Molecular characterization of HAMP rs10421768 gene and phenotypic expression of hepcidin; a case-control study among sickle cell anaemia patients in Ghana, PLoS One 19 (2024) e0306194. https://doi.org/10.1371/journal.pone.0306194.

[41] G. Zhou, D. Lu, Proteomics screening uncovers HMGA1 as a promising negative regulator for γ-globin expression in response to decreased β-globin levels, J Proteomics 286 (2023) 104957. https://doi.org/10.1016/j.jprot.2023.104957.
[42] J. Zeng, M.A. Nguyen, P. Liu, L. Ferreira da Silva, L.Y. Lin, D.G. Justus, K. Petri, K. Clement, S.N. Porter, A. Verma, N.R. Neri, T. Rosanwo, M.-F. Ciuculescu, D. Abriss, E. Mintzer, S.A. Maitland, S. Demirci, J.F. Tisdale, D.A. Williams, L.J. Zhu, S.M. Pruett-Miller, L. Pinello, J.K. Joung, V. Pattanayak, J.P. Manis, M. Armant, D. Pellin, C. Brendel, S.A. Wolfe, D.E. Bauer, Gene editing without ex vivo culture evades genotoxicity in human hematopoietic stem cells, bioRxiv (2023) 2023.05.27.542323. https://doi.org/10.1101/2023.05.27.542323.
[43] G. Ureña-Bailén, M. Block, T. Grandi, F. Aivazidou, J. Quednau, D. Krenz, A. Daniel-Moreno, A. Lamsfus-Calle, T. Epting, R. Handgretinger, S. Wild, M. Mezger, Automated Good Manufacturing Practice-Compatible CRISPR-Cas9 Editing of Hematopoietic Stem and Progenitor Cells for Clinical Treatment of β-Hemoglobinopathies, CRISPR J 6 (2023) 5–16. https://doi.org/10.1089/crispr.2022.0086.
[44] M. Simbula, M.F. Manchinu, M. Mingoia, M. Pala, I. Asunis, C.A. Caria, L. Perseu, M. Shah, M. Crossley, P. Moi, M.S. Ristaldi, miR-365-3p mediates BCL11A and SOX6 erythroid-specific coregulation: A new player in HbF activation, Mol Ther Nucleic Acids 34 (2023) 102025. https://doi.org/10.1016/j.omtn.2023.09.002.
[45] A. Sharma, J.-J. Boelens, M. Cancio, J.S. Hankins, P. Bhad, M. Azizy, A. Lewandowski, X. Zhao, S. Chitnis, R. Peddinti, Y. Zheng, N. Kapoor, F. Ciceri, T. Maclachlan, Y. Yang, Y. Liu, J. Yuan, U. Naumann, V.W.C. Yu, S.C. Stevenson, S. De Vita, J.L. LaBelle, CRISPR-Cas9 Editing of the HBG1 and HBG2 Promoters to Treat Sickle Cell Disease, N Engl J Med 389 (2023) 820–832. https://doi.org/10.1056/NEJMoa2215643.
[46] S. Shang, X. Li, A. Azzo, T. Truong, M. Dozmorov, C. Lyons, A.K. Manna, D.C. Williams, G.D. Ginder, MBD2a-NuRD binds to the methylated γ-globin gene promoter and uniquely forms a complex required for silencing of HbF expression, Proc Natl Acad Sci U S A 120 (2023) e2302254120. https://doi.org/10.1073/pnas.2302254120.
[47] S.A. Peslak, S. Demirci, V. Chandra, B. Ryu, S.K. Bhardwaj, J. Jiang, J.W. Rupon, R.E. Throm, N. Uchida, A. Leonard, K. Essawi, A.C. Bonifacino, A.E. Krouse, N.S. Linde, R.E. Donahue, F. Ferrara, M. Wielgosz, O. Abdulmalik, N. Hamagami, P. Germino-Watnick, A. Le, R. Chu, M. Hinds, M.J. Weiss, W. Tong, J.F. Tisdale, G.A. Blobel, Forced enhancer-promoter rewiring to alter gene expression in animal models, Mol Ther Nucleic Acids 31 (2023) 452–465. https://doi.org/10.1016/j.omtn.2023.01.016.
[48] T. Mayuranathan, G.A. Newby, R. Feng, Y. Yao, K.D. Mayberry, C.R. Lazzarotto, Y. Li, R.M. Levine, N. Nimmagadda, E. Dempsey, G. Kang, S.N. Porter, P.A. Doerfler, J. Zhang, Y. Jang, J. Chen, H.W. Bell, M. Crossley, S.V. Bhoopalan, A. Sharma, J.F. Tisdale, S.M. Pruett-Miller, Y. Cheng, S.Q. Tsai, D.R. Liu, M.J. Weiss, J.S. Yen, Potent and uniform fetal hemoglobin induction via base editing, Nat Genet 55 (2023) 1210–1220. https://doi.org/10.1038/s41588-023-01434-7.
[49] C. Li, H. Shin, D. Bhavanasi, M. Liu, X. Yu, S.A. Peslak, X. Liu, J.R. Alvarez-Dominguez, G.A. Blobel, B.D. Gregory, J. Huang, P.S. Klein, Expansion of human hematopoietic stem cells by inhibiting translation, bioRxiv (2023) 2023.11.28.568925. https://doi.org/10.1101/2023.11.28.568925.
[50] V.M. Lazaris, E. Simantirakis, E.F. Stavrou, M. Verras, A. Sgourou, M.K. Keramida, G. Vassilopoulos, A. Athanassiadou, Non-Viral Episomal Vector Mediates Efficient

Gene Transfer of the β-Globin Gene into K562 and Human Haematopoietic Progenitor Cells, Genes (Basel) 14 (2023) 1774. https://doi.org/10.3390/genes14091774.
[51] L. Koniali, C. Flouri, M.I. Kostopoulou, N.Y. Papaioannou, P.L. Papasavva, B. Naiisseh, C. Stephanou, A. Demetriadou, M. Sitarou, S. Christou, M.N. Antoniou, M. Kleanthous, P. Patsali, C.W. Lederer, Evaluation of Mono- and Bi-Functional GLOBE-Based Vectors for Therapy of β-Thalassemia by HBBAS3 Gene Addition and Mutation-Specific RNA Interference, Cells 12 (2023) 2848. https://doi.org/10.3390/cells12242848.
[52] Y. Inostroza-Nieves, A. Rivera, J.R. Romero, Blockade of endothelin-1 receptor B regulates molecules of the major histocompatibility complex in sickle cell disease, Front Immunol 14 (2023) 1124269. https://doi.org/10.3389/fimmu.2023.1124269.
[53] V. Ibanez, K. Vaitkus, M.A. Ruiz, Z. Lei, M. Maienschein-Cline, Z. Arbieva, D. Lavelle, Effect of the LSD1 inhibitor RN-1 on γ-globin and global gene expression during erythroid differentiation in baboons (Papio anubis), PLoS One 18 (2023) e0289860. https://doi.org/10.1371/journal.pone.0289860.
[54] R.P. Hebbel, L. Milbauer, P. Wei, A novel promoter of endothelial dysfunction in African Americans: Relevance to sickle cell anaemia, Br J Haematol 203 (2023) e71–e73. https://doi.org/10.1111/bjh.18993.
[55] S.M. Hassan, A. Alrawas, L. Al Khanbashi, Y. Wali, Homozygous mild beta-thalassaemia promoter transversion -71 C>T HBB:c.-121 C>T, BMJ Case Rep 16 (2023) e254416. https://doi.org/10.1136/bcr-2022-254416.
[56] A. Finotti, J. Gasparello, C. Zuccato, L.C. Cosenza, E. Fabbri, N. Bianchi, R. Gambari, Effects of Mithramycin on BCL11A Gene Expression and on the Interaction of the BCL11A Transcriptional Complex to γ-Globin Gene Promoter Sequences, Genes (Basel) 14 (2023) 1927. https://doi.org/10.3390/genes14101927.
[57] S. Cancellieri, J. Zeng, L.Y. Lin, M. Tognon, M.A. Nguyen, J. Lin, N. Bombieri, S.A. Maitland, M.-F. Ciuculescu, V. Katta, S.Q. Tsai, M. Armant, S.A. Wolfe, R. Giugno, D.E. Bauer, L. Pinello, Human genetic diversity alters off-target outcomes of therapeutic gene editing, Nat Genet 55 (2023) 34–43. https://doi.org/10.1038/s41588-022-01257-y.
[58] M. Brusson, A. Chalumeau, P. Martinucci, O. Romano, T. Felix, V. Poletti, S. Scaramuzza, S. Ramadier, C. Masson, G. Ferrari, F. Mavilio, M. Cavazzana, M. Amendola, A. Miccio, Novel lentiviral vectors for gene therapy of sickle cell disease combining gene addition and gene silencing strategies, Mol Ther Nucleic Acids 32 (2023) 229–246. https://doi.org/10.1016/j.omtn.2023.03.012.
[59] M.Y. Boontanrart, E. Mächler, S. Ponta, J.C. Nelis, V.G. Preiano, J.E. Corn, Engineering of the endogenous HBD promoter increases HbA2, Elife 12 (2023) e85258. https://doi.org/10.7554/eLife.85258.
[60] A.M. Agarwal, V. McMurty, A.L. Clayton, A. Bolia, N.S. Reading, C. Mani, J.L. Patel, A. Rets, Clinical utility of targeted next-generation sequencing panel in routine diagnosis of hereditary hemolytic anemia: A national reference laboratory experience, Eur J Haematol 110 (2023) 688–695. https://doi.org/10.1111/ejh.13951.
[61] J. Zhu, H. Li, W. Aerbajinai, C. Kumkhaek, M. Pirooznia, A. Saxena, P. Dagur, K. Chin, G.P. Rodgers, Kruppel-like factor 1-GATA1 fusion protein improves the sickle cell disease phenotype in mice both in vitro and in vivo, Blood 140 (2022) 2276–2289. https://doi.org/10.1182/blood.2021014877.
[62] C. Wu, X. Wang, W. Zhen, Y. Nie, Y. Li, P. Yuan, Q. Liu, S. Guo, Z. Shen, B. Zheng, Z. Hu, SICKLE modulates lateral root development by promoting degradation of lariat intronic RNA, Plant Physiol 190 (2022) 548–561. https://doi.org/10.1093/plphys/kiac301.

[63] Y.-J. Wen, Q.-X. Yu, F. Jiang, D.-Z. Li, Identification of a Novel Mutation in the 3' Untranslated Region of the β-Globin Gene (HBB:c.*132C>G) in a Chinese Family, Hemoglobin 46 (2022) 347–350. https://doi.org/10.1080/03630269.2023.2176320.
[64] S.K. Topfer, R. Feng, P. Huang, L.C. Ly, G.E. Martyn, G.A. Blobel, M.J. Weiss, K.G.R. Quinlan, M. Crossley, Disrupting the adult globin promoter alleviates promoter competition and reactivates fetal globin gene expression, Blood 139 (2022) 2107–2118. https://doi.org/10.1182/blood.2021014205.
[65] M. Silva, A. Coelho, S. Vargas, P. Faustino, VCAM1, HMOX1 and NOS3 differential endothelial expression may impact sickle cell anemia vasculopathy, Blood Cells Mol Dis 93 (2022) 102639. https://doi.org/10.1016/j.bcmd.2021.102639.
[66] N.S. Ravi, B. Wienert, S.K. Wyman, H.W. Bell, A. George, G. Mahalingam, J.T. Vu, K. Prasad, B.P. Bandlamudi, N. Devaraju, V. Rajendiran, N. Syedbasha, A.A. Pai, Y. Nakamura, R. Kurita, M. Narayanasamy, P. Balasubramanian, S. Thangavel, S. Marepally, S.R. Velayudhan, A. Srivastava, M.A. DeWitt, M. Crossley, J.E. Corn, K.M. Mohankumar, Identification of novel HPFH-like mutations by CRISPR base editing that elevate the expression of fetal hemoglobin, Elife 11 (2022) e65421. https://doi.org/10.7554/eLife.65421.
[67] Y. Pan, L. Xu, H. Huang, Expression, functional mechanism and therapy application of long noncoding RNA in β-thalassemia, Zhong Nan Da Xue Xue Bao Yi Xue Ban 47 (2022) 252–257. https://doi.org/10.11817/j.issn.1672-7347.2022.210411.
[68] S. Mehta, A. Buyanbat, Y. Kai, O. Karayel, S.R. Goldman, D. Seruggia, K. Zhang, Y. Fujiwara, K.A. Donovan, Q. Zhu, H. Yang, B. Nabet, N.S. Gray, M. Mann, E.S. Fischer, K. Adelman, S.H. Orkin, Temporal resolution of gene derepression and proteome changes upon PROTAC-mediated degradation of BCL11A protein in erythroid cells, Cell Chem Biol 29 (2022) 1273-1287.e8. https://doi.org/10.1016/j.chembiol.2022.06.007.
[69] N.H. Lopez, B. Li, C. Palani, U. Siddaramappa, M. Takezaki, H. Xu, W. Zhi, B.S. Pace, Salubrinal induces fetal hemoglobin expression via the stress-signaling pathway in human sickle erythroid progenitors and sickle cell disease mice, PLoS One 17 (2022) e0261799. https://doi.org/10.1371/journal.pone.0261799.
[70] T. Li, H. Li, H. Lian, P. Song, Y. Wang, J. Duan, Z. Song, Y. Cao, D. Xu, J. Li, H. Zhang, SICKLE represses photomorphogenic development of Arabidopsis seedlings via HY5- and PIF4-mediated signaling, J Integr Plant Biol 64 (2022) 1706–1723. https://doi.org/10.1111/jipb.13329.
[71] L.H. Junker, B. Li, X. Zhu, S. Koti, R.E. Cerbone, C.L. Hendrick, J. Sangerman, S. Perrine, B.S. Pace, Novel histone deacetylase inhibitor CT-101 induces γ-globin gene expression in sickle erythroid progenitors with targeted epigenetic effects, Blood Cells Mol Dis 93 (2022) 102626. https://doi.org/10.1016/j.bcmd.2021.102626.
[72] F. Jiang, G.-L. Chen, J. Li, X.-W. Tang, D.-Z. Li, β-Thalassemia Intermedia Caused by the β-Globin Gene 3' Untranslated Region: Another Case Report, Hemoglobin 46 (2022) 137–139. https://doi.org/10.1080/03630269.2022.2086135.
[73] P. Huriez, C. Ourghanlian, K. Razazi, W. Vindrios, A. Hulin, R. Lepeule, A. Habibi, S. Gallien, Probenecid, an old β-lactams pharmacokinetic enhancer for a renewed use: A retrospective study, Infect Dis Now 52 (2022) 273–279. https://doi.org/10.1016/j.idnow.2022.05.006.
[74] I. Germano, B. Santos, M. Delgadinho, C. Ginete, P. Lopes, A.P. Arez, M. Brito, P. Faustino, Genetic modulation of anemia severity, hemolysis level, and hospitalization rate in Angolan children with Sickle Cell Anemia, Mol Biol Rep 49 (2022) 10347–10356. https://doi.org/10.1007/s11033-022-07831-1.

[75] R. Feng, T. Mayuranathan, P. Huang, P.A. Doerfler, Y. Li, Y. Yao, J. Zhang, L.E. Palmer, K. Mayberry, G.E. Christakopoulos, P. Xu, C. Li, Y. Cheng, G.A. Blobel, M.C. Simon, M.J. Weiss, Activation of γ-globin expression by hypoxia-inducible factor 1α, Nature 610 (2022) 783–790. https://doi.org/10.1038/s41586-022-05312-w.

[76] E. Drakopoulou, M. Georgomanoli, C.W. Lederer, F. Panetsos, M. Kleanthous, E. Voskaridou, D. Valakos, E. Papanikolaou, N.P. Anagnou, The Optimized γ-Globin Lentiviral Vector GGHI-mB-3D Leads to Nearly Therapeutic HbF Levels In Vitro in CD34+ Cells from Sickle Cell Disease Patients, Viruses 14 (2022) 2716. https://doi.org/10.3390/v14122716.

[77] W. Chauhan, R. Fatma, Z. Zaka-Ur-Rab, M. Afzal, Direct sequencing of β-globin gene reveals a rare combination of two exonic and two intronic variants in a β-thalassemia major patient: a case report, J Med Case Rep 16 (2022) 362. https://doi.org/10.1186/s13256-022-03605-2.

[78] R. Archana, C. Vidya, N. Sumithra, M. Jyothi, R. Sanil, Arab-Indian -530 ß-distal promoter haplotype and sickle/Hb D heterozygosis in Badagas of Nilgiris: is it suggestive of Harappan origin?, J Genet 101 (2022) 17.

[79] P. Allard, N. Alhaj, S. Lobitz, H. Cario, A. Jarisch, R. Grosse, L. Oevermann, D. Hakimeh, L. Tagliaferri, E. Kohne, A. Kopp-Schneider, A.E. Kulozik, J.B. Kunz, Genetic modifiers of fetal hemoglobin affect the course of sickle cell disease in patients treated with hydroxyurea, Haematologica 107 (2022) 1577–1588. https://doi.org/10.3324/haematol.2021.278952.

[80] L. Zhong, Y. Wang, W. Lin, Z. Yao, J. Zhang, H. Xu, P. Feng, L. Xu, Prenatal Genetic Counseling in a Chinese Pregnant Woman With Rare Thalassemia: A Case Report, Front Genet 12 (2021) 670168. https://doi.org/10.3389/fgene.2021.670168.

[81] Y. Yang, R. Ren, L.C. Ly, J.R. Horton, F. Li, K.G.R. Quinlan, M. Crossley, Y. Shi, X. Cheng, Structural basis for human ZBTB7A action at the fetal globin promoter, Cell Rep 36 (2021) 109759. https://doi.org/10.1016/j.celrep.2021.109759.

[82] S. Uçucu, T. Karabıyık, F.M. Azik, IVS-II-16 (G>C) (HBB: c.315+16G>C) or IVS-II-666 (C>T) (HBB: c.316-185C>T) Mutations Trigger an Hb S (HBB: c.20A>T)/β+-Thalassemia Phenotype in an Hb S Trait Patient, Hemoglobin 45 (2021) 225–227. https://doi.org/10.1080/03630269.2021.1965620.

[83] N. Uchida, F. Ferrara, C.M. Drysdale, M. Yapundich, J. Gamer, T. Nassehi, J. DiNicola, Y. Shibata, M. Wielgosz, Y.-S. Kim, M. Bauler, R.E. Throm, J.J. Haro-Mora, S. Demirci, A.C. Bonifacino, A.E. Krouse, N.S. Linde, R.E. Donahue, B. Ryu, J.F. Tisdale, Sustained fetal hemoglobin induction in vivo is achieved by BCL11A interference and coexpressed truncated erythropoietin receptor, Sci Transl Med 13 (2021) eabb0411. https://doi.org/10.1126/scitranslmed.abb0411.

[84] D. Taliun, D.N. Harris, M.D. Kessler, J. Carlson, Z.A. Szpiech, R. Torres, S.A.G. Taliun, A. Corvelo, S.M. Gogarten, H.M. Kang, A.N. Pitsillides, J. LeFaive, S.-B. Lee, X. Tian, B.L. Browning, S. Das, A.-K. Emde, W.E. Clarke, D.P. Loesch, A.C. Shetty, T.W. Blackwell, A.V. Smith, Q. Wong, X. Liu, M.P. Conomos, D.M. Bobo, F. Aguet, C. Albert, A. Alonso, K.G. Ardlie, D.E. Arking, S. Aslibekyan, P.L. Auer, J. Barnard, R.G. Barr, L. Barwick, L.C. Becker, R.L. Beer, E.J. Benjamin, L.F. Bielak, J. Blangero, M. Boehnke, D.W. Bowden, J.A. Brody, E.G. Burchard, B.E. Cade, J.F. Casella, B. Chalazan, D.I. Chasman, Y.-D.I. Chen, M.H. Cho, S.H. Choi, M.K. Chung, C.B. Clish, A. Correa, J.E. Curran, B. Custer, D. Darbar, M. Daya, M. de Andrade, D.L. DeMeo, S.K. Dutcher, P.T. Ellinor, L.S. Emery, C. Eng, D. Fatkin, T. Fingerlin, L. Forer, M. Fornage, N. Franceschini, C. Fuchsberger, S.M. Fullerton, S. Germer, M.T. Gladwin, D.J. Gottlieb, X. Guo, M.E. Hall, J. He, N.L. Heard-Costa, S.R. Heckbert, M.R. Irvin, J.M. Johnsen, A.D. Johnson, R. Kaplan, S.L.R. Kardia, T. Kelly,

S. Kelly, E.E. Kenny, D.P. Kiel, R. Klemmer, B.A. Konkle, C. Kooperberg, A. Köttgen, L.A. Lange, J. Lasky-Su, D. Levy, X. Lin, K.-H. Lin, C. Liu, R.J.F. Loos, L. Garman, R. Gerszten, S.A. Lubitz, K.L. Lunetta, A.C.Y. Mak, A. Manichaikul, A.K. Manning, R.A. Mathias, D.D. McManus, S.T. McGarvey, J.B. Meigs, D.A. Meyers, J.L. Mikulla, M.A. Minear, B.D. Mitchell, S. Mohanty, M.E. Montasser, C. Montgomery, A.C. Morrison, J.M. Murabito, A. Natale, P. Natarajan, S.C. Nelson, K.E. North, J.R. O'Connell, N.D. Palmer, N. Pankratz, G.M. Peloso, P.A. Peyser, J. Pleiness, W.S. Post, B.M. Psaty, D.C. Rao, S. Redline, A.P. Reiner, D. Roden, J.I. Rotter, I. Ruczinski, C. Sarnowski, S. Schoenherr, D.A. Schwartz, J.-S. Seo, S. Seshadri, V.A. Sheehan, W.H. Sheu, M.B. Shoemaker, N.L. Smith, J.A. Smith, N. Sotoodehnia, A.M. Stilp, W. Tang, K.D. Taylor, M. Telen, T.A. Thornton, R.P. Tracy, D.J. Van Den Berg, R.S. Vasan, K.A. Viaud-Martinez, S. Vrieze, D.E. Weeks, B.S. Weir, S.T. Weiss, L.-C. Weng, C.J. Willer, Y. Zhang, X. Zhao, D.K. Arnett, A.E. Ashley-Koch, K.C. Barnes, E. Boerwinkle, S. Gabriel, R. Gibbs, K.M. Rice, S.S. Rich, E.K. Silverman, P. Qasba, W. Gan, NHLBI Trans-Omics for Precision Medicine (TOPMed) Consortium, G.J. Papanicolaou, D.A. Nickerson, S.R. Browning, M.C. Zody, S. Zöllner, J.G. Wilson, L.A. Cupples, C.C. Laurie, C.E. Jaquish, R.D. Hernandez, T.D. O'Connor, G.R. Abecasis, Sequencing of 53,831 diverse genomes from the NHLBI TOPMed Program, Nature 590 (2021) 290–299. https://doi.org/10.1038/s41586-021-03205-y.

[85] A. Sen, V. Seenappa, P. Chakrabarti, T.K. Dolai, First Report of the 3'-Untranslated Region +1506 (A>C) [NM_000518.5: c.*32A>C] mutation on the β-Globin Gene in the Indian Population, Hemoglobin 45 (2021) 325–328. https://doi.org/10.1080/03630269.2021.2011314.

[86] C. Samuelson, S. Radtke, H. Zhu, M. Llewellyn, E. Fields, S. Cook, M.-L.W. Huang, K.R. Jerome, H.-P. Kiem, O. Humbert, Multiplex CRISPR/Cas9 genome editing in hematopoietic stem cells for fetal hemoglobin reinduction generates chromosomal translocations, Mol Ther Methods Clin Dev 23 (2021) 507–523. https://doi.org/10.1016/j.omtm.2021.10.008.

[87] M. Rafat, Z. Allamehzadeh, M. Shekari, M. Afsa, K. Malekzadeh, The Effect of HBB: c.-121C>T Variant [-71 (C>T)] on the β-Globin Promoter: Case Series Study, Hemoglobin 45 (2021) 234–238. https://doi.org/10.1080/03630269.2021.1955707.

[88] M. Mingoia, C.A. Caria, L. Ye, I. Asunis, M.F. Marongiu, L. Manunza, M.C. Sollaino, J. Wang, A. Cabriolu, R. Kurita, Y. Nakamura, F. Cucca, Y.W. Kan, M.G. Marini, P. Moi, Induction of therapeutic levels of HbF in genome-edited primary β0 39-thalassaemia haematopoietic stem and progenitor cells, Br J Haematol 192 (2021) 395–404. https://doi.org/10.1111/bjh.17167.

[89] X. Li, M. Chen, B. Liu, P. Lu, X. Lv, X. Zhao, S. Cui, P. Xu, Y. Nakamura, R. Kurita, B. Chen, D.C.S. Huang, D.-P. Liu, M. Liu, Q. Zhao, Transcriptional silencing of fetal hemoglobin expression by NonO, Nucleic Acids Res 49 (2021) 9711–9723. https://doi.org/10.1093/nar/gkab671.

[90] C. Li, H. Wang, A. Georgakopoulou, S. Gil, E. Yannaki, A. Lieber, In Vivo HSC Gene Therapy Using a Bi-modular HDAd5/35++ Vector Cures Sickle Cell Disease in a Mouse Model, Mol Ther 29 (2021) 822–837. https://doi.org/10.1016/j.ymthe.2020.09.001.

[91] P. Himadewi, X.Q.D. Wang, F. Feng, H. Gore, Y. Liu, L. Yu, R. Kurita, Y. Nakamura, G.P. Pfeifer, J. Liu, X. Zhang, 3'HS1 CTCF binding site in human β-globin locus regulates fetal hemoglobin expression, Elife 10 (2021) e70557. https://doi.org/10.7554/eLife.70557.

[92] P. Hariharan, M. Gorivale, P. Sawant, P. Mehta, A. Nadkarni, Significance of genetic modifiers of hemoglobinopathies leading towards precision medicine, Sci Rep 11 (2021) 20906. https://doi.org/10.1038/s41598-021-00169-x.

[93] B.E. Gee, A. Pearson, I. Buchanan-Perry, R.P. Simon, D.R. Archer, R. Meller, Whole Blood Transcriptome Analysis in Children with Sickle Cell Anemia, Front Genet 12 (2021) 737741. https://doi.org/10.3389/fgene.2021.737741.

[94] H. Frangoul, D. Altshuler, M.D. Cappellini, Y.-S. Chen, J. Domm, B.K. Eustace, J. Foell, J. de la Fuente, S. Grupp, R. Handgretinger, T.W. Ho, A. Kattamis, A. Kernytsky, J. Lekstrom-Himes, A.M. Li, F. Locatelli, M.Y. Mapara, M. de Montalembert, D. Rondelli, A. Sharma, S. Sheth, S. Soni, M.H. Steinberg, D. Wall, A. Yen, S. Corbacioglu, CRISPR-Cas9 Gene Editing for Sickle Cell Disease and β-Thalassemia, N Engl J Med 384 (2021) 252–260. https://doi.org/10.1056/NEJMoa2031054.

[95] S.N. Escobar Alvarez, E.R. Myers, Impact of a grant program to spur advances in sickle cell disease research, Blood Adv 5 (2021) 3855–3861. https://doi.org/10.1182/bloodadvances.2021005709.

[96] M.K. Cromer, J. Camarena, R.M. Martin, B.J. Lesch, C.A. Vakulskas, N.M. Bode, G. Kurgan, M.A. Collingwood, G.R. Rettig, M.A. Behlke, V.T. Lemgart, Y. Zhang, A. Goyal, F. Zhao, E. Ponce, W. Srifa, R.O. Bak, N. Uchida, R. Majeti, V.A. Sheehan, J.F. Tisdale, D.P. Dever, M.H. Porteus, Gene replacement of α-globin with β-globin restores hemoglobin balance in β-thalassemia-derived hematopoietic stem and progenitor cells, Nat Med 27 (2021) 677–687. https://doi.org/10.1038/s41591-021-01284-y.

[97] O. Chinedu, W.V. Tonassé, D.M. Albuquerque, I. de F. Domingos, A. da S. Araújo, M.A.C. Bezerra, M. de F. Sonati, M.N.N.D. Santos, Polymorphisms in the heme oxygenase-1 and bone morphogenetic protein receptor type 1b genes and estimated glomerular filtration rate in Brazilian sickle cell anemia patients, Hematol Transfus Cell Ther 43 (2021) 165–170. https://doi.org/10.1016/j.htct.2020.01.009.

[98] H. Chen, Z. Wang, S. Yu, X. Han, Y. Deng, F. Wang, Y. Chen, X. Liu, J. Zhou, J. Zhu, H. Yuan, 3,3',5-Triiodothyroacetic acid (TRIAC) induces embryonic ζ-globin expression via thyroid hormone receptor α, J Hematol Oncol 14 (2021) 99. https://doi.org/10.1186/s13045-021-01108-z.

[99] M. Brusson, A. Miccio, Genome editing approaches to β-hemoglobinopathies, Prog Mol Biol Transl Sci 182 (2021) 153–183. https://doi.org/10.1016/bs.pmbts.2021.01.025.

[100] J.V.G.F. Batista, G.S. Arcanjo, T.H.C. Batista, M.J. Sobreira, R.M. Santana, I.F. Domingos, B.L. Hatzlhofer, D.A. Falcão, D.A. Pereira-Martins, J.M. Oliveira, A.S. Araujo, L.P.M. Laranjeira, F.S. Medeiros, F.P. Albuquerque, D.M. Albuquerque, M.N. Santos, M.F. Hazin, A.C. Dos Anjos, F.F. Costa, A.S. Araujo, A.R. Lucena-Araujo, M.A. Bezerra, Influence of UGT1A1 promoter polymorphism, α-thalassemia and βs haplotype in bilirubin levels and cholelithiasis in a large sickle cell anemia cohort, Ann Hematol 100 (2021) 903–911. https://doi.org/10.1007/s00277-021-04422-1.

[101] X. Bao, X. Zhang, L. Wang, Z. Wang, J. Huang, Q. Zhang, Y. Ye, Y. Liu, D. Chen, Y. Zuo, Q. Liu, P. Xu, B. Huang, J. Fang, J. Lao, X. Feng, Y. Li, R. Kurita, Y. Nakamura, W. Yu, C. Ju, C. Huang, N. Mohandas, D. Li, C. Zhao, X. Xu, Epigenetic inactivation of ERF reactivates γ-globin expression in β-thalassemia, Am J Hum Genet 108 (2021) 709–721. https://doi.org/10.1016/j.ajhg.2021.03.005.

[102] N.-A. Aziz, W.-R.W. Taib, N.-K. Kharolazaman, I. Ismail, H.A.N. Al-Jamal, N.W.-A.W.A. Jamil, E. Esa, H. Ibrahim, Evidence of new intragenic HBB haplotypes model

for the prediction of beta-thalassemia in the Malaysian population, Sci Rep 11 (2021) 16772. https://doi.org/10.1038/s41598-021-96018-y.
[103] J. Zhan, M.J. Irudayam, Y. Nakamura, R. Kurita, A.W. Nienhuis, High level of fetal-globin reactivation by designed transcriptional activator-like effector, Blood Adv 4 (2020) 687–695. https://doi.org/10.1182/bloodadvances.2019000482.
[104] J. Zeng, Y. Wu, C. Ren, J. Bonanno, A.H. Shen, D. Shea, J.M. Gehrke, K. Clement, K. Luk, Q. Yao, R. Kim, S.A. Wolfe, J.P. Manis, L. Pinello, J.K. Joung, D.E. Bauer, Therapeutic base editing of human hematopoietic stem cells, Nat Med 26 (2020) 535–541. https://doi.org/10.1038/s41591-020-0790-y.
[105] D. Wei, Y. Yang, C.J. Ricketts, C.D. Vocke, M.W. Ball, C. Sourbier, D. Wangsa, D. Wangsa, R. Guha, X. Zhang, K. Wilson, L. Chen, P.S. Meltzer, T. Ried, C.J. Thomas, M.J. Merino, W.M. Linehan, Novel renal medullary carcinoma cell lines, UOK353 and UOK360, provide preclinical tools to identify new therapeutic treatments, Genes Chromosomes Cancer 59 (2020) 472–483. https://doi.org/10.1002/gcc.22847.
[106] X. Wang, J.Z. Xu, A. Conrey, L. Mendelsohn, D. Shriner, M. Pirooznia, S.L. Thein, Whole genome sequence-based haplotypes reveal a single origin of the 1393 bp HBB deletion, J Med Genet 57 (2020) 567–570. https://doi.org/10.1136/jmedgenet-2019-106698.
[107] L.D.C.R. de la Torre, F.J.P. Díaz, B.I. Cortés, V.M.R. López, J.Y.S. López, F.J.S. Anzaldo, M.T.M. Torres, K. Gonnet, C. Badens, N. Bonello-Palot, Three Mexican Families with β thalassemia intermedia with different molecular basis, Genet Mol Biol 42 (2020) e20190032. https://doi.org/10.1590/1678-4685-GMB-2019-0032.
[108] M. Silva, S. Vargas, A. Coelho, E. Ferreira, J. Mendonça, L. Vieira, R. Maia, A. Dias, T. Ferreira, A. Morais, I.M. Soares, J. Lavinha, R. Silva, P. Kjöllerström, P. Faustino, Biomarkers and genetic modulators of cerebral vasculopathy in sub-Saharan ancestry children with sickle cell anemia, Blood Cells Mol Dis 83 (2020) 102436. https://doi.org/10.1016/j.bcmd.2020.102436.
[109] E. Saller, J. Knijnenburg, C.L. Harteveld, F. Dutly, A Woman with Missing Hb A2 Due to a Novel (εγ)δβ0-Thalassemia and a Novel δ-Globin Variant Hb A2-Gebenstorf (HBD: c.209G>A), Hemoglobin 44 (2020) 214–217. https://doi.org/10.1080/03630269.2020.1779739.
[110] R.R. Sales, A.R. Belisário, G. Faria, F. Mendes, M.R. Luizon, M.B. Viana, Functional polymorphisms of BCL11A and HBS1L-MYB genes affect both fetal hemoglobin level and clinical outcomes in a cohort of children with sickle cell anemia, Ann Hematol 99 (2020) 1453–1463. https://doi.org/10.1007/s00277-020-04079-2.
[111] P. Ropero, F.A. González, J.M. Nieto, A. Villegas, J. Sevilla, G. Pérez, J.M. Alonso, V. Recasens, M. Abio, J.M. Vagace, R.J. Vanegas, B. González Fernández, R. Martínez, C>A substitution in NT 46 of the 3’ UTR region (the α complex protected region) of the alpha-1 globin gene: a non-deletional mutation or polymorphism?, J Clin Pathol 73 (2020) 14–16. https://doi.org/10.1136/jclinpath-2019-206004.
[112] M. Ponomarenko, E. Sharypova, I. Drachkova, I. Chadaeva, O. Arkova, O. Podkolodnaya, P. Ponomarenko, N. Kolchanov, L. Savinkova, Unannotated single nucleotide polymorphisms in the TATA box of erythropoiesis genes show in vitro positive involvements in cognitive and mental disorders, BMC Med Genet 21 (2020) 165. https://doi.org/10.1186/s12881-020-01106-x.
[113] D.C. Ortega, H. Cárdenas, G. Barreto, Joint selection for two malaria resistance mutations in a south-west Colombian population, Infect Genet Evol 80 (2020) 104188. https://doi.org/10.1016/j.meegid.2020.104188.
[114] L.D. Notarangelo, A. Agostini, M. Casale, P. Samperi, F. Arcioni, P. Gorello, S. Perrotta, N. Masera, A. Barone, E. Bertoni, E. Bonetti, R. Burnelli, T. Casini, G.C. Del

Vecchio, B. Filippini, F. Giona, P. Giordano, C. Gorio, E. Marchina, M. Nardi, A. Petrone, R. Colombatti, L. Sainati, G. Russo, HbS/β+ thalassemia: Really a mild disease? A National survey from the AIEOP Sickle Cell Disease Study Group with genotype-phenotype correlation, Eur J Haematol 104 (2020) 214–222. https://doi.org/10.1111/ejh.13362.
[115] R.A. Morgan, M.J. Unti, B. Aleshe, D. Brown, K.S. Osborne, C. Koziol, P.G. Ayoub, O.B. Smith, R. O'Brien, C. Tam, E. Miyahira, M. Ruiz, J.P. Quintos, S. Senadheera, R.P. Hollis, D.B. Kohn, Improved Titer and Gene Transfer by Lentiviral Vectors Using Novel, Small β-Globin Locus Control Region Elements, Mol Ther 28 (2020) 328–340. https://doi.org/10.1016/j.ymthe.2019.09.020.
[116] R.A. Morgan, F. Ma, M.J. Unti, D. Brown, P.G. Ayoub, C. Tam, L. Lathrop, B. Aleshe, R. Kurita, Y. Nakamura, S. Senadheera, R.L. Wong, R.P. Hollis, M. Pellegrini, D.B. Kohn, Creating New β-Globin-Expressing Lentiviral Vectors by High-Resolution Mapping of Locus Control Region Enhancer Sequences, Mol Ther Methods Clin Dev 17 (2020) 999–1013. https://doi.org/10.1016/j.omtm.2020.04.006.
[117] M.S.M. Khalil, A.T. Timbs, S.J. Henderson, A. Schuh, J.M. Old, Fifteen Cases of Hb J-Meerut: The Rare Association with Hb E and/or HBA1: c.-24C>G (or HBA2) Variants, Hemoglobin 44 (2020) 364–367. https://doi.org/10.1080/03630269.2020.1817755.
[118] P. Huang, S.A. Peslak, X. Lan, E. Khandros, J.A. Yano, M. Sharma, C.A. Keller, B. Giardine, K. Qin, O. Abdulmalik, R.C. Hardison, J. Shi, G.A. Blobel, The HRI-regulated transcription factor ATF4 activates BCL11A transcription to silence fetal hemoglobin expression, Blood 135 (2020) 2121–2132. https://doi.org/10.1182/blood.2020005301.
[119] F. Forouzesh Pour, K. Karimi, Z. Ghaderi, A. Tavakoli Koudehi, H. Najmabadi, Heterozygosity for the Novel HBA2: c.*91_*92delTA Polyadenylation Site Variant on the α2-Globin Gene Expanding the Genetic Spectrum of α-Thalassemia in Iran, Hemoglobin 44 (2020) 423–426. https://doi.org/10.1080/03630269.2020.1831529.
[120] C. Fong, Y. Mendoza, G. Barreto, Genetic variants in the G gamma-globin promoter modulate fetal hemoglobin expression in the Colombian population, Genet Mol Biol 43 (2020) e20190076. https://doi.org/10.1590/1678-4685-GMB-2019-0076.
[121] M. El-Ghamrawy, M.E. Yassa, A.M.S. Tousson, M.A. El-Hady, E. Mikhaeil, N.B. Mohamed, M.M. Khorshied, Association between BCL11A, HSB1L-MYB, and XmnI γG-158 (C/T) gene polymorphism and hemoglobin F level in Egyptian sickle cell disease patients, Ann Hematol 99 (2020) 2279–2288. https://doi.org/10.1007/s00277-020-04187-z.
[122] S. Demirci, J. Zeng, Y. Wu, N. Uchida, A.H. Shen, D. Pellin, J. Gamer, M. Yapundich, C. Drysdale, J. Bonanno, A.C. Bonifacino, A.E. Krouse, N.S. Linde, T. Engels, R.E. Donahue, J.J. Haro-Mora, A. Leonard, T. Nassehi, K. Luk, S.N. Porter, C.R. Lazzarotto, S.Q. Tsai, M.J. Weiss, S.M. Pruett-Miller, S.A. Wolfe, D.E. Bauer, J.F. Tisdale, BCL11A enhancer-edited hematopoietic stem cells persist in rhesus monkeys without toxicity, J Clin Invest 130 (2020) 6677–6687. https://doi.org/10.1172/JCI140189.
[123] A. Costa Neto, F. Santos, I. Ribeiro, V. Oliveira, M. Dezan, S. Kashima, D. Covas, A. Pereira, G. Fonseca, F. Moreira, J. Krieger, S. Gualandro, V. Rocha, A. Mendrone, C.L. Dinardo, FcγR2B B2.4 haplotype predicts increased risk of red blood cell alloimmunization in sickle cell disease patients, Transfusion 60 (2020) 1573–1578. https://doi.org/10.1111/trf.15832.
[124] L. Chaouch, H. Sellami, M. Kalai, I. Darragi, I. Boudrigua, D. Chaouachi, S. Abbes, S. Mnif, New Deletion at Promoter of HBG1 Gene in Sickle Cell Disease Patients With

High HbF Level, J Pediatr Hematol Oncol 42 (2020) 20–22. https://doi.org/10.1097/MPH.0000000000001626.
[125] Y. Barbanera, F. Arcioni, H. Lancioni, R. La Starza, I. Cardinali, C. Matteucci, V. Nofrini, A. Roetto, A. Piga, P. Grammatico, M. Caniglia, C. Mecucci, P. Gorello, Comprehensive analysis of mitochondrial and nuclear DNA variations in patients affected by hemoglobinopathies: A pilot study, PLoS One 15 (2020) e0240632. https://doi.org/10.1371/journal.pone.0240632.
[126] X. Bao, Y. Zuo, D. Chen, C. Zhao, DNA methylation patterns of β-globin cluster in β-thalassemia patients, Clin Epigenetics 12 (2020) 187. https://doi.org/10.1186/s13148-020-00987-2.
[127] V. Alexeeva, I.T. Aydin, C. Schaniel, A.W. Stranahan, S.L. D’Souza, J.J. Bieker, A human H1-HBB11-GFP reporter embryonic stem cell line (WAe001-A-2) generated using TALEN-based genome editing, Stem Cell Res 45 (2020) 101837. https://doi.org/10.1016/j.scr.2020.101837.
[128] F.-Z. Alaoui-Ismaili, A. Laghmich, N. Ghailani-Nourouti, A. Barakat, M. Bennani-Mechita, XmnI Polymorphism in Sickle Cell Disease in North Morocco, Hemoglobin 44 (2020) 190–194. https://doi.org/10.1080/03630269.2020.1772284.
[129] S. Yılmaz, The Spectrum of β-Thalassemia Mutations in Siirt Province, Southeastern Turkey, Hemoglobin 43 (2019) 174–181. https://doi.org/10.1080/03630269.2019.1647852.
[130] Y. Wu, J. Zeng, B.P. Roscoe, P. Liu, Q. Yao, C.R. Lazzarotto, K. Clement, M.A. Cole, K. Luk, C. Baricordi, A.H. Shen, C. Ren, E.B. Esrick, J.P. Manis, D.M. Dorfman, D.A. Williams, A. Biffi, C. Brugnara, L. Biasco, C. Brendel, L. Pinello, S.Q. Tsai, S.A. Wolfe, D.E. Bauer, Highly efficient therapeutic gene editing of human hematopoietic stem cells, Nat Med 25 (2019) 776–783. https://doi.org/10.1038/s41591-019-0401-y.
[131] A. Starlard-Davenport, A. Smith, L. Vu, B. Li, B.S. Pace, MIR29B mediates epigenetic mechanisms of HBG gene activation, Br J Haematol 186 (2019) 91–100. https://doi.org/10.1111/bjh.15870.
[132] B.N. Seamans, S.L. Pellechio, A.L. Capria, S.E. Agyingi, O.B. Morenikeji, O. Ojurongbe, B.N. Thomas, Genetic diversity of CD14, CD28, CTLA-4 and ICOS gene promoter polymorphism in African and American sickle cell disease, Hum Immunol 80 (2019) 930–936. https://doi.org/10.1016/j.humimm.2019.08.005.
[133] A.R. Oseghale, X. Zhu, B. Li, K.R. Peterson, A. Nudelman, A. Rephaeli, H. Xu, B.S. Pace, Conjugate prodrug AN-233 induces fetal hemoglobin expression in sickle erythroid progenitors and β-YAC transgenic mice, Blood Cells Mol Dis 79 (2019) 102345. https://doi.org/10.1016/j.bcmd.2019.102345.
[134] O.S. Olatunya, D.M. Albuquerque, G.O. Akanbi, O.S. Aduayi, A.B. Taiwo, O.A. Faboya, T.S. Kayode, D.P. Leonardo, A. Adekile, F.F. Costa, Uridine diphosphate glucuronosyl transferase 1A (UGT1A1) promoter polymorphism in young patients with sickle cell anaemia: report of the first cohort study from Nigeria, BMC Med Genet 20 (2019) 160. https://doi.org/10.1186/s12881-019-0899-3.
[135] N. Naderi, A. Namvar, N. Amani, N. Nasoohi, A. Bolhassani, Analysis of long non-coding RNA expression in hemophilia A patients, Hematology 24 (2019) 255–262. https://doi.org/10.1080/16078454.2018.1560934.
[136] A. Mishra, P. Sundaravadivel, S.K. Tripathi, R.K. Jha, J. Badrukhiya, N. Basak, I. Anerao, A. Sharma, A.E. Idowu, A. Mishra, S. Pandey, U. Kumar, S. Singh, S. Nizamuddin, N.C. Tupperwar, A.N. Jha, K. Thangaraj, Variations in macrophage migration inhibitory factor gene are not associated with visceral leishmaniasis in India, J Infect Public Health 12 (2019) 380–387. https://doi.org/10.1016/j.jiph.2018.12.011.

[137] J.-Y. Métais, P.A. Doerfler, T. Mayuranathan, D.E. Bauer, S.C. Fowler, M.M. Hsieh, V. Katta, S. Keriwala, C.R. Lazzarotto, K. Luk, M.D. Neel, S.S. Perry, S.T. Peters, S.N. Porter, B.Y. Ryu, A. Sharma, D. Shea, J.F. Tisdale, N. Uchida, S.A. Wolfe, K.J. Woodard, Y. Wu, Y. Yao, J. Zeng, S. Pruett-Miller, S.Q. Tsai, M.J. Weiss, Genome editing of HBG1 and HBG2 to induce fetal hemoglobin, Blood Adv 3 (2019) 3379–3392. https://doi.org/10.1182/bloodadvances.2019000820.
[138] G.E. Martyn, B. Wienert, R. Kurita, Y. Nakamura, K.G.R. Quinlan, M. Crossley, A natural regulatory mutation in the proximal promoter elevates fetal globin expression by creating a de novo GATA1 site, Blood 133 (2019) 852–856. https://doi.org/10.1182/blood-2018-07-863951.
[139] C.T. Lux, S. Pattabhi, M. Berger, C. Nourigat, D.A. Flowers, O. Negre, O. Humbert, J.G. Yang, C. Lee, K. Jacoby, I. Bernstein, H.-P. Kiem, A. Scharenberg, D.J. Rawlings, TALEN-Mediated Gene Editing of HBG in Human Hematopoietic Stem Cells Leads to Therapeutic Fetal Hemoglobin Induction, Mol Ther Methods Clin Dev 12 (2019) 175–183. https://doi.org/10.1016/j.omtm.2018.12.008.
[140] L. Liu, X. Zhu, A. Yu, C.M. Ward, B.S. Pace, δ-Aminolevulinate induces fetal hemoglobin expression by enhancing cellular heme biosynthesis, Exp Biol Med (Maywood) 244 (2019) 1220–1232. https://doi.org/10.1177/1535370219872995.
[141] S. Kodali, P. Ramachandran, I.N. Richard, J.-C. Wang, TTP-like syndrome associated with hemoglobin SC disease treated successfully with plasma and red cell exchange, Leuk Res Rep 12 (2019) 100179. https://doi.org/10.1016/j.lrr.2019.100179.
[142] M.A. Khosravi, M. Abbasalipour, J.-P. Concordet, J.V. Berg, S. Zeinali, A. Arashkia, K. Azadmanesh, T. Buch, M. Karimipoor, Targeted deletion of BCL11A gene by CRISPR-Cas9 system for fetal hemoglobin reactivation: A promising approach for gene therapy of beta thalassemia disease, Eur J Pharmacol 854 (2019) 398–405. https://doi.org/10.1016/j.ejphar.2019.04.042.
[143] M.G. Kapetanaki, O.T. Gbotosho, D. Sharma, F. Weidert, S.F. Ofori-Acquah, G.J. Kato, Free heme regulates placenta growth factor through NRF2-antioxidant response signaling, Free Radic Biol Med 143 (2019) 300–308. https://doi.org/10.1016/j.freeradbiomed.2019.08.009.
[144] F. Jiang, J. Li, J.-Y. Zhou, C. Liao, D.-Z. Li, Regulatory Single Nucleotide Polymorphism rs368698783 (G>A): a Genetic Modifier of Hb F Production Only under Erythropoietic Stress Characteristic for β-Globin Chain Deficiency?, Hemoglobin 43 (2019) 73–75. https://doi.org/10.1080/03630269.2019.1588130.
[145] W. Jia, S. Jia, P. Chen, Y. He, Construction and Analysis of a Long Non-Coding RNA (lncRNA)-Associated ceRNA Network in β-Thalassemia and Hereditary Persistence of Fetal Hemoglobin, Med Sci Monit 25 (2019) 7079–7086. https://doi.org/10.12659/MSM.915946.
[146] E.H. Jhun, N. Sadhu, X. Hu, Y. Yao, Y. He, D.J. Wilkie, R.E. Molokie, Z.J. Wang, Beta2-Adrenergic Receptor Polymorphisms and Haplotypes Associate With Chronic Pain in Sickle Cell Disease, Front Pharmacol 10 (2019) 84. https://doi.org/10.3389/fphar.2019.00084.
[147] A. Fakhr-Eldeen, E.A. Toraih, M.S. Fawzy, Long non-coding RNAs MALAT1, MIAT and ANRIL gene expression profiles in beta-thalassemia patients: a cross-sectional analysis, Hematology 24 (2019) 308–317. https://doi.org/10.1080/16078454.2019.1570616.
[148] E. Drakopoulou, M. Georgomanoli, C.W. Lederer, M. Kleanthous, C. Costa, O. Bernadin, F.-L. Cosset, E. Voskaridou, E. Verhoeyen, E. Papanikolaou, N.P. Anagnou, A Novel BaEVRless-Pseudotyped γ-Globin Lentiviral Vector Drives High and Stable

Fetal Hemoglobin Expression and Improves Thalassemic Erythropoiesis In Vitro, Hum Gene Ther 30 (2019) 601–617. https://doi.org/10.1089/hum.2018.022.
[149] G. Breveglieri, F. Salvatori, A. Finotti, L.C. Cosenza, C. Zuccato, N. Bianchi, L. Breda, S. Rivella, A. Bresciani, M. Bisbocci, M. Borgatti, R. Gambari, Development and characterization of cellular biosensors for HTS of erythroid differentiation inducers targeting the transcriptional activity of γ-globin and β-globin gene promoters, Anal Bioanal Chem 411 (2019) 7669–7680. https://doi.org/10.1007/s00216-019-01959-z.
[150] B. Bayramov, G. Aliyeva, C. Asadov, T. Mammadova, N. Karimova, K. Eynullazadeh, S. Gafarova, S. Akbarov, S. Farhadova, Z. Safarzadeh, M. Abbasov, A Novel Frameshift Mutation at Codon 2 (-T) (HBB: c.9delT) and First Report of Three New β-Globin Mutations From Azerbaijan, Hemoglobin 43 (2019) 280–282. https://doi.org/10.1080/03630269.2019.1657886.
[151] A. Azimi, P. Nejati, S. Tahmasebi, S. Alimoradi, R. Alibakhshi, Characterization of the IVS-II-821 (A>C) (HBB: c.316-30A>C) Mutation in a β-Thalassemia Phenotype in Iran, Hemoglobin 43 (2019) 23–26. https://doi.org/10.1080/03630269.2019.1592760.
[152] D.B. Amle, R.L. Patnayak, V. Verma, G.K. Singh, V. Jain, P.K. Khodiar, P.K. Patra, VEGF Promoter Region 18-bp Insertion-Deletion Polymorphism in Sickle Cell Disease Patients with Microalbuminuria: A Pilot Study, Indian J Hematol Blood Transfus 35 (2019) 278–283. https://doi.org/10.1007/s12288-018-1018-x.
[153] S. Abdulazeez, S. Sultana, N.B. Almandil, D. Almohazey, B.J. Bency, J.F. Borgio, The rs61742690 (S783N) single nucleotide polymorphism is a suitable target for disrupting BCL11A-mediated foetal-to-adult globin switching, PLoS One 14 (2019) e0212492. https://doi.org/10.1371/journal.pone.0212492.
[154] L.M. Williams, Z. Qi, K. Batai, S. Hooker, N.J. Hall, R.F. Machado, A. Chen, S. Campbell-Lee, Y. Guan, R. Kittles, N.A. Hanchard, A locus on chromosome 5 shows African ancestry-limited association with alloimmunization in sickle cell disease, Blood Adv 2 (2018) 3637–3647. https://doi.org/10.1182/bloodadvances.2018020594.
[155] L. Weber, V. Poletti, E. Magrin, C. Antoniani, S. Martin, C. Bayard, H. Sadek, T. Felix, V. Meneghini, M.N. Antoniou, W. El-Nemer, F. Mavilio, M. Cavazzana, I. Andre-Schmutz, A. Miccio, An Optimized Lentiviral Vector Efficiently Corrects the Human Sickle Cell Disease Phenotype, Mol Ther Methods Clin Dev 10 (2018) 268–280. https://doi.org/10.1016/j.omtm.2018.07.012.
[156] G.R. Serjeant, B.E. Serjeant, K.P. Mason, M. Happich, A.E. Kulozik, β-Thalassemia Mutations in Jamaica: Geographic Variation in Small Communities, Hemoglobin 42 (2018) 294–296. https://doi.org/10.1080/03630269.2018.1540354.
[157] L.M. Raffield, J.C. Ulirsch, R.P. Naik, S. Lessard, R.E. Handsaker, D. Jain, H.M. Kang, N. Pankratz, P.L. Auer, E.L. Bao, J.D. Smith, L.A. Lange, E.M. Lange, Y. Li, T.A. Thornton, B.A. Young, G.R. Abecasis, C.C. Laurie, D.A. Nickerson, S.A. McCarroll, A. Correa, J.G. Wilson, NHLBI Trans-Omics for Precision Medicine (TOPMed) Consortium, Hematology & Hemostasis, Diabetes, and Structural Variation TOPMed Working Groups, G. Lettre, V.G. Sankaran, A.P. Reiner, Common α-globin variants modify hematologic and other clinical phenotypes in sickle cell trait and disease, PLoS Genet 14 (2018) e1007293. https://doi.org/10.1371/journal.pgen.1007293.
[158] V. Poletti, F. Urbinati, S. Charrier, G. Corre, R.P. Hollis, B. Campo Fernandez, S. Martin, M. Rothe, A. Schambach, D.B. Kohn, F. Mavilio, Pre-clinical Development of a Lentiviral Vector Expressing the Anti-sickling βAS3 Globin for Gene Therapy for Sickle Cell Disease, Mol Ther Methods Clin Dev 11 (2018) 167–179. https://doi.org/10.1016/j.omtm.2018.10.014.

[159] T.A. Morrison, I. Wilcox, H.-Y. Luo, J.J. Farrell, R. Kurita, Y. Nakamura, G.J. Murphy, S. Cui, M.H. Steinberg, D.H.K. Chui, A long noncoding RNA from the HBS1L-MYB intergenic region on chr6q23 regulates human fetal hemoglobin expression, Blood Cells Mol Dis 69 (2018) 1–9. https://doi.org/10.1016/j.bcmd.2017.11.003.
[160] F. Moassas, A. Alabloog, H. Murad, Description of a Rare β-Globin Gene Mutation: -86 (C>G) (HBB: c.-136C>G) Observed in a Syrian Family, Hemoglobin 42 (2018) 203–205. https://doi.org/10.1080/03630269.2018.1500918.
[161] G.E. Martyn, B. Wienert, L. Yang, M. Shah, L.J. Norton, J. Burdach, R. Kurita, Y. Nakamura, R.C.M. Pearson, A.P.W. Funnell, K.G.R. Quinlan, M. Crossley, Natural regulatory mutations elevate the fetal globin gene via disruption of BCL11A or ZBTB7A binding, Nat Genet 50 (2018) 498–503. https://doi.org/10.1038/s41588-018-0085-0.
[162] N. Liu, V.V. Hargreaves, Q. Zhu, J.V. Kurland, J. Hong, W. Kim, F. Sher, C. Macias-Trevino, J.M. Rogers, R. Kurita, Y. Nakamura, G.-C. Yuan, D.E. Bauer, J. Xu, M.L. Bulyk, S.H. Orkin, Direct Promoter Repression by BCL11A Controls the Fetal to Adult Hemoglobin Switch, Cell 173 (2018) 430-442.e17. https://doi.org/10.1016/j.cell.2018.03.016.
[163] C. Li, N. Psatha, H. Wang, M. Singh, H.B. Samal, W. Zhang, A. Ehrhardt, Z. Izsvák, T. Papayannopoulou, A. Lieber, Integrating HDAd5/35++ Vectors as a New Platform for HSC Gene Therapy of Hemoglobinopathies, Mol Ther Methods Clin Dev 9 (2018) 142–152. https://doi.org/10.1016/j.omtm.2018.02.004.
[164] C. Li, N. Psatha, P. Sova, S. Gil, H. Wang, J. Kim, C. Kulkarni, C. Valensisi, R.D. Hawkins, G. Stamatoyannopoulos, A. Lieber, Reactivation of γ-globin in adult β-YAC mice after ex vivo and in vivo hematopoietic stem cell genome editing, Blood 131 (2018) 2915–2928. https://doi.org/10.1182/blood-2018-03-838540.
[165] S. He, Q. Qin, L. Lin, Y. Zuo, Q. Chen, H. Wei, C. Zheng, B. Chen, X. Qiu, First Identification of the 3.5 kb Deletion (NC_000011.10: g.5224302-5227791del3490bp) on the β-Globin Gene Cluster in a Chinese Family, Hemoglobin 42 (2018) 272–275. https://doi.org/10.1080/03630269.2018.1531018.
[166] B. Gudmundsdottir, K.O. Gudmundsson, K.D. Klarmann, S.K. Singh, L. Sun, S. Singh, Y. Du, V. Coppola, L. Stockwin, N. Nguyen, L. Tessarollo, L. Thorsteinsson, O.E. Sigurjonsson, S. Gudmundsson, T. Rafnar, J.F. Tisdale, J.R. Keller, POGZ Is Required for Silencing Mouse Embryonic β-like Hemoglobin and Human Fetal Hemoglobin Expression, Cell Rep 23 (2018) 3236–3248. https://doi.org/10.1016/j.celrep.2018.05.043.
[167] R.M. Grimholt, C.L. Harteveld, S.G.J. Arkesteijn, B. Fjeld, O. Klingenberg, Characterization of Two Deep Intronic Variants on the β-Globin Gene with Inconsistent Interpretations of Clinical Significance, Hemoglobin 42 (2018) 126–128. https://doi.org/10.1080/03630269.2018.1473255.
[168] S.S. Ganaie, A.Y. Chen, C. Huang, P. Xu, S. Kleiboeker, A. Du, J. Qiu, RNA Binding Protein RBM38 Regulates Expression of the 11-Kilodalton Protein of Parvovirus B19, Which Facilitates Viral DNA Replication, J Virol 92 (2018) e02050-17. https://doi.org/10.1128/JVI.02050-17.
[169] H. Galehdari, S.Z. Azarshin, M. Bijanzadeh, M. Shafiei, Polymorphism studies on microRNA targetome of thalassemia, Bioinformation 14 (2018) 252–258. https://doi.org/10.6026/97320630014252.
[170] S. David, P. Aguiar, L. Antunes, A. Dias, A. Morais, A. Sakuntabhai, J. Lavinha, Variants in the non-coding region of the TLR2 gene associated with infectious

subphenotypes in pediatric sickle cell anemia, Immunogenetics 70 (2018) 37–51. https://doi.org/10.1007/s00251-017-1013-7.

[171] F. Bernaudin, C. Arnaud, A. Kamdem, I. Hau, F. Lelong, R. Epaud, C. Pondarré, S. Pissard, Biological impact of α genes, β haplotypes, and G6PD activity in sickle cell anemia at baseline and with hydroxyurea, Blood Adv 2 (2018) 626–637. https://doi.org/10.1182/bloodadvances.2017014555.

[172] C. Ben Hamda, R. Sangeda, L. Mwita, A. Meintjes, S. Nkya, S. Panji, N. Mulder, L. Guizani-Tabbane, A. Benkahla, J. Makani, K. Ghedira, H3ABioNet Consortium, A common molecular signature of patients with sickle cell disease revealed by microarray meta-analysis and a genome-wide association study, PLoS One 13 (2018) e0199461. https://doi.org/10.1371/journal.pone.0199461.

[173] Z.A. Al-Ali, R.K. Fallatah, E.A. Aljaffer, E.R. Albukhari, N. Sadek Al-Ali, Z.T. Al-Ghannam, R. Sayeb Al-Atrash, A. Alsuliman, C. Vatte, ANTXR1 Intronic Variants Are Associated with Fetal Hemoglobin in the Arab-Indian Haplotype of Sickle Cell Disease, Acta Haematol 140 (2018) 55–59. https://doi.org/10.1159/000491688.

[174] R.A.-R. Afifi, D. Kamal, R.E. Sayed, S.M.M. Ekladious, G.H. Shaheen, S.M. Yousry, R.E. Hussein, CD209-336A/G promotor polymorphism and its clinical associations in sickle cell disease Egyptian Pediatric patients, Hematol Oncol Stem Cell Ther 11 (2018) 75–81. https://doi.org/10.1016/j.hemonc.2017.09.002.

[175] T.A. Adeyemo, O.O. Ojewunmi, I.A. Oyetunji, H. Rooks, D.C. Rees, A.O. Akinsulie, A.S. Akanmu, S.L. Thein, S. Menzel, A survey of genetic fetal-haemoglobin modifiers in Nigerian patients with sickle cell anaemia, PLoS One 13 (2018) e0197927. https://doi.org/10.1371/journal.pone.0197927.

[176] X. Zhu, T. Hu, M.H. Ho, Y. Wang, M. Yu, N. Patel, W. Pi, J.-H. Choi, H. Xu, V. Ganapathy, F. Kutlar, A. Kutlar, D. Tuan, Hydroxyurea differentially modulates activator and repressors of γ-globin gene in erythroblasts of responsive and non-responsive patients with sickle cell disease in correlation with Index of Hydroxyurea Responsiveness, Haematologica 102 (2017) 1995–2004. https://doi.org/10.3324/haematol.2017.175646.

[177] Y. Wang, Y. Wang, L. Ma, M. Nie, J. Ju, M. Liu, Y. Deng, B. Yao, T. Gui, X. Li, C. Guo, C. Ma, R. Tan, Q. Zhao, Heterochromatin Protein 1γ Is a Novel Epigenetic Repressor of Human Embryonic ϵ-Globin Gene Expression, J Biol Chem 292 (2017) 4811–4817. https://doi.org/10.1074/jbc.M116.768515.

[178] C.-W. Sun, L.-C. Wu, P.L. Knopick, D.S. Bradley, T. Townes, D.S. Terman, Sickle cells produce functional immune modulators and cytotoxics, Am J Hematol 92 (2017) 981–988. https://doi.org/10.1002/ajh.24836.

[179] P. Ropero, S. Erquiaga, B. Arrizabalaga, G. Pérez, S. de la Iglesia, M.J. Torrejón, C. Gil, C. Elena, M. Tenorio, J.M. Nieto, F. de la Fuente-Gonzalo, A. Villegas, F.-A. González Fernández, R. Martínez, Phenotype of mutations in the promoter region of the β-globin gene, J Clin Pathol 70 (2017) 874–878. https://doi.org/10.1136/jclinpath-2017-204378.

[180] G.D. Pule, V.J.N. Bitoungui, B.C. Chemegni, A.P. Kengne, A. Wonkam, SAR1a promoter polymorphisms are not associated with fetal hemoglobin in patients with sickle cell disease from Cameroon, BMC Res Notes 10 (2017) 183. https://doi.org/10.1186/s13104-017-2502-3.

[181] L.J. Norton, A.P.W. Funnell, J. Burdach, B. Wienert, R. Kurita, Y. Nakamura, S. Philipsen, R.C.M. Pearson, K.G.R. Quinlan, M. Crossley, KLF1 directly activates expression of the novel fetal globin repressor ZBTB7A/LRF in erythroid cells, Blood Adv 1 (2017) 685–692. https://doi.org/10.1182/bloodadvances.2016002303.

[182] S. Mettananda, C.A. Fisher, D. Hay, M. Badat, L. Quek, K. Clark, P. Hublitz, D. Downes, J. Kerry, M. Gosden, J. Telenius, J.A. Sloane-Stanley, P. Faustino, A. Coelho, J. Doondeea, B. Usukhbayar, P. Sopp, J.A. Sharpe, J.R. Hughes, P. Vyas, R.J. Gibbons, D.R. Higgs, Editing an α-globin enhancer in primary human hematopoietic stem cells as a treatment for β-thalassemia, Nat Commun 8 (2017) 424. https://doi.org/10.1038/s41467-017-00479-7.
[183] J. Ma, F. Liu, X. Du, D. Ma, L. Xiong, Changes in lncRNAs and related genes in β-thalassemia minor and β-thalassemia major, Front Med 11 (2017) 74–86. https://doi.org/10.1007/s11684-017-0503-1.
[184] C.N. Lwanira, F. Kironde, M. Kaddumukasa, G. Swedberg, Prevalence of polymorphisms in glucose-6-phosphate dehydrogenase, sickle haemoglobin and nitric oxide synthase genes and their relationship with incidence of uncomplicated malaria in Iganga, Uganda, Malar J 16 (2017) 322. https://doi.org/10.1186/s12936-017-1970-1.
[185] K. Leecharoenkiat, Y. Tanaka, Y. Harada, P. Chaichompoo, O. Sarakul, Y. Abe, D.R. Smith, S. Fucharoen, S. Svasti, T. Umemura, Plasma microRNA-451 as a novel hemolytic marker for β0-thalassemia/HbE disease, Mol Med Rep 15 (2017) 2495–2502. https://doi.org/10.3892/mmr.2017.6326.
[186] P. Joly, C. Renoux, P. Lacan, Y. Bertrand, G. Cannas, N. Garnier, D. Cuzzubbo, K. Kebaïli, C. Renard, A. Gauthier, V. Pialoux, C. Martin, M. Romana, P. Connes, UGT1A1 (TA)n genotype is not the major risk factor of cholelithiasis in sickle cell disease children, Eur J Haematol 98 (2017) 296–301. https://doi.org/10.1111/ejh.12838.
[187] J.A. Heit, S.M. Armasu, B.M. McCauley, I.J. Kullo, H. Sicotte, J. Pathak, C.G. Chute, O. Gottesman, E.P. Bottinger, J.C. Denny, D.M. Roden, R. Li, M.D. Ritchie, M. de Andrade, Identification of unique venous thromboembolism-susceptibility variants in African-Americans, Thromb Haemost 117 (2017) 758–768. https://doi.org/10.1160/TH16-08-0652.
[188] P. Hariharan, M. Sawant, M. Gorivale, R. Manchanda, R. Colah, K. Ghosh, A. Nadkarni, Synergistic effect of two β globin gene cluster mutations leading to the hereditary persistence of fetal hemoglobin (HPFH) phenotype, Mol Biol Rep 44 (2017) 413–417. https://doi.org/10.1007/s11033-017-4125-0.
[189] S. Ge, B. Yang, W. Yi, K. Huang, H. Liu, X. Huang, J. Chu, Z. Yang, [Analysis of clinical phenotype and genotype of unstable Hemoglobin Rush], Zhonghua Yi Xue Yi Chuan Xue Za Zhi 34 (2017) 15–20. https://doi.org/10.3760/cma.j.issn.1003-9406.2017.01.004.
[190] L.A. de Azevedo, J. Bonazzoni, S.C. Wagner, M.G. Farias, C.M. Bittar, L. Daudt, S.M. de Castro, Do Alpha Thalassemia, Fetal Hemoglobin, and the UGT1A1 Polymorphism have an Influence on Serum Bilirubin Levels and Cholelithiasis in Patients with Sickle Cell Disease?, Mol Diagn Ther 21 (2017) 437–442. https://doi.org/10.1007/s40291-017-0283-y.
[191] Y. Dai, T. Chen, H. Ijaz, E.H. Cho, M.H. Steinberg, SIRT1 activates the expression of fetal hemoglobin genes, Am J Hematol 92 (2017) 1177–1186. https://doi.org/10.1002/ajh.24879.
[192] C. Cyrus, C. Vatte, J.F. Borgio, A. Al-Rubaish, S. Chathoth, Z.A. Nasserullah, S.A. Jarrash, A. Sulaiman, H. Qutub, H. Alsaleem, A.J. Alzahrani, M.H. Steinberg, A.K.A. Ali, Existence of HbF Enhancer Haplotypes at HBS1L-MYB Intergenic Region in Transfusion-Dependent Saudi β-Thalassemia Patients, Biomed Res Int 2017 (2017) 1972429. https://doi.org/10.1155/2017/1972429.
[193] D. Chen, Y. Zuo, X. Zhang, Y. Ye, X. Bao, H. Huang, W. Tepakhan, L. Wang, J. Ju, G. Chen, M. Zheng, D. Liu, S. Huang, L. Zong, C. Li, Y. Chen, C. Zheng, L. Shi, Q.

Zhao, Q. Wu, S. Fucharoen, C. Zhao, X. Xu, A Genetic Variant Ameliorates β-Thalassemia Severity by Epigenetic-Mediated Elevation of Human Fetal Hemoglobin Expression, Am J Hum Genet 101 (2017) 130–138. https://doi.org/10.1016/j.ajhg.2017.05.012.
[194] K.-H. Chang, S.E. Smith, T. Sullivan, K. Chen, Q. Zhou, J.A. West, M. Liu, Y. Liu, B.F. Vieira, C. Sun, V.P. Hong, M. Zhang, X. Yang, A. Reik, F.D. Urnov, E.J. Rebar, M.C. Holmes, O. Danos, H. Jiang, S. Tan, Long-Term Engraftment and Fetal Globin Induction upon BCL11A Gene Editing in Bone-Marrow-Derived CD34+ Hematopoietic Stem and Progenitor Cells, Mol Ther Methods Clin Dev 4 (2017) 137–148. https://doi.org/10.1016/j.omtm.2016.12.009.
[195] G.C.S. Carrocini, L.P.R. Venancio, V.L.R. Pessoa, C.L.C. Lobo, C.R. Bonini-Domingos, Mutational Profile of Homozygous β-Thalassemia in Rio de Janeiro, Brazil, Hemoglobin 41 (2017) 12–15. https://doi.org/10.1080/03630269.2017.1289958.
[196] V. Barresi, S. Lionti, L. Valori, G. Gallina, M. Caffo, S. Rossi, Dual-Genotype Diffuse Low-Grade Glioma: Is It Really Time to Abandon Oligoastrocytoma As a Distinct Entity?, J Neuropathol Exp Neurol 76 (2017) 342–346. https://doi.org/10.1093/jnen/nlx024.
[197] M. Zachariah, S. Al Zadjali, W. Bashir, R. Al Ambusaidi, R. Misquith, Y. Wali, A. Pathare, Impact of Mannose-Binding Protein Gene Polymorphisms in Omani Sickle Cell Disease Patients, Mediterr J Hematol Infect Dis 8 (2016) e2016013. https://doi.org/10.4084/MJHID.2016.013.
[198] M.-Y. Wu, D.-Z. Li, First Detection of the -27 (A > G) (HBB: c.-77A > G) Mutation of the β-Globin Gene in a Chinese Family, Hemoglobin 40 (2016) 59–60. https://doi.org/10.3109/03630269.2015.1100118.
[199] M. Vinciguerra, C. Passarello, F. Cassarà, F. Leto, M. Cannata, G. Calvaruso, R. Di Maggio, D. Renda, A. Maggio, A. Giambona, Co-heredity of silent CAP + 1570 T>C (HBB:c*96T>C) defect and severe β-thal mutation: a cause of mild β-thalassemia intermedia, Int J Lab Hematol 38 (2016) 17–26. https://doi.org/10.1111/ijlh.12433.
[200] E.A. Traxler, Y. Yao, Y.-D. Wang, K.J. Woodard, R. Kurita, Y. Nakamura, J.R. Hughes, R.C. Hardison, G.A. Blobel, C. Li, M.J. Weiss, A genome-editing strategy to treat β-hemoglobinopathies that recapitulates a mutation associated with a benign genetic condition, Nat Med 22 (2016) 987–990. https://doi.org/10.1038/nm.4170.
[201] A.K. Srivastava, Y. Wang, R. Huang, C. Skinner, T. Thompson, L. Pollard, T. Wood, F. Luo, R. Stevenson, R. Polimanti, J. Gelernter, X. Lin, I.Y. Lim, Y. Wu, A.L. Teh, L. Chen, I.M. Aris, S.E. Soh, M.T. Tint, J.L. MacIsaac, F. Yap, K. Kwek, S.M. Saw, M.S. Kobor, M.J. Meaney, K.M. Godfrey, Y.S. Chong, J.D. Holbrook, Y.S. Lee, P.D. Gluckman, N. Karnani, GUSTO study group, A. Kapoor, D. Lee, A. Chakravarti, C. Maercker, F. Graf, M. Boutros, G. Stamoulis, F. Santoni, P. Makrythanasis, A. Letourneau, M. Guipponi, N. Panousis, M. Garieri, P. Ribaux, E. Falconnet, C. Borel, S.E. Antonarakis, S. Kumar, J. Curran, J. Blangero, S. Chatterjee, A. Kapoor, J. Akiyama, D. Auer, C. Berrios, L. Pennacchio, A. Chakravarti, T.R. Donti, G. Cappuccio, M. Miller, P. Atwal, A. Kennedy, A. Cardon, C. Bacino, L. Emrick, J. Hertecant, F. Baumer, B. Porter, M. Bainbridge, P. Bonnen, B. Graham, R. Sutton, Q. Sun, S. Elsea, Z. Hu, P. Wang, Y. Zhu, J. Zhao, M. Xiong, D.A. Bennett, A. Hidalgo-Miranda, S. Romero-Cordoba, S. Rodriguez-Cuevas, R. Rebollar-Vega, E. Tagliabue, M. Iorio, E. D'Ippolito, S. Baroni, B. Kaczkowski, Y. Tanaka, H. Kawaji, A. Sandelin, R. Andersson, M. Itoh, T. Lassmann, The FANTOM5 Consortium, Y. Hayashizaki, P. Carninci, A.R.R. Forrest, C.A. Semple, E.A. Rosenthal, B. Shirts, L. Amendola, C. Gallego, M. Horike-Pyne, A. Burt, P. Robertson, P. Beyers, C. Nefcy, D. Veenstra, F.

Hisama, R. Bennett, M. Dorschner, D. Nickerson, J. Smith, K. Patterson, D. Crosslin, R. Nassir, N. Zubair, T. Harrison, U. Peters, G. Jarvik, NHLBI GO Exome Sequencing Project, F. Menghi, K. Inaki, X. Woo, P. Kumar, K. Grzeda, A. Malhotra, H. Kim, D. Ucar, P. Shreckengast, K. Karuturi, J. Keck, J. Chuang, E.T. Liu, B. Ji, A. Tyler, G. Ananda, G. Carter, H. Nikbakht, M. Montagne, M. Zeinieh, A. Harutyunyan, M. Mcconechy, N. Jabado, P. Lavigne, J. Majewski, J.B. Goldstein, M. Overman, G. Varadhachary, R. Shroff, R. Wolff, M. Javle, A. Futreal, D. Fogelman, L. Bravo, W. Fajardo, H. Gomez, C. Castaneda, C. Rolfo, J.A. Pinto, K.C. Akdemir, L. Chin, A. Futreal, ICGC PCAWG Structural Alterations Group, S. Patterson, C. Statz, S. Mockus, S.N. Nikolaev, X.I. Bonilla, L. Parmentier, B. King, F. Bezrukov, G. Kaya, V. Zoete, V. Seplyarskiy, H. Sharpe, T. McKee, A. Letourneau, P. Ribaux, K. Popadin, N. Basset-Seguin, R.B. Chaabene, F. Santoni, M. Andrianova, M. Guipponi, M. Garieri, C. Verdan, K. Grosdemange, O. Sumara, M. Eilers, I. Aifantis, O. Michielin, F. de Sauvage, S. Antonarakis, S. Likhitrattanapisal, S. Lincoln, A. Kurian, A. Desmond, S. Yang, Y. Kobayashi, J. Ford, L. Ellisen, T.L. Peters, K.R. Alvarez, E.F. Hollingsworth, D.H. Lopez-Terrada, A. Hastie, Z. Dzakula, A.W. Pang, E.T. Lam, T. Anantharaman, M. Saghbini, H. Cao, BioNano Genomics, C. Gonzaga-Jauregui, L. Ma, A. King, E.B. Rosenzweig, U. Krishnan, J.G. Reid, J.D. Overton, F. Dewey, W.K. Chung, K. Small, A. DeLuca, F. Cremers, R.A. Lewis, V. Puech, B. Bakall, R. Silva-Garcia, K. Rohrschneider, M. Leys, F.S. Shaya, E. Stone, N.L. Sobreira, F. Schiettecatte, H. Ling, E. Pugh, D. Witmer, K. Hetrick, P. Zhang, K. Doheny, D. Valle, A. Hamosh, S.N. Jhangiani, Z.C. Akdemir, M.N. Bainbridge, W. Charng, W. Wiszniewski, T. Gambin, E. Karaca, Y. Bayram, M.K. Eldomery, J. Posey, H. Doddapaneni, J. Hu, V.R. Sutton, D.M. Muzny, E.A. Boerwinkle, D. Valle, J.R. Lupski, R.A. Gibbs, S. Shekar, W. Salerno, A. English, A. Mangubat, J. Bruestle, A. Thorogood, B.M. Knoppers, Global Alliance for Genomics and Health - Regulatory and Ethics Working Group, H. Takahashi, K.R. Nitta, A. Kozhuharova, A.M. Suzuki, H. Sharma, D. Cotella, C. Santoro, S. Zucchelli, S. Gustincich, P. Carninci, J.J. Mulvihill, G. Baynam, W. Gahl, S.C. Groft, K. Kosaki, P. Lasko, B. Melegh, D. Taruscio, R. Ghosh, S. Plon, S. Scherer, X. Qin, R. Sanghvi, K. Walker, T. Chiang, D. Muzny, L. Wang, J. Black, E. Boerwinkle, R. Weinshilboum, R. Gibbs, T. Karpinets, T. Calderone, K. Wani, X. Yu, C. Creasy, C. Haymaker, M. Forget, V. Nanda, J. Roszik, J. Wargo, L. Haydu, X. Song, A. Lazar, J. Gershenwald, M. Davies, C. Bernatchez, J. Zhang, A. Futreal, S. Woodman, E.J. Chesler, T. Reynolds, J.A. Bubier, C. Phillips, M.A. Langston, E.J. Baker, M. Xiong, L. Ma, N. Lin, C. Amos, N. Lin, P. Wang, Y. Zhu, J. Zhao, V. Calhoun, M. Xiong, O. Dobretsberger, M. Egger, F. Leimgruber, S. Sadedin, A. Oshlack, Melbourne Genomics Health Alliance, V. a. A. Antonio, N. Ono, Clark Kendrick C. Go, Z. Ahmed, M. Bolisetty, S. Zeeshan, E. Anguiano, D. Ucar, A. Sarkar, M.R. Nandineni, C. Zeng, J. Shao, H. Cao, A. Hastie, A.W. Pang, E.T. Lam, T. Liang, K. Pham, M. Saghbini, Z. Dzakula, Y. Chee-Wei, L. Dongsheng, W. Lai-Ping, D. Lian, R.O.T. Hee, Y. Yunus, F. Aghakhanian, S.S. Mokhtar, C.V. Lok-Yung, J. Bhak, M. Phipps, X. Shuhua, T. Yik-Ying, V. Kumar, H. Boon-Peng, I. Campbell, M.-A. Young, P. James, Lifepool, M. Rain, G. Mohammad, R. Kukreti, Q. Pasha, A.R. Akilzhanova, C. Guelly, Z. Abilova, S. Rakhimova, A. Akhmetova, U. Kairov, S. Trajanoski, Z. Zhumadilov, M. Bekbossynova, C. Schumacher, S. Sandhu, T. Harkins, V. Makarov, H. Doddapaneni, R. Glenn, Z. Momin, B. Dilrukshi, H. Chao, Q. Meng, B. Gudenkauf, R. Kshitij, J. Jayaseelan, C. Nessner, S. Lee, K. Blankenberg, L. Lewis, J. Hu, Y. Han, H. Dinh, S. Jireh, K. Walker, E. Boerwinkle, D. Muzny, R. Gibbs, J. Hu, K. Walker, C. Buhay, X. Liu, Q. Wang, R. Sanghvi, H. Doddapaneni, Y. Ding, N. Veeraraghavan, Y. Yang, E.

Boerwinkle, A.L. Beaudet, C.M. Eng, D.M. Muzny, R.A. Gibbs, K.C.C. Worley, Y. Liu, D.S.T. Hughes, S.C. Murali, R.A. Harris, A.C. English, X. Qin, O.A. Hampton, P. Larsen, C. Beck, Y. Han, M. Wang, H. Doddapaneni, C.L. Kovar, W.J. Salerno, A. Yoder, S. Richards, J. Rogers, J.R. Lupski, D.M. Muzny, R.A. Gibbs, Q. Meng, M. Bainbridge, M. Wang, H. Doddapaneni, Y. Han, D. Muzny, R. Gibbs, R.A. Harris, M. Raveenedran, C. Xue, M. Dahdouli, L. Cox, G. Fan, B. Ferguson, J. Hovarth, Z. Johnson, S. Kanthaswamy, M. Kubisch, M. Platt, D. Smith, E. Vallender, R. Wiseman, X. Liu, J. Below, D. Muzny, R. Gibbs, F. Yu, J. Rogers, J. Lin, Y. Zhang, Z. Ouyang, A. Moore, Z. Wang, J. Hofmann, M. Purdue, R. Stolzenberg-Solomon, S. Weinstein, D. Albanes, C.S. Liu, W.L. Cheng, T.T. Lin, Q. Lan, N. Rothman, S. Berndt, E.S. Chen, H. Bahrami, A. Khoshzaban, S.H. Keshal, H. Bahrami, A. Khoshzaban, S.H. Keshal, K.K.R. Alharbi, M. Zhalbinova, A. Akilzhanova, S. Rakhimova, M. Bekbosynova, S. Myrzakhmetova, M. Matar, N. Mili, R. Molinari, Y. Ma, S. Guerrier, N. Elhawary, M. Tayeb, N. Bogari, N. Qotb, S.A. McClymont, P.W. Hook, L.A. Goff, A. McCallion, Y. Kong, J.R. Charette, W.L. Hicks, J.K. Naggert, L. Zhao, P.M. Nishina, B.M. Edrees, M. Athar, F.A. Al-Allaf, M.M. Taher, W. Khan, A. Bouazzaoui, N.A. Harbi, R. Safar, H. Al-Edressi, A. Anazi, N. Altayeb, M.A. Ahmed, K. Alansary, Z. Abduljaleel, A. Kratz, P. Beguin, S. Poulain, M. Kaneko, C. Takahiko, A. Matsunaga, S. Kato, A.M. Suzuki, N. Bertin, T. Lassmann, R. Vigot, P. Carninci, C. Plessy, T. Launey, D. Graur, D. Lee, A. Kapoor, A. Chakravarti, J. Friis-Nielsen, J.M. Izarzugaza, S. Brunak, A. Chakraborty, J. Basak, A. Mukhopadhyay, B.S. Soibam, D. Das, N. Biswas, S. Das, S. Sarkar, A. Maitra, C. Panda, P. Majumder, H. Morsy, A. Gaballah, M. Samir, M. Shamseya, H. Mahrous, A. Ghazal, W. Arafat, M. Hashish, J.J. Gruber, N. Jaeger, M. Snyder, K. Patel, S. Bowman, T. Davis, D. Kraushaar, A. Emerman, S. Russello, N. Henig, C. Hendrickson, K. Zhang, M. Rodriguez-Dorantes, C.D. Cruz-Hernandez, C.D.P. Garcia-Tobilla, S. Solorzano-Rosales, N. Jäger, J. Chen, R. Haile, M. Hitchins, J.D. Brooks, M. Snyder, S. Jiménez-Morales, M. Ramírez, J. Nuñez, V. Bekker, Y. Leal, E. Jiménez, A. Medina, A. Hidalgo, J. Mejía, V. Halytskiy, J. Naggert, G.B. Collin, K. DeMauro, R. Hanusek, P.M. Nishina, K. Belhassa, K. Belhassan, L. Bouguenouch, I. Samri, H. Sayel, F.Z. moufid, I. El Bouchikhi, S. Trhanint, H. Hamdaoui, I. Elotmani, I. Khtiri, O. Kettani, L. Quibibo, M. Ahagoud, M. Abbassi, K. Ouldim, A.V. Marusin, A.N. Kornetov, M. Swarovskaya, K. Vagaiceva, V. Stepanov, E.M.C. De La Paz, R. Sy, J. Nevado, P. Reganit, L. Santos, J.D. Magno, F.E. Punzalan, D. Ona, E. Llanes, R.L. Santos-Cortes, R. Tiongco, J. Aherrera, L. Abrahan, P. Pagauitan-Alan, The Philippine Cardiogenomics Study Group, K.H. Morelli, J.S. Domire, N. Pyne, S. Harper, R. Burgess, M. Zhalbinova, A. Akilzhanova, S. Rakhimova, M. Bekbosynova, S. Myrzakhmetova, M.A. Gari, A. Dallol, H. Alsehli, A. Gari, M. Gari, A. Abuzenadah, M. Thomas, M. Sukhai, S. Garg, M. Misyura, T. Zhang, A. Schuh, T. Stockley, S. Kamel-Reid, S. Sherry, C. Xiao, D. Slotta, K. Rodarmer, M. Feolo, M. Kimelman, G. Godynskiy, C. O'Sullivan, E. Yaschenko, C. Xiao, E. Yaschenko, S. Sherry, C. Rangel-Escareño, H. Rueda-Zarate, I.A. Tayubi, R. Mohammed, on behalf of 1, I. Ahmed, T. Ahmed, S. Seth, S. Amin, X. Song, X. Mao, H. Sun, R.G. Verhaak, A. Futreal, J. Zhang, S.J. Whiite, T. Chiang, A. English, J. Farek, Z. Kahn, W. Salerno, N. Veeraraghavan, E. Boerwinkle, R. Gibbs, T. Kasukawa, M. Lizio, J. Harshbarger, S. Hisashi, J. Severin, A. Imad, S. Sahin, T.C. Freeman, K. Baillie, A. Sandelin, P. Carninci, A.R.R. Forrest, H. Kawaji, The FANTOM Consortium, W. Salerno, A. English, S.N. Shekar, A. Mangubat, J. Bruestle, E. Boerwinkle, R.A. Gibbs, A.H. Salem, M. Ali, A. Ibrahim, M. Ibrahim, H.A. Barrera, L. Garza, J.A. Torres, V. Barajas, A. Ulloa-Aguirre, D. Kershenobich, S. Mortaji, P. Guizar, E. Loera, K. Moreno, A. De León, D. Monsiváis, J. Gómez, R.

Cardiel, J.C. Fernandez-Lopez, V. Bonifaz-Peña, C. Rangel-Escareño, A. Hidalgo-Miranda, A.V. Contreras, L. Polfus, CHARGE and NHLBI Exome Sequence Project Working Groups, X. Wang, V. Philip, G. Carter, A.A. Abuzenadah, M. Gari, R. Turki, A. Dallol, A. Uyar, A. Kaygun, S. Zaman, E. Marquez, J. George, D. Ucar, C.L. Hendrickson, A. Emerman, D. Kraushaar, S. Bowman, N. Henig, T. Davis, S. Russello, K. Patel, D.B. Starr, M. Baird, B. Kirkpatrick, K. Sheets, R. Nitsche, L. Prieto-Lafuente, M. Landrum, J. Lee, W. Rubinstein, D. Maglott, P.K.R. Thavanati, A.E. de Dios, R.E.N. Hernandez, M.E.A. Aldrate, M.R.R. Mejia, K.R.R. Kanala, Z. Abduljaleel, W. Khan, F.A. Al-Allaf, M. Athar, M.M. Taher, N. Shahzad, A. Bouazzaoui, E. Huber, A. Dan, F.A. Al-Allaf, W. Herr, G. Sprotte, J. Köstler, A. Hiergeist, A. Gessner, R. Andreesen, E. Holler, F. Al-Allaf, A. Alashwal, Z. Abduljaleel, M. Taher, A. Bouazzaoui, H. Abalkhail, A. Al-Allaf, R. Bamardadh, M. Athar, O. Filiptsova, M. Kobets, Y. Kobets, I. Burlaka, I. Timoshyna, O. Filiptsova, M.N. Kobets, Y. Kobets, I. Burlaka, I. Timoshyna, O. Filiptsova, M.N. Kobets, Y. Kobets, I. Burlaka, I. Timoshyna, F.A. Al-allaf, M.T. Mohiuddin, A. Zainularifeen, A. Mohammed, H. Abalkhail, T. Owaidah, A. Bouazzaoui, Human genome meeting 2016 : Houston, TX, USA. 28 February - 2 March 2016, Hum Genomics 10 Suppl 1 (2016) 12. https://doi.org/10.1186/s40246-016-0063-5.

[202] C. Simonnet, N. Elanga, P. Joly, T. Vaz, M. Nacher, Genetic modulators of sickle cell disease in French Guiana: Markers of the slave trade, Am J Hum Biol 28 (2016) 811–816. https://doi.org/10.1002/ajhb.22871.

[203] M. Silva, S. Vargas, A. Coelho, A. Dias, T. Ferreira, A. Morais, R. Maia, P. Kjöllerström, J. Lavinha, P. Faustino, Hemorheological alterations in sickle cell anemia and their clinical consequences - The role of genetic modulators, Clin Hemorheol Microcirc 64 (2016) 859–866. https://doi.org/10.3233/CH-168048.

[204] J.W. Shay, N. Homma, R. Zhou, M.I. Naseer, A.G. Chaudhary, M. Al-Qahtani, N. Hirokawa, M. Goudarzi, A.J. Fornace, S. Baeesa, D. Hussain, M. Bangash, F. Alghamdi, H.-J. Schulten, A. Carracedo, I. Khan, H. Qashqari, N. Madkhali, M. Saka, K.S. Saini, A. Jamal, J. Al-Maghrabi, A. Abuzenadah, A. Chaudhary, M. Al Qahtani, G. Damanhouri, H. Alkhatabi, A. Goodeve, L. Crookes, N. Niksic, N. Beauchamp, A.M. Abuzenadah, J. Vaught, B. Budowle, M. Assidi, A. Buhmeida, J. Al-Maghrabi, A. Buhmeida, M. Assidi, L. Merdad, S. Kumar, S. Miura, K. Gomez, A. Carracedo, M. Rasool, A. Rebai, S. Karim, H.F.N. Eldin, H. Abusamra, E.M. Alhathli, N. Salem, M.H. Al-Qahtani, S. Kumar, H. Faheem, A. Agarwa, E. Nieschlag, J. Wistuba, O.S. Damm, M.A. Beg, T.A. Abdel-Meguid, H.A. Mosli, O.S. Bajouh, A.M. Abuzenadah, M.H. Al-Qahtani, S. Coskun, M. Abu-Elmagd, A. Buhmeida, A. Dallol, J. Al-Maghrabi, S. Hakamy, W. Al-Qahtani, A. Al-Harbi, S. Hussain, M. Assidi, M. Al-Qahtani, A. Abuzenadah, B. Ozkosem, R. DuBois, S.S. Messaoudi, M.T. Dandana, T. Mahjoub, W.Y. Almawi, S. Abdalla, M.N. Al-Aama, A. Elzawahry, T. Takahashi, S. Mimaki, E. Furukawa, R. Nakatsuka, I. Kurosaka, T. Nishigaki, H. Nakamura, S. Serada, T. Naka, S. Hirota, T. Shibata, K. Tsuchihara, T. Nishida, M. Kato, S. Mehmood, N.M. Ashraf, A. Asif, M. Bilal, M.S. Mehmood, A. Hussain, Q.M.S. Jamal, M.U. Siddiqui, M.A. Alzohairy, M.A. Al Karaawi, T. Nedjadi, J. Al-Maghrabi, M. Assidi, H. Al-Khattabi, A. Al-Ammari, A. Al-Sayyad, A. Buhmeida, M. Al-Qahtani, H. Zitouni, N. Raguema, M.B. Ali, W. Malah, R. Lfalah, W. Almawi, T. Mahjoub, M. Elanbari, A. Ptitsyn, S. Mahjoub, R. El Ghali, B. Achour, N.B. Amor, M. Assidi, B. N’siri, H. Morjani, T. Nedjadi, A. Al-Ammari, A. Al-Sayyad, N. Salem, E. Azhar, J. Al-Maghrabi, V. Chayeb, M. Dendena, H. Zitouni, K. Zouari-Limayem, T. Mahjoub, B. Refaat, A.M. Ashshi, S.A. Batwa, H. Ramadan, A. Awad, A. Ateya, A.G.A. El-Shemi, A. Ashshi, M. Basalamah, Y. Na, C.-O. Yun, A.G.A. El-Shemi, A. Ashshi, M.

Basalamah, Y. Na, C.-O. Yun, A.G. El-Shemi, B. Refaat, O. Kensara, A. Abdelfattah, B.I. Dheeb, M.M.F. Al-Halbosiy, R.K. Al lihabi, B.M. Khashman, D. Laiche, C. Adeel, N. Taoufik, H. Al-Afghani, M. Łastowska, H.H. Al-Balool, H. Sheth, E. Mercer, J.M. Coxhead, C.P.F. Redfern, H. Peters, A.D. Burt, M. Santibanez-Koref, C.M. Bacon, L. Chesler, A.G. Rust, D.J. Adams, D. Williamson, S.C. Clifford, M.S. Jackson, M. Singh, M.S. Mansuri, S.D. Jadeja, H. Patel, Y.S. Marfatia, R. Begum, A.M. Mohamed, A.K. Kamel, N.A. Helmy, S.A. Hammad, H.F. Kayed, M.I. Shehab, A. El Gerzawy, M.M. Ead, O.M. Ead, M. Mekkawy, I. Mazen, M. El-Ruby, S.M.A. Shahid, Q.M.S. Jamal, J.M. Arif, M. Lohani, M. Imen, C. Leila, O. Houyem, D. Kais, C.D.M. Fethi, B. Mohamed, A. Salem, A. Faggad, A.T. Gebreslasie, H.Y. Zaki, B.E. Abdalla, M.S. AlShammari, R. Al-Ali, N. Al-Balawi, M. Al-Enazi, A. Al-Muraikhi, F. Busaleh, A. Al-Sahwan, F. Borgio, A. Sayyed, A. Al-Ali, S. Acharya, M.S. Zaki, H.T. El-Bassyouni, M.I. Shehab, M.F. Elshal, K. M., A.M. Aldahlawi, O. Saadah, J.P. McCoy, A.E. El-Tarras, N.S. Awad, A.A. Alharthi, M.M.M. Ibrahim, H.S. Alsehli, A. Dallol, A.M. Gari, M.M. Abbas, R.A. Kadam, M.M. Gari, M.H. Alkaff, A.M. Abuzenadah, M.A. Gari, H. Abusamra, S. Karim, H.F.N. eldin, E.M. Alhathli, N. Salem, S. Kumar, M.H. Al-Qahtani, F.A. Moradi, O.M. Rashidi, Z.A. Awan, I.H. Kaya, O. Al-Harazi, D. Colak, N.A. Alkousi, T. Athanasopoulos, A.O. Bahmaid, E.A. Alhwait, M.A. Gari, H.S. Alsehli, M.M. Abbas, M.H. Alkaf, R. Kadam, A. Dallol, G. Kalamegam, H.F.N. Eldin, S. Karim, H. Abusamra, E. Alhathli, N. Salem, M.H. Al-Qahtani, S. Kumar, S.N. Alsayed, F.H. Aljohani, S.M. Habeeb, R.A. Almashali, S. Basit, S.M. Ahmed, R. Sharma, A. Agarwal, D. Durairajanayagam, L. Samanta, M. Abu-Elmagd, A.M. Abuzenadah, E.S. Sabanegh, M. Assidi, M. Al-Qahtani, A. Agarwal, R. Sharma, L. Samanta, D. Durairajanayagam, M. Assidi, M. Abu-Elmagd, M. Al-Qahtani, A.M. Abuzenadah, E.S. Sabanegh, L. Samanta, A. Agarwal, R. Sharma, Z. Cui, M. Assidi, A.M. Abuzenadah, M. Abu-Elmagd, M. Al-Qahtani, A.A. Alboogmi, N.A. Alansari, M.M. Al-Quaiti, F.T. Ashgan, A. Bandah, H.S. Jamal, A. Rozi, Z. Mirza, A.M. Abuzenadah, S. Karim, M.H. Al-Qahtani, S. Karim, H.-J. Schulten, A.J. Al Sayyad, H.M.A. Farsi, J.A. Al-Maghrabi, Z. Mirza, R. Alotibi, A. Al-Ahmadi, N.A. Alansari, A.A. Albogmi, M.M. Al-Quaiti, F.T. Ashgan, A. Bandah, M.H. Al-Qahtani, R.A. Ebiya, S.M. Darwish, M.M. Montaser, H. Abusamra, V.B. Bajic, J. Al-Maghrabi, W. Gomaa, M. Hanbazazh, M. Al-Ahwal, A. Al-Harbi, W. Al-Qahtani, S. Hakamy, G. Baba, A. Buhmeida, M. Al-Qahtani, J. Al-Maghrabi, A. Al-Harbi, M. Al-Ahwal, A. Al-Harbi, W. Al-Qahtani, S. Hakamy, G. Baba, A. Buhmeida, M. Al-Qahtani, E.M. Alhathli, S. Karim, N. Salem, H.N. Eldin, H. Abusamra, S. Kumar, M.H. Al-Qahtani, A.A. Alyamani, G. Kalamegam, E.A. Alhwait, M.A. Gari, M.M. Abbas, M.H. Alkaf, H.S. Alsehli, R.A. Kadam, M. Al-Qahtani, R. Gadi, A. Buhmeida, M. Assidi, A. Chaudhary, L. Merdad, S.M. Alfakeeh, E.A. Alhwait, M.A. Gari, M.M. Abbas, M.H. Alkaf, H.S. Alsehli, R. Kadam, G. Kalamegam, R. Ghazala, S. Mathew, M.H. Hamed, M. Assidi, M. Al-Qahtani, I. Qadri, S. Mathew, L. Mira, M. Shaabad, S. Hussain, M. Assidi, M. Abu-Elmagd, M. Al-Qahtani, S. Mathew, M. Shaabad, L. Mira, S. Hussain, M. Assidi, M. Abu-Elmagd, M. Al-Qahtani, A. Rebai, M. Assidi, A. Buhmeida, M. Abu-Elmagd, A. Dallol, J.W. Shay, M.H. Almutairi, A. Ambers, J. Churchill, J. King, M. Stoljarova, H. Gill-King, M. Assidi, M. Abu-Elmagd, A. Buhmeida, M. Al-Qatani, B. Budowle, M. Abu-Elmagd, F. Ahmed, A. Dallol, M. Assidi, T.A. Almagd, S. Hakamy, A. Agarwal, M. Al-Qahtani, A. Abuzenadah, S. Karim, H.-J. Schulten, A.J. Al Sayyad, H.M.A. Farsi, J.A. Al-Maghrabi, A. Buhmaida, Z. Mirza, R. Alotibi, A. Al-Ahmadi, N.A. Alansari, A.A. Albogmi, M.M. Al-Quaiti, F.T. Ashgan, A. Bandah, M.H. Al-Qahtani, R. Satar, M. Rasool, W. Ahmad, N. Nazam, M.I. Lone, M.I. Naseer, M.S. Jamal, S.K. Zaidi, P.N.

Pushparaj, M.A. Jafri, S.A. Ansari, M.H. Alqahtani, H. Bashier, A. Al Qahtani, S. Mathew, A.M. Nour, H. Alkhatabi, A.M.A. Zenadah, A. Buhmeida, M. Assidi, M. Al Qahtani, M. Faheem, S. Mathew, S. Mathew, P.N. Pushparaj, M.H. Al-Qahtani, H.A. Alhadrami, A. Dallol, A. Abuzenadah, I.R. Hussein, A.G. Chaudhary, R.S. Bader, R. Bassiouni, M. Alquaiti, F. Ashgan, H. Schulten, M.N. Alama, M.H. Al Qahtani, M.I. Lone, N. Nizam, W. Ahmad, M.A. Jafri, M. Rasool, S.A. Ansari, M.H. Al-Qahtani, E. Alshihri, M. Abu-Elmagd, L. Alharbi, M. Assidi, M. Al-Qahtani, S. Mathew, P.P. Natesan, M. Al Qahtani, G. Kalamegam, P.N. Pushparaj, F. Khan, R. Kadam, F. Ahmed, M. Assidi, K.H.W. Sait, N. Anfinan, M. Al Qahtani, M.I. Naseer, A.G. Chaudhary, M.S. Jamal, S. Mathew, L.S. Mira, P.N. Pushparaj, S.A. Ansari, M. Rasool, M.H. AlQahtani, M.I. Naseer, A.G. Chaudhary, S. Mathew, L.S. Mira, M.S. Jamal, S. Sogaty, R.I. Bassiouni, M. Rasool, M.H. AlQahtani, M. Rasool, S.A. Ansari, M.S. Jamal, P.N. Pushparaj, A.M.S. Sibiani, W. Ahmad, A. Buhmeida, M.A. Jafri, M.K. Warsi, M.I. Naseer, M.H. Al-Qahtani, null Rubi, K. Kumar, A.A.T. Naqvi, F. Ahmad, M.I. Hassan, M.S. Jamal, M. Rasool, M.H. AlQahtani, A. Ali, J. Jarullah, M. Rasool, A. Buhmeida, S. Khan, G. Abdussami, M. Mahfooz, M.A. Kamal, G.A. Damanhouri, M.S. Jamal, B. Jarullah, J. Jarullah, M.S.S. Jarullah, A. Ali, M. Rasool, M.S. Jamal, M. Assidi, M. Abu-Elmagd, O. Bajouh, P.N. Pushparaj, M. Al-Qahtani, A. Abuzenadah, M.S. Jamal, J. Jarullah, A.E.A. Mathkoor, H.M.A. Alsalmi, A.M.M. Oun, G.A. Damanhauri, M. Rasool, M.H. AlQahtani, M.I. Naseer, M. Rasool, S. Sogaty, A.G. Chudhary, Y.A. Abutalib, D. Merico, S. Walker, C.R. Marshall, M. Zarrei, S.W. Scherer, M.H. Al-Qahtani, M.I. Naseer, M. Faheem, A.G. Chaudhary, M. Rasool, G. Kalamegam, F.T. Ashgan, M. Assidi, F. Ahmed, S.K. Zaidi, M.M. Jan, M.H. Al-Qahtani, M. Al-Zahrani, S. Lary, S. Hakamy, A. Dallol, M. Al-Ahwal, J. Al-Maghrabi, E. Dermitzakis, A. Abuzenadah, A. Buhmeida, M. Al-Qahtani, A.A. Al-refai, M. Saleh, R.I. Yassien, M. Kamel, R.M. Habeb, N. Filimban, A. Dallol, N. Ghannam, M. Al-Qahtani, A.M. Abuzenadah, F. Bibi, S. Akhtar, E.I. Azhar, M. Yasir, M.I. Nasser, A.A. Jiman-Fatani, A. Sawan, R.A. Lahzah, A. Ali, S.A. Hassan, S.E. Hasnain, I.A. Tayubi, H.A. Abujabal, A.O. Magrabi, F. Khan, G. Kalamegam, P.N. Pushparaj, A. Abuzenada, T.A. Kumosani, E. Barbour, M. Al-Qahtani, M. Shabaad, S. Mathew, A. Dallol, A. Merdad, A. Buhmeida, M. Al-Qahtani, M. Assidi, M. Abu-Elmagd, K. Gauthaman, M. Gari, A. Chaudhary, A. Abuzenadah, P.N. Pushparaj, M. Al-Qahtani, S.A. Hassan, I.A. Tayubi, H.M.A. Aljahdali, R. Al Nono, M. Gari, H. Alsehli, F. Ahmed, M. Abbas, G. Kalamegam, M. Al-Qahtani, S. Mathew, F. Khan, M. Rasool, M.S. Jamal, M.I. Naseer, Z. Mirza, S. Karim, S. Ansari, M. Assidi, G. Kalamegam, M. Gari, A. Chaudhary, A. Abuzenadah, P.N. Pushparaj, M. Al-Qahtani, M. Abu-Elmagd, G. Kalamegam, R. Kadam, M.A. Alghamdi, M. Shamy, M. Costa, M.I. Khoder, M. Assidi, P.N. Pushparaj, M. Gari, M. Al-Qahtani, N. Kharrat, S. Belmabrouk, R. Abdelhedi, R. Benmarzoug, M. Assidi, M.H. Al Qahtani, A. Rebai, G. Dhamanhouri, P.N. Pushparaj, A. Noorwali, M.K. Alwasiyah, A. Bahamaid, S. Alfakeeh, A. Alyamani, H. Alsehli, M. Abbas, M. Gari, A. Mobasheri, G. Kalamegam, M. Al-Qahtani, M. Faheem, S. Mathew, P.N. Pushparaj, M.H. Al-Qahtani, S. Mathew, M. Faheem, S. Mathew, P.N. Pushparaj, M.H. Al-Qahtani, M.S. Jamal, S.K. Zaidi, R. Khan, K. Bhatia, M.H. Al-Qahtani, S. Ahmad, I. AslamTayubi, M. Tripathi, S.A. Hassan, R. Shrivastava, I.A. Tayubi, S. Hassan, H.A.S. Abujabal, I. Shah, B. Jarullah, M.S. Jamal, J. Jarullah, I.A. Sheikh, E. Ahmad, M.S. Jamal, M. Rehan, M. Abu-Elmagd, I.A. Tayubi, S.F. AlBasri, O.S. Bajouh, R.F. Turki, A.M. Abuzenadah, G.A. Damanhouri, M.A. Beg, M. Al-Qahtani, S.A.F. Hammoudah, K.M. AlHarbi, L.M. El-Attar, A.M.Z. Darwish, S.M. Ibrahim, A. Dallol, H. Choudhry, A. Abuzenadah, J. Awlia, A. Chaudhary, F. Ahmed, M. Al-Qahtani, M.A. Jafri, M. Abu-Elmagd, M.

Assidi, M. Al-Qahtani, I. khan, M. Yasir, E.I. Azhar, S. Al-basri, E. Barbour, T. Kumosani, F. Khan, G. Kalamegam, P.N. Pushparaj, A. Abuzenada, T.A. Kumosani, E. Barbour, H.M. EL Sayed, E.A. Hafez, H.-J. Schulten, A.H. Elaimi, I.R. Hussein, R.I. Bassiouni, M.K. Alwasiyah, R.F. Wintle, A. Chaudhary, S.W. Scherer, M. Al-Qahtani, Z. Mirza, V.G. Pillai, S. Karim, S. Sharma, P. Kaur, A. Srinivasan, T.P. Singh, M. Al-Qahtani, R. Alotibi, A. Al-Ahmadi, F. Al-Adwani, D. Hussein, S. Karim, M. Al-Sharif, A. Jamal, F. Al-Ghamdi, J. Al-Maghrabi, S.S. Baeesa, M. Bangash, A. Chaudhary, H.-J. Schulten, M. Al-Qahtani, M. Faheem, P.N. Pushparaj, S. Mathew, T.A. Kumosani, G. Kalamegam, M. Al-Qahtani, F.A. Al-Allaf, Z. Abduljaleel, A. Alashwal, M.M. Taher, A. Bouazzaoui, H. Abalkhail, F.A. Ba-Hammam, M. Athar, G. Kalamegam, P.N. Pushparaj, M. Abu-Elmagd, F. Ahmed, K.H. Sait, N. Anfinan, M. Gari, A. Chaudhary, A. Abuzenadah, M. Assidi, M. Al-Qahtani, N.B. Mami, Y.Z. Haffani, M. Medhioub, L. Hamzaoui, A. Cherif, M. Azouz, G. Kalamegam, F. Khan, S. Mathew, M.I. Nasser, M. Rasool, F. Ahmed, P.N. Pushparaj, M. Al-Qahtani, S.A. Turkistany, L.M. Al-harbi, A. Dallol, J. Sabir, A. Chaudhary, A. Abuzenadah, B. Al-Madoudi, B. Al-Aslani, K. Al-Harbi, R. Al-Jahdali, H. Qudaih, E. Al Hamzy, M. Assidi, M. Al Qahtani, A.M. Ilyas, Y. Ahmed, M. Gari, F. Ahmed, M. Alqahtani, N. Salem, S. Karim, E.M. Alhathli, H. Abusamra, H.F.N. Eldin, M.H. Al-Qahtani, S. Kumar, F. Al-Adwani, D. Hussein, M. Al-Sharif, A. Jamal, F. Al-Ghamdi, J. Al-Maghrabi, S.S. Baeesa, M. Bangash, A. Chaudhary, M. Al-Qahtani, H.-J. Schulten, A. Alamandi, R. Alotibi, D. Hussein, S. Karim, J. Al-Maghrabi, F. Al-Ghamdi, A. Jamal, S.S. Baeesa, M. Bangash, A. Chaudhary, H.-J. Schulten, M. Al-Qahtani, O. Subhi, N. Bagatian, S. Karim, A. Al-Johari, O.A. Al-Hamour, H. Al-Aradati, A. Al-Mutawa, F. Al-Mashat, J. Al-Maghrabi, H.-J. Schulten, M. Al-Qahtani, N. Bagatian, O. Subhi, S. Karim, A. Al-Johari, O.A. Al-Hamour, A. Al-Mutawa, H. Al-Aradati, F. Al-Mashat, M. Al-Qahtani, H.-J. Schulten, J. Al-Maghrabi, M.W. shah, M. Yasir, E.I. Azhar, S. Al-Masoodi, Y.Z. Haffani, M. Azouz, E. Khamla, C. Jlassi, A.S. Masmoudi, A. Cherif, L. Belbahri, S. Al-Khayyat, R. Attas, A. Abu-Sanad, M. Abuzinadah, A. Merdad, A. Dallol, A. Chaudhary, M. Al-Qahtani, A. Abuzenadah, H. Bouazzi, C. Trujillo, M.K. Alwasiyah, M. Al-Qahtani, M. Alotaibi, R. Nassir, I.A. Sheikh, M.A. Kamal, E.H. Jiffri, G.M. Ashraf, M.A. Beg, M.A. Aziz, R. Ali, M. Rasool, M.S. Jamal, N. Samman, G. Abdussami, S. Periyasamy, M.K. Warsi, M. Aldress, M. Al Otaibi, Z. Al Yousef, M. Boudjelal, A. Buhmeida, M.H. Al-Qahtani, I. AlAbdulkarim, R. Ghazala, S. Mathew, M.H. Hamed, M. Assidi, M. Al-Qahtani, I. Qadri, I.A. Sheikh, M. Abu-Elmagd, R.F. Turki, G.A. Damanhouri, M.A. Beg, M. Suhail, A. Qureshi, A. Jamal, P.N. Pushparaj, M. Al-Qahtani, I. Qadri, M.Z. El-Readi, S.Y. Eid, M. Wink, A.M. Isa, L. Alnuaim, J. Almutawa, B. Abu-Rafae, S. Alasiri, S. Binsaleh, N. Nazam, M.I. Lone, W. Ahmad, S.A. Ansari, M.H. Alqahtani, Abstracts from the 3rd International Genomic Medicine Conference (3rd IGMC 2015) : Jeddah, Kingdom of Saudi Arabia. 30 November - 3 December 2015, BMC Genomics 17 Suppl 6 (2016) 487. https://doi.org/10.1186/s12864-016-2858-0.

[205] A. Rivers, K. Vaitkus, V. Ibanez, M.A. Ruiz, R. Jagadeeswaran, Y. Saunthararajah, S. Cui, J.D. Engel, J. DeSimone, D. Lavelle, The LSD1 inhibitor RN-1 recapitulates the fetal pattern of hemoglobin synthesis in baboons (P. anubis), Haematologica 101 (2016) 688–697. https://doi.org/10.3324/haematol.2015.140749.

[206] L. Liu, A. Pertsemlidis, L.-H. Ding, M.D. Story, M.H. Steinberg, P. Sebastiani, C. Hoppe, S.K. Ballas, B.S. Pace, Original Research: A case-control genome-wide association study identifies genetic modifiers of fetal hemoglobin in sickle cell disease, Exp Biol Med (Maywood) 241 (2016) 706–718. https://doi.org/10.1177/1535370216642047.

[207] C. Li, Y. Zhou, A. Loberg, S.M. Tahara, P. Malik, V.K. Kalra, Activated Transcription Factor 3 in Association with Histone Deacetylase 6 Negatively Regulates MicroRNA 199a2 Transcription by Chromatin Remodeling and Reduces Endothelin-1 Expression, Mol Cell Biol 36 (2016) 2838–2854. https://doi.org/10.1128/MCB.00345-16.
[208] F.C. Leonardo, A.F. Brugnerotto, I.F. Domingos, K.Y. Fertrin, D.M. de Albuquerque, M.A.C. Bezerra, A.S. Araújo, S.T.O. Saad, F.F. Costa, S. Menzel, N. Conran, S.L. Thein, Reduced rate of sickle-related complications in Brazilian patients carrying HbF-promoting alleles at the BCL11A and HMIP-2 loci, Br J Haematol 173 (2016) 456–460. https://doi.org/10.1111/bjh.13961.
[209] X. Hu, E.H. Jhun, Y. Yao, Y. He, R.E. Molokie, D.J. Wilkie, Z.J. Wang, IL1A rs1800587 associates with chronic noncrisis pain in sickle cell disease, Pharmacogenomics 17 (2016) 1999–2006. https://doi.org/10.2217/pgs-2016-0085.
[210] M.A. Hossain, Y. Shen, I. Knudson, S. Thakur, J.R. Stees, Y. Qiu, B.S. Pace, K.R. Peterson, J. Bungert, Activation of Fetal γ-globin Gene Expression via Direct Protein Delivery of Synthetic Zinc-finger DNA-Binding Domains, Mol Ther Nucleic Acids 5 (2016) e378. https://doi.org/10.1038/mtna.2016.85.
[211] L. Breda, I. Motta, S. Lourenco, C. Gemmo, W. Deng, J.W. Rupon, O.Y. Abdulmalik, D. Manwani, G.A. Blobel, S. Rivella, Forced chromatin looping raises fetal hemoglobin in adult sickle cells to higher levels than pharmacologic inducers, Blood 128 (2016) 1139–1143. https://doi.org/10.1182/blood-2016-01-691089.
[212] C.A. Braghini, F.C. Costa, H. Fedosyuk, R.Y. Neades, L.V. Novikova, M.P. Parker, R.D. Winefield, K.R. Peterson, Original Research: Generation of non-deletional hereditary persistence of fetal hemoglobin β-globin locus yeast artificial chromosome transgenic mouse models: -175 Black HPFH and -195 Brazilian HPFH, Exp Biol Med (Maywood) 241 (2016) 697–705. https://doi.org/10.1177/1535370216636724.
[213] T. Bilgen, D. Canatan, S. Delibas, I. Keser, A Novel Mutation in the Promoter Region of the β-Globin Gene: HBB: c.-127G > C, Hemoglobin 40 (2016) 280–282. https://doi.org/10.1080/03630269.2016.1193513.
[214] T. Ammosova, K. Washington, J. Rotimi, N. Kumari, K.A. Smith, X. Niu, M. Jerebtsova, S. Nekhai, Protein Phosphatase-1 Regulates Expression of Neuregulin-1, Biology (Basel) 5 (2016) 49. https://doi.org/10.3390/biology5040049.
[215] A.O. Akinbami, A.D. Campbell, Z.J. Han, H.-Y. Luo, D.H.K. Chui, M.H. Steinberg, Hereditary Persistence of Fetal Hemoglobin Caused by Single Nucleotide Promoter Mutations in Sickle Cell Trait and Hb SC Disease, Hemoglobin 40 (2016) 64–65. https://doi.org/10.3109/03630269.2015.1080725.
[216] B. Zaker-Kandjani, P. Namdar-Aligoodarzi, A. Azarkeivan, H. Najmabadi, M. Banan, Mutation screening of the Krüppel-like factor 1 gene using single-strand conformational polymorphism in a cohort of Iranian β-thalassemia patients, Hemoglobin 39 (2015) 24–29. https://doi.org/10.3109/03630269.2014.991023.
[217] S. Yamsri, K. Singha, T. Prajantasen, W. Taweenan, G. Fucharoen, K. Sanchaisuriya, S. Fucharoen, A large cohort of β(+)-thalassemia in Thailand: molecular, hematological and diagnostic considerations, Blood Cells Mol Dis 54 (2015) 164–169. https://doi.org/10.1016/j.bcmd.2014.11.008.
[218] G. Thareja, S.E. John, P. Hebbar, K. Behbehani, T.A. Thanaraj, O. Alsmadi, Sequence and analysis of a whole genome from Kuwaiti population subgroup of Persian ancestry, BMC Genomics 16 (2015) 92. https://doi.org/10.1186/s12864-015-1233-x.
[219] R.P. Shamoon, N.A.S. Al-Allawi, M.D. Cappellini, E. Di Pierro, V. Brancaleoni, F. Granata, Molecular Basis of β-Thalassemia Intermedia in Erbil Province of Iraqi Kurdistan, Hemoglobin 39 (2015) 178–183. https://doi.org/10.3109/03630269.2015.1032415.

[220] P. Sebastiani, J.J. Farrell, A. Alsultan, S. Wang, H.L. Edward, H. Shappell, H. Bae, J.N. Milton, C.T. Baldwin, A.M. Al-Rubaish, Z. Naserullah, F. Al-Muhanna, A. Alsuliman, P.K. Patra, L.A. Farrer, D. Ngo, V. Vathipadiekal, D.H.K. Chui, A.K. Al-Ali, M.H. Steinberg, BCL11A enhancer haplotypes and fetal hemoglobin in sickle cell anemia, Blood Cells Mol Dis 54 (2015) 224–230. https://doi.org/10.1016/j.bcmd.2015.01.001.

[221] G.D. Pule, V.J. Ngo Bitoungui, B. Chetcha Chemegni, A.P. Kengne, S. Antonarakis, A. Wonkam, Association between Variants at BCL11A Erythroid-Specific Enhancer and Fetal Hemoglobin Levels among Sickle Cell Disease Patients in Cameroon: Implications for Future Therapeutic Interventions, OMICS 19 (2015) 627–631. https://doi.org/10.1089/omi.2015.0124.

[222] E. Pennisi, GENETICS. An enhanced view of gene control, Science 348 (2015) 1407–1408. https://doi.org/10.1126/science.348.6242.1407.

[223] J.A. Noble, K.C. Duru, A. Guindo, L. Yi, I.G. Imumorin, D.A. Diallo, B.N. Thomas, Interethnic diversity of the CD209 (rs4804803) gene promoter polymorphism in African but not American sickle cell disease, PeerJ 3 (2015) e799. https://doi.org/10.7717/peerj.799.

[224] T. Nitta, F. Kawano, Y. Yamashiro, F. Takagi, T. Murata, T. Tanaka, M. Ferania, C. Adhiyanto, Y. Hattori, A new Krüppel-like factor 1 mutation (c.947G > A or p.C316Y) in humans causes β-thalassemia minor, Hemoglobin 39 (2015) 121–126. https://doi.org/10.3109/03630269.2015.1008702.

[225] R. Nagar, S. Sinha, R. Raman, Genotype-phenotype correlation and report of novel mutations in β-globin gene in thalassemia patients, Blood Cells Mol Dis 55 (2015) 10–14. https://doi.org/10.1016/j.bcmd.2015.03.005.

[226] S.N. Mtatiro, J. Mgaya, T. Singh, H. Mariki, H. Rooks, D. Soka, B. Mmbando, S.L. Thein, J.C. Barrett, J. Makani, S.E. Cox, S. Menzel, Genetic association of fetal-hemoglobin levels in individuals with sickle cell disease in Tanzania maps to conserved regulatory elements within the MYB core enhancer, BMC Med Genet 16 (2015) 4. https://doi.org/10.1186/s12881-015-0148-3.

[227] K.B. Mahesh Kumar, S. Prabha, E. Ramprasad, L.V. Bhaskar, P. Soundararajan, TRPC6 gene promoter polymorphisms in steroid resistant nephrotic syndrome children, J Nephropharmacol 4 (2015) 52–56.

[228] J.-Y. Liau, J.-H. Tsai, C.-Y. Yang, J.-C. Lee, C.-W. Liang, H.-H. Hsu, Y.-M. Jeng, Alternative lengthening of telomeres phenotype in malignant vascular tumors is highly associated with loss of ATRX expression and is frequently observed in hepatic angiosarcomas, Hum Pathol 46 (2015) 1360–1366. https://doi.org/10.1016/j.humpath.2015.05.019.

[229] J.-Y. Liau, J.-H. Tsai, Y.-M. Jeng, J.-C. Lee, H.-H. Hsu, C.-Y. Yang, Leiomyosarcoma with alternative lengthening of telomeres is associated with aggressive histologic features, loss of ATRX expression, and poor clinical outcome, Am J Surg Pathol 39 (2015) 236–244. https://doi.org/10.1097/PAS.0000000000000324.

[230] Z. Li, L. Li, Y. Yao, N. Li, Y. Li, Z. Zhang, F. Yan, H. Qiu, C. Wu, Z. Zhang, A novel promoter mutation (HBB: c.-75G>T) was identified as a cause of β(+)-thalassemia, Hemoglobin 39 (2015) 115–120. https://doi.org/10.3109/03630269.2014.1002844.

[231] C. Li, C.S. Gonsalves, M.-S. Eiymo Mwa Mpollo, P. Malik, S.M. Tahara, V.K. Kalra, MicroRNA 648 Targets ET-1 mRNA and is cotranscriptionally regulated with MICAL3 by PAX5, Mol Cell Biol 35 (2015) 514–528. https://doi.org/10.1128/MCB.01199-14.

[232] I. Krivega, C. Byrnes, J.F. de Vasconcellos, Y.T. Lee, M. Kaushal, A. Dean, J.L. Miller, Inhibition of G9a methyltransferase stimulates fetal hemoglobin production by

facilitating LCR/γ-globin looping, Blood 126 (2015) 665–672. https://doi.org/10.1182/blood-2015-02-629972.
[233] M.A. Herrera, F. De La Fuente-Gonzalo, F.A. González, J.M. Nieto, A.B. Dominguez, A. Villegas, P. Ropero, Identification of a novel mutation in the β-globin gene 3' untranslated region (HBB: c.*+118A > G) in Spain, Hemoglobin 39 (2015) 30–35. https://doi.org/10.3109/03630269.2014.995805.
[234] S. He, Q. Qin, S. Yi, W. Zhou, J. Deng, C. Zheng, B. Chen, First Description of a β-Thalassemia Mutation, -86 (C > G) (HBB: c.-136C > G), in a Chinese Family, Hemoglobin 39 (2015) 448–450. https://doi.org/10.3109/03630269.2015.1070734.
[235] S.M. Hassan, C.L. Harteveld, E. Bakker, P.C. Giordano, Hb Lansing (HBA2: c.264C > G) and a new β promoter transversion [-52 (G > T)]: an attempt to define the phenotype of two mutations found in the Omani population, Hemoglobin 39 (2015) 111–114. https://doi.org/10.3109/03630269.2015.1016615.
[236] C.S. Gonsalves, C. Li, M.-S.E.M. Mpollo, V. Pullarkat, P. Malik, S.M. Tahara, V.K. Kalra, Erythropoietin-mediated expression of placenta growth factor is regulated via activation of hypoxia-inducible factor-1α and post-transcriptionally by miR-214 in sickle cell disease, Biochem J 468 (2015) 409–423. https://doi.org/10.1042/BJ20141138.
[237] C.S. Gonsalves, C. Li, P. Malik, S.M. Tahara, V.K. Kalra, Peroxisome proliferator-activated receptor-α-mediated transcription of miR-301a and miR-454 and their host gene SKA2 regulates endothelin-1 and PAI-1 expression in sickle cell disease, Biosci Rep 35 (2015) e00275. https://doi.org/10.1042/BSR20150190.
[238] S. Farashi, N. Faramarzi Garous, F. Zeinali, S. Vakili, M. Ashki, H. Imanian, H. Najmabadi, A. Azarkeivan, A. Tamaddoni, A 21 Nucleotide Duplication on the α1- and α2-Globin Genes Involves a Variety of Hypochromic Microcytic Anemias, From Mild to Hb H Disease, Hemoglobin 39 (2015) 196–200. https://doi.org/10.3109/03630269.2015.1030757.
[239] A.D. Elmezayen, S.M. Kotb, N.A. Sadek, E.M. Abdalla, β-Globin Mutations in Egyptian Patients With β-Thalassemia, Lab Med 46 (2015) 8–13. https://doi.org/10.1309/LM1AYKG6VE8MLPHG.
[240] L. Dalle Carbonare, A. Matte', M.T. Valenti, A. Siciliano, A. Mori, V. Schweiger, G. Zampieri, L. Perbellini, L. De Franceschi, Hypoxia-reperfusion affects osteogenic lineage and promotes sickle cell bone disease, Blood 126 (2015) 2320–2328. https://doi.org/10.1182/blood-2015-04-641969.
[241] S. Cui, K.-C. Lim, L. Shi, M. Lee, N. Jearawiriyapaisarn, G. Myers, A. Campbell, D. Harro, S. Iwase, R.C. Trievel, A. Rivers, J. DeSimone, D. Lavelle, Y. Saunthararajah, J.D. Engel, The LSD1 inhibitor RN-1 induces fetal hemoglobin synthesis and reduces disease pathology in sickle cell mice, Blood 126 (2015) 386–396. https://doi.org/10.1182/blood-2015-02-626259.
[242] Y.-C. Chou, R.-L. Chen, Z.-S. Lai, J.-S. Song, Y.-S. Chao, C.-K.J. Shen, Pharmacological Induction of Human Fetal Globin Gene in Hydroxyurea-Resistant Primary Adult Erythroid Cells, Mol Cell Biol 35 (2015) 2541–2553. https://doi.org/10.1128/MCB.00035-15.
[243] M.S. Boosalis, J.I. Sangerman, G.L. White, R.F. Wolf, L. Shen, Y. Dai, E. White, L.H. Makala, B. Li, B.S. Pace, M. Nouraie, D.V. Faller, S.P. Perrine, Novel Inducers of Fetal Globin Identified through High Throughput Screening (HTS) Are Active In Vivo in Anemic Baboons and Transgenic Mice, PLoS One 10 (2015) e0144660. https://doi.org/10.1371/journal.pone.0144660.

[244] A.A. Bhanushali, P.K. Patra, S. Pradhan, S.S. Khanka, S. Singh, B.R. Das, Genetics of fetal hemoglobin in tribal Indian patients with sickle cell anemia, Transl Res 165 (2015) 696–703. https://doi.org/10.1016/j.trsl.2015.01.002.
[245] S.Y. Alkindi, A. Pathare, S. Al Zadjali, V. Panjwani, F. Wasim, H. Khan, P. Chopra, R. Krishnamoorthy, S. Alkindi, Serum Total Bilirubin, not Cholelithiasis, is Influenced by UGT1A1 Polymorphism, Alpha Thalassemia and β(s) Haplotype: First Report on Comparison between Arab-Indian and African β(s) Genes, Mediterr J Hematol Infect Dis 7 (2015) e2015060. https://doi.org/10.4084/mjhid.2015.060.
[246] J.S. Waye, B. Eng, M. Hanna, B.-A. Hohenadel, L.N. Nakamura, L. Walker, Non-thalassemic phenotype associated with the -83 (G > A) mutation of the β-globin gene promoter (HBB: c.-133G > A), Hemoglobin 38 (2014) 447–448. https://doi.org/10.3109/03630269.2014.976417.
[247] X. Wang, L. Mendelsohn, H. Rogers, S. Leitman, N. Raghavachari, Y. Yang, Y.Y. Yau, M. Tallack, A. Perkins, J.G. Taylor, C.T. Noguchi, G.J. Kato, Heme-bound iron activates placenta growth factor in erythroid cells via erythroid Krüppel-like factor, Blood 124 (2014) 946–954. https://doi.org/10.1182/blood-2013-11-539718.
[248] R.A. Voit, A. Hendel, S.M. Pruett-Miller, M.H. Porteus, Nuclease-mediated gene editing by homologous recombination of the human globin locus, Nucleic Acids Res 42 (2014) 1365–1378. https://doi.org/10.1093/nar/gkt947.
[249] M. Vinciguerra, C. Passarello, F. Leto, F. Cassarà, M. Cannata, A. Maggio, A. Giambona, Identification of three new nucleotide substitutions in the β-globin gene: laboratoristic approach and impact on genetic counselling for beta-thalassaemia, Eur J Haematol 92 (2014) 444–449. https://doi.org/10.1111/ejh.12267.
[250] R. Stadhouders, S. Aktuna, S. Thongjuea, A. Aghajanirefah, F. Pourfarzad, W. van Ijcken, B. Lenhard, H. Rooks, S. Best, S. Menzel, F. Grosveld, S.L. Thein, E. Soler, HBS1L-MYB intergenic variants modulate fetal hemoglobin via long-range MYB enhancers, J Clin Invest 124 (2014) 1699–1710. https://doi.org/10.1172/JCI71520.
[251] W. Promsote, L. Makala, B. Li, S.B. Smith, N. Singh, V. Ganapathy, B.S. Pace, P.M. Martin, Monomethylfumarate induces γ-globin expression and fetal hemoglobin production in cultured human retinal pigment epithelial (RPE) and erythroid cells, and in intact retina, Invest Ophthalmol Vis Sci 55 (2014) 5382–5393. https://doi.org/10.1167/iovs.14-14179.
[252] T. Prajantasen, N. Teawtrakul, G. Fucharoen, S. Fucharoen, Molecular characterization of a β-thalassemia intermedia patient presenting inferior vena cava thrombosis: interaction of the β-globin erythroid Krüppel-like factor binding site mutation with Hb E and α(+)-thalassemia, Hemoglobin 38 (2014) 451–453. https://doi.org/10.3109/03630269.2014.974608.
[253] K.R. Peterson, F.C. Costa, H. Fedosyuk, R.Y. Neades, A.M. Chazelle, L. Zelenchuk, A.H. Fonteles, P. Dalal, A. Roy, R. Chaguturu, B. Li, B.S. Pace, A cell-based high-throughput screen for novel chemical inducers of fetal hemoglobin for treatment of hemoglobinopathies, PLoS One 9 (2014) e107006. https://doi.org/10.1371/journal.pone.0107006.
[254] S. Menzel, H. Rooks, D. Zelenika, S.N. Mtatiro, A. Gnanakulasekaran, E. Drasar, S. Cox, L. Liu, M. Masood, N. Silver, C. Garner, N. Vasavda, J. Howard, J. Makani, A. Adekile, B. Pace, T. Spector, M. Farrall, M. Lathrop, S.L. Thein, Global genetic architecture of an erythroid quantitative trait locus, HMIP-2, Ann Hum Genet 78 (2014) 434–451. https://doi.org/10.1111/ahg.12077.
[255] M.F. Manchinu, M.F. Marongiu, D. Poddie, C. Casu, V. Latini, M. Simbula, R. Galanello, P. Moi, A. Cao, S. Porcu, M.S. Ristaldi, In vivo activation of the human δ-

globin gene: the therapeutic potential in β-thalassemic mice, Haematologica 99 (2014) 76–84. https://doi.org/10.3324/haematol.2012.082768.
[256] C. Li, M.-S.E.M. Mpollo, C.S. Gonsalves, S.M. Tahara, P. Malik, V.K. Kalra, Peroxisome proliferator-activated receptor-α-mediated transcription of miR-199a2 attenuates endothelin-1 expression via hypoxia-inducible factor-1α, J Biol Chem 289 (2014) 36031–36047. https://doi.org/10.1074/jbc.M114.600775.
[257] N. Kumari, S. Iordanskiy, D. Kovalskyy, D. Breuer, X. Niu, X. Lin, M. Xu, K. Gavrilenko, F. Kashanchi, S. Dhawan, S. Nekhai, Phenyl-1-Pyridin-2yl-ethanone-based iron chelators increase IκB-α expression, modulate CDK2 and CDK9 activities, and inhibit HIV-1 transcription, Antimicrob Agents Chemother 58 (2014) 6558–6571. https://doi.org/10.1128/AAC.02918-14.
[258] Z. He, D. Song, S. van Zalen, J.E. Russell, Structural determinants of human ζ-globin mRNA stability, J Hematol Oncol 7 (2014) 35. https://doi.org/10.1186/1756-8722-7-35.
[259] C.S. Gonsalves, S. Crable, S. Chandra, W. Li, V.K. Kalra, C.H. Joiner, Angiogenic growth factors augment K-Cl cotransporter expression in erythroid cells via hypoxia-inducible factor-1α, Am J Hematol 89 (2014) 273–281. https://doi.org/10.1002/ajh.23631.
[260] O. Gilad, O. Dgany, S. Noy-Lotan, T. Krasnov, S. Elitzur, S. Pissard, I. Kventsel, J. Yacobovich, H. Tamary, Characterization of two unique α-globin gene cluster deletions causing α-thalassemia in Israeli Arabs, Hemoglobin 38 (2014) 319–324. https://doi.org/10.3109/03630269.2014.954668.
[261] P.S. Dabke, R.B. Colah, K.K. Ghosh, A.H. Nadkarni, Role of co-inherited Gilbert syndrome on hyperbilirubinemia in Indian beta thalassemia patients, Hematology 19 (2014) 388–392. https://doi.org/10.1179/1607845413Y.0000000142.
[262] A. Coelho, A. Dias, A. Morais, B. Nunes, E. Ferreira, I. Picanço, P. Faustino, J. Lavinha, Genetic variation in CD36, HBA, NOS3 and VCAM1 is associated with chronic haemolysis level in sickle cell anaemia: a longitudinal study, Eur J Haematol 92 (2014) 237–243. https://doi.org/10.1111/ejh.12226.
[263] G.L. Cardoso, I.G. Diniz, A.N.L.M. da Silva, D.A. Cunha, J.S. da Silva Junior, C.T.C. Uchôa, S.E.B. dos Santos, S.M.S. Trindade, M. do S. de O. Cardoso, J.F. Guerreiro, DNA polymorphisms at BCL11A, HBS1L-MYB and Xmn1-HBG2 site loci associated with fetal hemoglobin levels in sickle cell anemia patients from Northern Brazil, Blood Cells Mol Dis 53 (2014) 176–179. https://doi.org/10.1016/j.bcmd.2014.07.006.
[264] V. Thomas, B. Mazard, C. Garcia, P. Lacan, M.-C. Gagnieu, P. Joly, UGT1A1 (TA)n genotyping in sickle-cell disease: high resolution melting (HRM) curve analysis or direct sequencing, what is the best way?, Clin Chim Acta 424 (2013) 258–260. https://doi.org/10.1016/j.cca.2013.06.023.
[265] C. Tafrali, A. Paizi, J. Borg, M. Radmilovic, M. Bartsakoulia, E. Giannopoulou, O. Giannakopoulou, M. Stojiljkovic-Petrovic, B. Zukic, K. Poulas, E.F. Stavrou, P. Lambropoulou, A. Kourakli, A.E. Felice, A. Papachatzopoulou, S. Philipsen, S. Pavlovic, M. Georgitsi, G.P. Patrinos, Genomic variation in the MAP3K5 gene is associated with β-thalassemia disease severity and hydroxyurea treatment efficacy, Pharmacogenomics 14 (2013) 469–483. https://doi.org/10.2217/pgs.13.31.
[266] M. Suzuki, H. Yamazaki, H.Y. Mukai, H. Motohashi, L. Shi, O. Tanabe, J.D. Engel, M. Yamamoto, Disruption of the Hbs1l-Myb locus causes hereditary persistence of fetal hemoglobin in a mouse model, Mol Cell Biol 33 (2013) 1687–1695. https://doi.org/10.1128/MCB.01617-12.
[267] L. Song, X. Li, G.R. Jayandharan, Y. Wang, G.V. Aslanidi, C. Ling, L. Zhong, G. Gao, M.C. Yoder, C. Ling, M. Tan, A. Srivastava, High-efficiency transduction of

primary human hematopoietic stem cells and erythroid lineage-restricted expression by optimized AAV6 serotype vectors in vitro and in a murine xenograft model in vivo, PLoS One 8 (2013) e58757. https://doi.org/10.1371/journal.pone.0058757.
[268] L. Shi, S. Cui, J.D. Engel, O. Tanabe, Lysine-specific demethylase 1 is a therapeutic target for fetal hemoglobin induction, Nat Med 19 (2013) 291–294. https://doi.org/10.1038/nm.3101.
[269] V.A. Sheehan, Z. Luo, J.M. Flanagan, T.A. Howard, B.W. Thompson, W.C. Wang, A. Kutlar, R.E. Ware, BABY HUG Investigators, Genetic modifiers of sickle cell anemia in the BABY HUG cohort: influence on laboratory and clinical phenotypes, Am J Hematol 88 (2013) 571–576. https://doi.org/10.1002/ajh.23457.
[270] P. Ropero, F.A. González, E. Cela, C. Beléndez, A. Cervera, J. Martínez-Nieto, F. de la Fuente-Gonzalo, L. Vinuesa, A. Villegas, J. Díaz-Mediavilla, Association in cis of the mutations +20 (C>T) in the 5' untranslated region and IVS-II-745 (C>G) on the β-globin gene, Hemoglobin 37 (2013) 112–118. https://doi.org/10.3109/03630269.2013.766620.
[271] X. Qian, J. Chen, D. Zhao, L. Guo, X. Qian, Plastrum testudinis induces γ-globin gene expression through epigenetic histone modifications within the γ-globin gene promoter via activation of the p38 MAPK signaling pathway, Int J Mol Med 31 (2013) 1418–1428. https://doi.org/10.3892/ijmm.2013.1338.
[272] F. Pourfarzad, A. Aghajanirefah, E. de Boer, S. Ten Have, T. Bryn van Dijk, S. Kheradmandkia, R. Stadhouders, S. Thongjuea, E. Soler, N. Gillemans, M. von Lindern, J. Demmers, S. Philipsen, F. Grosveld, Locus-specific proteomics by TChP: targeted chromatin purification, Cell Rep 4 (2013) 589–600. https://doi.org/10.1016/j.celrep.2013.07.004.
[273] D. Ngo, H. Bae, M.H. Steinberg, P. Sebastiani, N. Solovieff, C.T. Baldwin, E. Melista, S. Safaya, L.A. Farrer, A.M. Al-Suliman, W.H. Albuali, M.H. Al Bagshi, Z. Naserullah, I. Akinsheye, P. Gallagher, H. Luo, D.H.K. Chui, J.J. Farrell, A.K. Al-Ali, A. Alsultan, Fetal hemoglobin in sickle cell anemia: genetic studies of the Arab-Indian haplotype, Blood Cells Mol Dis 51 (2013) 22–26. https://doi.org/10.1016/j.bcmd.2012.12.005.
[274] M. Neishabury, F. Zamani, E. Keyhani, A. Azarkeivan, S.S. Abedini, M.S. Eslami, S.T. Kakroodi, M.J. Vesiehsari, H. Najmabadi, The influence of the BCL11A polymorphism on the phenotype of patients with beta thalassemia could be affected by the beta globin locus control region and/or the Xmn1-HBG2 genotypic background, Blood Cells Mol Dis 51 (2013) 80–84. https://doi.org/10.1016/j.bcmd.2013.02.007.
[275] A. Kutlar, M.E. Reid, A. Inati, A.T. Taher, M.R. Abboud, A. El-Beshlawy, G.R. Buchanan, H. Smith, K.I. Ataga, S.P. Perrine, R.G. Ghalie, A dose-escalation phase IIa study of 2,2-dimethylbutyrate (HQK-1001), an oral fetal globin inducer, in sickle cell disease, Am J Hematol 88 (2013) E255-260. https://doi.org/10.1002/ajh.23533.
[276] L. Jouini, C.A. Sahli, N. Laaouini, F. Ouali, I. Ben Youssef, B. Dakhlaoui, R. Othmeni, F. Ouennich, S. Hadj Fredj, H. Siala, M. Becher, N.E. Toumi, S. Fattoum, R. Hafsia, A. Bibi, T. Messaoud, Association between clinical expression and molecular heterogeneity in β-thalassemia Tunisian patients, Mol Biol Rep 40 (2013) 6205–6212. https://doi.org/10.1007/s11033-013-2732-y.
[277] Y. He, G. Rank, M. Zhang, J. Ju, R. Liu, Z. Xu, F. Brown, L. Cerruti, C. Ma, R. Tan, S.M. Jane, Q. Zhao, Induction of human fetal hemoglobin expression by adenosine-2',3'-dialdehyde, J Transl Med 11 (2013) 14. https://doi.org/10.1186/1479-5876-11-14.

[278] Z. Hamad, A. Aljedai, R. Halwani, A. AlSultan, UGT1A1 promoter polymorphism associated with serum bilirubin level in Saudi patients with sickle cell disease, Ann Saudi Med 33 (2013) 372–376. https://doi.org/10.5144/0256-4947.2013.372.
[279] A. Fettah, C. Bayram, N. Yarali, P. Isik, A. Kara, V. Culha, B. Tunc, Beta-globin Gene Mutations in Turkish Children with Beta-Thalassemia: Results from a Single Center Study, Mediterr J Hematol Infect Dis 5 (2013) e2013055. https://doi.org/10.4084/MJHID.2013.055.
[280] J.Y. Chin, F. Reza, P.M. Glazer, Triplex-forming peptide nucleic acids induce heritable elevations in gamma-globin expression in hematopoietic progenitor cells, Mol Ther 21 (2013) 580–587. https://doi.org/10.1038/mt.2012.262.
[281] L. Chaouch, E. Talbi, I. Moumni, A. Ben Chaabene, M. Kalai, D. Chaouachi, F. Mallouli, A. Ghanem, S. Abbes, Early complication in sickle cell anemia children due to A(TA)nTAA polymorphism at the promoter of UGT1A1 gene, Dis Markers 35 (2013) 67–72. https://doi.org/10.1155/2013/173474.
[282] L. Chaouch, E. Talbi, I. Moumni, A. Ben Chaabene, M. Kalai, D. Chaouachi, F. Mallouli, A. Ghanem, S. Abbes, Early complication in Sickle Cell Anemia children due to A(TA)<formula>_n</formula> TAA polymorphism at the promoter of UGT1A1 gene, Dis Markers (2013). https://doi.org/10.1155/2013/173474.
[283] T. Bilgen, O.A. Clark, Z. Ozturk, M. Akif Yesilipek, I. Keser, Two novel mutations in the 3' untranslated region of the beta-globin gene that are associated with the mild phenotype of beta thalassemia, Int J Lab Hematol 35 (2013) 26–30. https://doi.org/10.1111/j.1751-553X.2012.01456.x.
[284] S. AlFadhli, H. Al-Jafer, M. Hadi, M. Al-Mutairi, R. Nizam, The effect of UGT1A1 promoter polymorphism in the development of hyperbilirubinemia and cholelithiasis in hemoglobinopathy patients, PLoS One 8 (2013) e77681. https://doi.org/10.1371/journal.pone.0077681.
[285] K. Wilkinson, S. Harris, P. Gaur, A. Haile, R. Armour, G. Teramura, M. Delaney, Molecular blood typing augments serologic testing and allows for enhanced matching of red blood cells for transfusion in patients with sickle cell disease, Transfusion 52 (2012) 381–388. https://doi.org/10.1111/j.1537-2995.2011.03288.x.
[286] J.N. Milton, P. Sebastiani, N. Solovieff, S.W. Hartley, P. Bhatnagar, D.E. Arking, D.A. Dworkis, J.F. Casella, E. Barron-Casella, C.J. Bean, W.C. Hooper, M.R. DeBaun, M.E. Garrett, K. Soldano, M.J. Telen, A. Ashley-Koch, M.T. Gladwin, C.T. Baldwin, M.H. Steinberg, E.S. Klings, A genome-wide association study of total bilirubin and cholelithiasis risk in sickle cell anemia, PLoS One 7 (2012) e34741. https://doi.org/10.1371/journal.pone.0034741.
[287] A. Mejri, H. Siala, F. Ouali, A. Bibi, T. Messaoud, Identification of candidate genes involved in clinical variability among Tunisian patients with β-thalassemia, Gene 506 (2012) 166–172. https://doi.org/10.1016/j.gene.2012.06.078.
[288] N. Mahdi, T.M. Abu-Hijleh, F.M. Abu-Hijleh, M.S. Sater, K. Al-Ola, W.Y. Almawi, Protein Z polymorphisms associated with vaso-occlusive crisis in young sickle cell disease patients, Ann Hematol 91 (2012) 1215–1220. https://doi.org/10.1007/s00277-012-1474-6.
[289] A. Kutlar, K. Ataga, M. Reid, E.P. Vichinsky, L. Neumayr, L. Blair-Britt, R. Labotka, J. Glass, J.R. Keefer, W.A. Wargin, R. Berenson, S.P. Perrine, A phase 1/2 trial of HQK-1001, an oral fetal globin inducer, in sickle cell disease, Am J Hematol 87 (2012) 1017–1021. https://doi.org/10.1002/ajh.23306.
[290] M. Hino, Y. Yamashiro, Y. Hattori, H. Ito, T. Nitta, C. Adhiyanto, M. Matar, M. Naveed, Identification of a novel mutation in the β-globin gene 3' untranslated region

[+1,506 (A>C)] in a Japanese male with a heterozygous β-thalassemia phenotype, Hemoglobin 36 (2012) 170–176. https://doi.org/10.3109/03630269.2011.647186.
[291] E. Fibach, E. Prus, N. Bianchi, C. Zuccato, G. Breveglieri, F. Salvatori, A. Finotti, M. Lipucci di Paola, E. Brognara, I. Lampronti, M. Borgatti, R. Gambari, Resveratrol: Antioxidant activity and induction of fetal hemoglobin in erythroid cells from normal donors and β-thalassemia patients, Int J Mol Med 29 (2012) 974–982. https://doi.org/10.3892/ijmm.2012.928.
[292] F.C. Costa, H. Fedosyuk, R. Neades, J.B. de Los Rios, C.F. Barbas, K.R. Peterson, Induction of Fetal Hemoglobin In Vivo Mediated by a Synthetic γ-Globin Zinc Finger Activator, Anemia 2012 (2012) 507894. https://doi.org/10.1155/2012/507894.
[293] D. Breuer, A. Kotelkin, T. Ammosova, N. Kumari, A. Ivanov, A.V. Ilatovskiy, M. Beullens, P.R. Roane, M. Bollen, M.G. Petukhov, F. Kashanchi, S. Nekhai, CDK2 regulates HIV-1 transcription by phosphorylation of CDK9 on serine 90, Retrovirology 9 (2012) 94. https://doi.org/10.1186/1742-4690-9-94.
[294] R. Bertrand, Nitric oxide-mediated suppression of 2,3-bisphosphoglycerate synthesis: therapeutic relevance for environmental hypoxia and sickle cell disease, Med Hypotheses 79 (2012) 315–318. https://doi.org/10.1016/j.mehy.2012.05.020.
[295] C.J. Bean, S.L. Boulet, D. Ellingsen, M.E. Pyle, E.A. Barron-Casella, J.F. Casella, A.B. Payne, J. Driggers, H.A. Trau, G. Yang, K. Jones, S.F. Ofori-Acquah, W.C. Hooper, M.R. DeBaun, Heme oxygenase-1 gene promoter polymorphism is associated with reduced incidence of acute chest syndrome among children with sickle cell disease, Blood 120 (2012) 3822–3828. https://doi.org/10.1182/blood-2011-06-361642.
[296] A. Alsultan, D.A. Ngo, J.J. Farrell, I. Akinsheye, N. Solovieff, H.A. Ghabbour, A. Al-Ali, A. Alsuliman, M. Al-Baghshi, W. Albu-Ali, M. Alabdulaali, C.T. Baldwin, L.A. Farrer, H. Luo, E. Melista, S. Safaya, M. Nwaru, D.H.K. Chui, M.H. Steinberg, A functional promoter polymorphism of the δ-globin gene is a specific marker of the Arab-Indian haplotype, Am J Hematol 87 (2012) 824–826. https://doi.org/10.1002/ajh.23239.
[297] M.T. Akbari, M. Hamid, Identification of α-globin chain variants: a report from Iran, Arch Iran Med 15 (2012) 564–567.
[298] J. Zhu, K. Chin, W. Aerbajinai, C. Trainor, P. Gao, G.P. Rodgers, Recombinant erythroid Kruppel-like factor fused to GATA1 up-regulates delta- and gamma-globin expression in erythroid cells, Blood 117 (2011) 3045–3052. https://doi.org/10.1182/blood-2010-07-294751.
[299] J.S. Waye, L.M. Nakamura-Garrett, B. Eng, E. Kanavakis, J. Traeger-Synodinos, β+-Thalassemia trait due to a novel mutation in the β-globin gene promoter: -26 (A>C) [HBB c.-76A>C], Hemoglobin 35 (2011) 84–86. https://doi.org/10.3109/03630269.2010.529744.
[300] A.L. Walker, S. Steward, T.A. Howard, N. Mortier, M. Smeltzer, Y.-D. Wang, R.E. Ware, Epigenetic and molecular profiles of erythroid cells after hydroxyurea treatment in sickle cell anemia, Blood 118 (2011) 5664–5670. https://doi.org/10.1182/blood-2011-07-368746.
[301] J. Van Ziffle, W. Yang, F.F. Chehab, Homozygous deletion of six olfactory receptor genes in a subset of individuals with Beta-thalassemia, PLoS One 6 (2011) e17327. https://doi.org/10.1371/journal.pone.0017327.
[302] V. Sebastiano, M.L. Maeder, J.F. Angstman, B. Haddad, C. Khayter, D.T. Yeo, M.J. Goodwin, J.S. Hawkins, C.L. Ramirez, L.F.Z. Batista, S.E. Artandi, M. Wernig, J.K. Joung, In situ genetic correction of the sickle cell anemia mutation in human induced pluripotent stem cells using engineered zinc finger nucleases, Stem Cells 29 (2011) 1717–1726. https://doi.org/10.1002/stem.718.

[303] L. Martin, V. Douet, C.M. VanWart, M.B. Heller, O. Le Saux, A mouse model of β-thalassemia shows a liver-specific down-regulation of Abcc6 expression, Am J Pathol 178 (2011) 774–783. https://doi.org/10.1016/j.ajpath.2010.10.004.
[304] E.R. Macari, C.H. Lowrey, Induction of human fetal hemoglobin via the NRF2 antioxidant response signaling pathway, Blood 117 (2011) 5987–5997. https://doi.org/10.1182/blood-2010-10-314096.
[305] M. Hamid, F. Mahjoubi, M.T. Akbari, H. Khanahmad, F. Jamshidi, S. Zeinali, M. Karimipoor, Transient expression assay of Agamma-588 (A/G) mutations in the K562 cell line, Iran Biomed J 15 (2011) 15–21.
[306] M. Hamid, M.T. Akbari, A 13-bp deletion in the 3' untranslated region of the β-globin gene causes β-thalassemia major in compound heterozygosity with IVSII-1 mutation, Med Princ Pract 20 (2011) 488–490. https://doi.org/10.1159/000328425.
[307] A. Haj Khelil, M. Morinière, S. Laradi, A. Khelif, P. Perrin, J. Ben Chibani, F. Baklouti, Xmn I polymorphism associated with concomitant activation of Gγ and Aγ globin gene transcription on a β0-thalassemia chromosome, Blood Cells Mol Dis 46 (2011) 133–138. https://doi.org/10.1016/j.bcmd.2010.11.002.
[308] Z. Gatalica, S.L. Lilleberg, F.A. Monzon, M.S. Koul, J.A. Bridge, J. Knezetic, B. Legendre, P. Sharma, P.A. McCue, Renal medullary carcinomas: histopathologic phenotype associated with diverse genotypes, Hum Pathol 42 (2011) 1979–1988. https://doi.org/10.1016/j.humpath.2011.02.026.
[309] D.A. Dworkis, E.S. Klings, N. Solovieff, G. Li, J.N. Milton, S.W. Hartley, E. Melista, J. Parente, P. Sebastiani, M.H. Steinberg, C.T. Baldwin, Severe sickle cell anemia is associated with increased plasma levels of TNF-R1 and VCAM-1, Am J Hematol 86 (2011) 220–223. https://doi.org/10.1002/ajh.21928.
[310] Z. Debebe, T. Ammosova, D. Breuer, D.B. Lovejoy, D.S. Kalinowski, K. Kumar, M. Jerebtsova, P. Ray, F. Kashanchi, V.R. Gordeuk, D.R. Richardson, S. Nekhai, Iron chelators of the di-2-pyridylketone thiosemicarbazone and 2-benzoylpyridine thiosemicarbazone series inhibit HIV-1 transcription: identification of novel cellular targets--iron, cyclin-dependent kinase (CDK) 2, and CDK9, Mol Pharmacol 79 (2011) 185–196. https://doi.org/10.1124/mol.110.069062.
[311] T. Bilgen, D. Canatan, Y. Arıkan, A. Yeşilipek, İ. Keser, The effect of HBB: c.*+96T>C (3'UTR +1570 T>C) on the mild b-thalassemia intermedia phenotype, Turk J Haematol 28 (2011) 219–222. https://doi.org/10.5152/tjh.2011.57.
[312] S.B. Anyona, P. Kempaiah, E. Raballah, C. Ouma, T. Were, G.C. Davenport, S.N. Konah, J.M. Vulule, J.B. Hittner, C.W. Gichuki, J.M. Ong'echa, D.J. Perkins, Functional promoter haplotypes of interleukin-18 condition susceptibility to severe malarial anemia and childhood mortality, Infect Immun 79 (2011) 4923–4932. https://doi.org/10.1128/IAI.05601-11.
[313] S. Al Zadjali, Y. Wali, F. Al Lawatiya, D. Gravell, S. Alkindi, K. Al Falahi, R. Krishnamoorthy, S. Daar, The β-globin promoter -71 C>T mutation is a β+ thalassemic allele, Eur J Haematol 87 (2011) 457–460. https://doi.org/10.1111/j.1600-0609.2011.01687.x.
[314] A.D. Adekile, Limitations of Hb F as a phenotypic modifier in sickle cell disease: study of Kuwaiti Arab patients, Hemoglobin 35 (2011) 607–617. https://doi.org/10.3109/03630269.2011.617230.
[315] A. Wilber, U. Tschulena, P.W. Hargrove, Y.-S. Kim, D.A. Persons, C.F. Barbas, A.W. Nienhuis, A zinc-finger transcriptional activator designed to interact with the gamma-globin gene promoters enhances fetal hemoglobin production in primary human adult erythroblasts, Blood 115 (2010) 3033–3041. https://doi.org/10.1182/blood-2009-08-240556.

[316] Z. Wang, W. Yu, Y. Li, X. Shang, X. Zhang, F. Xiong, X. Xu, Analysis of alpha-hemoglobin-stabilizing protein (AHSP) gene as a genetic modifier to the phenotype of beta-thalassemia in Southern China, Blood Cells Mol Dis 45 (2010) 128–132. https://doi.org/10.1016/j.bcmd.2010.04.005.
[317] M. Savy, B.J. Hennig, C.P. Doherty, A.J. Fulford, R. Bailey, M.J. Holland, G. Sirugo, K.A. Rockett, D.P. Kwiatkowski, A.M. Prentice, S.E. Cox, Haptoglobin and sickle cell polymorphisms and risk of active trachoma in Gambian children, PLoS One 5 (2010) e11075. https://doi.org/10.1371/journal.pone.0011075.
[318] G. Rank, L. Cerruti, R.J. Simpson, R.L. Moritz, S.M. Jane, Q. Zhao, Identification of a PRMT5-dependent repressor complex linked to silencing of human fetal globin gene expression, Blood 116 (2010) 1585–1592. https://doi.org/10.1182/blood-2009-10-251116.
[319] R. Petruzzelli, S. Gaudino, G. Amendola, R. Sessa, S. Puzone, R. Di Concilio, G. d’Urzo, M. Amendolara, P. Izzo, M. Grosso, Role of the cold shock domain protein A in the transcriptional regulation of HBG expression, Br J Haematol 150 (2010) 689–699. https://doi.org/10.1111/j.1365-2141.2010.08303.x.
[320] N. Patel, N. Sundaram, M. Yang, C. Madigan, V.K. Kalra, P. Malik, Placenta growth factor (PlGF), a novel inducer of plasminogen activator inhibitor-1 (PAI-1) in sickle cell disease (SCD), J Biol Chem 285 (2010) 16713–16722. https://doi.org/10.1074/jbc.M110.101691.
[321] N. Patel, V.K. Kalra, Placenta growth factor-induced early growth response 1 (Egr-1) regulates hypoxia-inducible factor-1alpha (HIF-1alpha) in endothelial cells, J Biol Chem 285 (2010) 20570–20579. https://doi.org/10.1074/jbc.M110.119495.
[322] T.K.T. Nguyen, P. Joly, C. Bardel, M. Moulsma, N. Bonello-Palot, A. Francina, The XmnI (G)gamma polymorphism influences hemoglobin F synthesis contrary to BCL11A and HBS1L-MYB SNPs in a cohort of 57 beta-thalassemia intermedia patients, Blood Cells Mol Dis 45 (2010) 124–127. https://doi.org/10.1016/j.bcmd.2010.04.002.
[323] T.F. Mendonça, M.C.V.C. Oliveira, L.R.S. Vasconcelos, L.M.M.B. Pereira, P. Moura, M. a. C. Bezerra, M.N.N. Santos, A.S. Araújo, M.S.M. Cavalcanti, Association of variant alleles of MBL2 gene with vasoocclusive crisis in children with sickle cell anemia, Blood Cells Mol Dis 44 (2010) 224–228. https://doi.org/10.1016/j.bcmd.2010.02.004.
[324] P. Mali, B.-K. Chou, J. Yen, Z. Ye, J. Zou, S. Dowey, R.A. Brodsky, J.E. Ohm, W. Yu, S.B. Baylin, K. Yusa, A. Bradley, D.J. Meyers, C. Mukherjee, P.A. Cole, L. Cheng, Butyrate greatly enhances derivation of human induced pluripotent stem cells by promoting epigenetic remodeling and the expression of pluripotency-associated genes, Stem Cells 28 (2010) 713–720. https://doi.org/10.1002/stem.402.
[325] K.Y. Italia, F.F. Jijina, D. Jain, R. Merchant, A.H. Nadkarni, M. Mukherjee, K. Ghosh, R.B. Colah, The effect of UGT1A1 promoter polymorphism on bilirubin response to hydroxyurea therapy in hemoglobinopathies, Clin Biochem 43 (2010) 1329–1332. https://doi.org/10.1016/j.clinbiochem.2010.08.006.
[326] M. Halvorsen, J.S. Martin, S. Broadaway, A. Laederach, Disease-associated mutations that alter the RNA structural ensemble, PLoS Genet 6 (2010) e1001074. https://doi.org/10.1371/journal.pgen.1001074.
[327] C.S. Gonsalves, V.K. Kalra, Hypoxia-mediated expression of 5-lipoxygenase-activating protein involves HIF-1alpha and NF-kappaB and microRNAs 135a and 199a-5p, J Immunol 184 (2010) 3878–3888. https://doi.org/10.4049/jimmunol.0902594.

[328] C. Gonsalves, V.K. Kalra, Endothelin-1-induced macrophage inflammatory protein-1beta expression in monocytic cells involves hypoxia-inducible factor-1alpha and AP-1 and is negatively regulated by microRNA-195, J Immunol 185 (2010) 6253–6264. https://doi.org/10.4049/jimmunol.1000660.
[329] M. Cavazzana-Calvo, E. Payen, O. Negre, G. Wang, K. Hehir, F. Fusil, J. Down, M. Denaro, T. Brady, K. Westerman, R. Cavallesco, B. Gillet-Legrand, L. Caccavelli, R. Sgarra, L. Maouche-Chrétien, F. Bernaudin, R. Girot, R. Dorazio, G.-J. Mulder, A. Polack, A. Bank, J. Soulier, J. Larghero, N. Kabbara, B. Dalle, B. Gourmel, G. Socie, S. Chrétien, N. Cartier, P. Aubourg, A. Fischer, K. Cornetta, F. Galacteros, Y. Beuzard, E. Gluckman, F. Bushman, S. Hacein-Bey-Abina, P. Leboulch, Transfusion independence and HMGA2 activation after gene therapy of human β-thalassaemia, Nature 467 (2010) 318–322. https://doi.org/10.1038/nature09328.
[330] J.D. Belcher, J.V. Vineyard, C.M. Bruzzone, C. Chen, J.D. Beckman, J. Nguyen, C.J. Steer, G.M. Vercellotti, Heme oxygenase-1 gene delivery by Sleeping Beauty inhibits vascular stasis in a murine model of sickle cell disease, J Mol Med (Berl) 88 (2010) 665–675. https://doi.org/10.1007/s00109-010-0613-6.
[331] C.G. Barbosa, N.J. Goncalves-Santos, S.B. Souza-Ribeiro, J.P. Moura-Neto, D. Takahashi, D.O. Silva, A.F. Hurtado-Guerrero, M.G. Reis, M.S. Goncalves, Promoter region sequence differences in the A and G gamma globin genes of Brazilian sickle cell anemia patients, Braz J Med Biol Res 43 (2010) 705–711. https://doi.org/10.1590/s0100-879x2010007500062.
[332] X.S. Xu, X. Hong, G. Wang, Induction of endogenous gamma-globin gene expression with decoy oligonucleotide targeting Oct-1 transcription factor consensus sequence, J Hematol Oncol 2 (2009) 15. https://doi.org/10.1186/1756-8722-2-15.
[333] S. Safaya, E.S. Klings, A. Odhiambo, G. Li, H.W. Farber, M.H. Steinberg, Effect of sodium butyrate on lung vascular TNFSF15 (TL1A) expression: differential expression patterns in pulmonary artery and microvascular endothelial cells, Cytokine 46 (2009) 72–78. https://doi.org/10.1016/j.cyto.2008.12.013.
[334] T.I. Pestina, P.W. Hargrove, D. Jay, J.T. Gray, K.M. Boyd, D.A. Persons, Correction of murine sickle cell disease using gamma-globin lentiviral vectors to mediate high-level expression of fetal hemoglobin, Mol Ther 17 (2009) 245–252. https://doi.org/10.1038/mt.2008.259.
[335] N. Patel, C.S. Gonsalves, M. Yang, P. Malik, V.K. Kalra, Placenta growth factor induces 5-lipoxygenase-activating protein to increase leukotriene formation in sickle cell disease, Blood 113 (2009) 1129–1138. https://doi.org/10.1182/blood-2008-07-169821.
[336] I. Lampronti, N. Bianchi, C. Zuccato, F. Dall'Acqua, D. Vedaldi, G. Viola, R. Potenza, F. Chiavilli, G. Breveglieri, M. Borgatti, A. Finotti, G. Feriotto, F. Salvatori, R. Gambari, Increase in gamma-globin mRNA content in human erythroid cells treated with angelicin analogs, Int J Hematol 90 (2009) 318–327. https://doi.org/10.1007/s12185-009-0422-2.
[337] M. Haghi, A.A.H. Feizi, C.L. Harteveld, N. Pouladi, M.A.H. Feizi, Homozygosity for a rare beta 0-thalassemia mutation [frameshift codons 25/26 (+T)] causes beta-thalassemia intermedia in an Iranian family, Hemoglobin 33 (2009) 75–80. https://doi.org/10.1080/03630260802683377.
[338] A. Guerrini, I. Lampronti, N. Bianchi, C. Zuccato, G. Breveglieri, F. Salvatori, I. Mancini, D. Rossi, R. Potenza, F. Chiavilli, G. Sacchetti, R. Gambari, M. Borgatti, Bergamot (Citrus bergamia Risso) fruit extracts as γ-globin gene expression inducers: phytochemical and functional perspectives, J Agric Food Chem 57 (2009) 4103–4111. https://doi.org/10.1021/jf803489p.

[339] O.P. Dossou-Yovo, C. Lapoumeroulie, M. Hauchecorne, I. Zaccaria, R. Ducrocq, R. Krishnamoorthy, M.C. Rahimy, J. Elion, Variants of the mannose-binding lectin gene in the Benin population: heterozygosity for the p.G57E allele may confer a selective advantage. 2007, Hum Biol 81 (2009) 899–909. https://doi.org/10.3378/027.081.0630.
[340] A.F. da Cunha, A.F. Brugnerotto, M.A.F. Corat, E.E. Devlin, A.P. Gimenes, M.B. de Melo, L.A.C. Passos, D. Bodine, S.T.O. Saad, F.F. Costa, High levels of human gamma-globin are expressed in adult mice carrying a transgene of the Brazilian type of hereditary persistence of fetal hemoglobin ((A)gamma -195), Hemoglobin 33 (2009) 439–447. https://doi.org/10.3109/03630260903344176.
[341] E. a. A. Cordero, T.D. Veit, M. a. L. da Silva, S.M.C. Jacques, L.M.D.R. Silla, J. a. B. Chies, HLA-G polymorphism influences the susceptibility to HCV infection in sickle cell disease patients, Tissue Antigens 74 (2009) 308–313. https://doi.org/10.1111/j.1399-0039.2009.01331.x.
[342] Z. Chen, H. Luo, M.H. Steinberg, D.H.K. Chui, BCL11A represses HBG transcription in K562 cells, Blood Cells Mol Dis 42 (2009) 144–149. https://doi.org/10.1016/j.bcmd.2008.12.003.
[343] C. Chassanidis, A. Kalamaras, M. Phylactides, F. Pourfarzad, S. Likousi, V. Maroulis, M.N. Papadakis, N.K. Vamvakopoulos, V. Aleporou-Marinou, G.P. Patrinos, P. Kollia, The Hellenic type of nondeletional hereditary persistence of fetal hemoglobin results from a novel mutation (g.-109G>T) in the HBG2 gene promoter, Ann Hematol 88 (2009) 549–555. https://doi.org/10.1007/s00277-008-0643-0.
[344] C.-W. Chang, Y.-S. Lai, K.M. Pawlik, K. Liu, C.-W. Sun, C. Li, T.R. Schoeb, T.M. Townes, Polycistronic lentiviral vector for “hit and run” reprogramming of adult skin fibroblasts to induced pluripotent stem cells, Stem Cells 27 (2009) 1042–1049. https://doi.org/10.1002/stem.39.
[345] C.-C. So, Y.-Q. Song, S.T. Tsang, L.-F. Tang, A.Y. Chan, E.S. Ma, L.-C. Chan, The HBS1L-MYB intergenic region on chromosome 6q23 is a quantitative trait locus controlling fetal haemoglobin level in carriers of beta-thalassaemia, J Med Genet 45 (2008) 745–751. https://doi.org/10.1136/jmg.2008.060335.
[346] N. Patel, C.S. Gonsalves, P. Malik, V.K. Kalra, Placenta growth factor augments endothelin-1 and endothelin-B receptor expression via hypoxia-inducible factor-1 alpha, Blood 112 (2008) 856–865. https://doi.org/10.1182/blood-2007-12-130567.
[347] G. Parrinello, D. Torres, S. Paterna, P. Di Pasquale, G. Licata, Fever of unclear origin and cytopenia because of acute splenic sequestration in a young immunocompetent carrier of beta-globin mutation for Hb Valletta, Am J Med Sci 336 (2008) 508–511. https://doi.org/10.1097/MAJ.0b013e318162d13f.
[348] R. Martins, A. Morais, A. Dias, I. Soares, C. Rolão, J.L. Ducla-Soares, L. Braga, T. Seixas, B. Nunes, G. Olim, L. Romão, J. Lavinha, P. Faustino, Early modification of sickle cell disease clinical course by UDP-glucuronosyltransferase 1A1 gene promoter polymorphism, J Hum Genet 53 (2008) 524–528. https://doi.org/10.1007/s10038-008-0281-3.
[349] N. Maina, L. Zhong, X. Li, W. Zhao, Z. Han, D. Bischof, G. Aslanidi, S. Zolotukhin, K.A. Weigel-Van Aken, A.E. Rivers, W.B. Slayton, M.C. Yoder, A. Srivastava, Optimization of recombinant adeno-associated viral vectors for human beta-globin gene transfer and transgene expression, Hum Gene Ther 19 (2008) 365–375. https://doi.org/10.1089/hum.2007.173.
[350] N. Maina, Z. Han, X. Li, Z. Hu, L. Zhong, D. Bischof, K.A. Weigel-Van Aken, W.B. Slayton, M.C. Yoder, A. Srivastava, Recombinant self-complementary adeno-associated virus serotype vector-mediated hematopoietic stem cell transduction and lineage-restricted, long-term transgene expression in a murine serial bone marrow

transplantation model, Hum Gene Ther 19 (2008) 376–383. https://doi.org/10.1089/hum.2007.143.
[351] R. Mabaera, M.R. Greene, C.A. Richardson, S.J. Conine, C.D. Kozul, C.H. Lowrey, Neither DNA hypomethylation nor changes in the kinetics of erythroid differentiation explain 5-azacytidine’s ability to induce human fetal hemoglobin, Blood 111 (2008) 411–420. https://doi.org/10.1182/blood-2007-06-093948.
[352] C. Kumkhaek, J.G. Taylor, J. Zhu, C. Hoppe, G.J. Kato, G.P. Rodgers, Fetal haemoglobin response to hydroxycarbamide treatment and sar1a promoter polymorphisms in sickle cell anaemia, Br J Haematol 141 (2008) 254–259. https://doi.org/10.1111/j.1365-2141.2008.07045.x.
[353] P.W. Hargrove, S. Kepes, H. Hanawa, J.C. Obenauer, D. Pei, C. Cheng, J.T. Gray, G. Neale, D.A. Persons, Globin lentiviral vector insertions can perturb the expression of endogenous genes in beta-thalassemic hematopoietic cells, Mol Ther 16 (2008) 525–533. https://doi.org/10.1038/sj.mt.6300394.
[354] G.T. Gibney, C.I.M. Panhuysen, J.C.C. So, E.S.K. Ma, S.Y. Ha, C.K. Li, A.C.W. Lee, C.K. Li, H.L. Yuen, Y.L. Lau, D.M. Johnson, J.J. Farrell, A.B. Bisbee, L.A. Farrer, M.H. Steinberg, L.C. Chan, D.H.K. Chui, Variation and heritability of Hb F and F-cells among beta-thalassemia heterozygotes in Hong Kong, Am J Hematol 83 (2008) 458–464. https://doi.org/10.1002/ajh.21150.
[355] C.E. Eyler, T. Jackson, L.E. Elliott, L.M. De Castro, J. Jonassaint, A. Ashley-Koch, M.J. Telen, beta(2)-Adrenergic receptor and adenylate cyclase gene polymorphisms affect sickle red cell adhesion, Br J Haematol 141 (2008) 105–108. https://doi.org/10.1111/j.1365-2141.2008.07008.x.
[356] D.S. Darbari, R.H.N. van Schaik, E.V. Capparelli, S. Rana, R. McCarter, J. van den Anker, UGT2B7 promoter variant -840G>A contributes to the variability in hepatic clearance of morphine in patients with sickle cell disease, Am J Hematol 83 (2008) 200–202. https://doi.org/10.1002/ajh.21051.
[357] S.E. Cox, C.P. Doherty, S.H. Atkinson, C.V. Nweneka, A.J.C. Fulford, G. Sirugo, K.A. Rockett, D.P. Kwiatkowski, A.M. Prentice, Haptoglobin genotype, anaemia and malaria in Gambian children, Trop Med Int Health 13 (2008) 76–82. https://doi.org/10.1111/j.1365-3156.2007.01976.x.
[358] Z. Chen, H.-Y. Luo, R.K. Basran, T.-H. Hsu, D.W.H. Mang, L. Nuntakarn, C.G. Rosenfield, G.P. Patrinos, R.C. Hardison, M.H. Steinberg, D.H.K. Chui, A T-to-G transversion at nucleotide -567 upstream of HBG2 in a GATA-1 binding motif is associated with elevated hemoglobin F, Mol Cell Biol 28 (2008) 4386–4393. https://doi.org/10.1128/MCB.00071-08.
[359] S.L. Carpenter, S. Lieff, T.A. Howard, B. Eggleston, R.E. Ware, UGT1A1 promoter polymorphisms and the development of hyperbilirubinemia and gallbladder disease in children with sickle cell anemia, Am J Hematol 83 (2008) 800–803. https://doi.org/10.1002/ajh.21264.
[360] A. Buzina, M.Y.M. Lo, A. Moffett, A. Hotta, E. Fussner, R.R. Bharadwaj, P. Pasceri, J.V. Garcia-Martinez, D.P. Bazett-Jones, J. Ellis, Beta-globin LCR and intron elements cooperate and direct spatial reorganization for gene therapy, PLoS Genet 4 (2008) e1000051. https://doi.org/10.1371/journal.pgen.1000051.
[361] E.V. Adorno, J.P. Moura-Neto, I. Lyra, A. Zanette, L.F.O. Santos, M.O. Seixas, M.G. Reis, M.S. Goncalves, Sequence change in the HS2-LCR and Ggamma-globin gene promoter region of sickle cell anemia patients, Braz J Med Biol Res 41 (2008) 95–98. https://doi.org/10.1590/s0100-879x2008005000002.

[362] J. Zhu, B.T. Kren, C.W. Park, R. Bilgim, P.Y.-P. Wong, C.J. Steer, Erythroid-specific expression of beta-globin by the sleeping beauty transposon for Sickle cell disease, Biochemistry 46 (2007) 6844–6858. https://doi.org/10.1021/bi6024484.
[363] G.-H. Wei, G.-W. Zhao, W. Song, D.-L. Hao, X. Lv, D.-P. Liu, C.-C. Liang, Mechanisms of human gamma-globin transcriptional induction by apicidin involves p38 signaling to chromatin, Biochem Biophys Res Commun 363 (2007) 889–894. https://doi.org/10.1016/j.bbrc.2007.06.191.
[364] N. Vasavda, S. Menzel, S. Kondaveeti, E. Maytham, M. Awogbade, S. Bannister, J. Cunningham, A. Eichholz, Y. Daniel, I. Okpala, T. Fulford, S.L. Thein, The linear effects of alpha-thalassaemia, the UGT1A1 and HMOX1 polymorphisms on cholelithiasis in sickle cell disease, Br J Haematol 138 (2007) 263–270. https://doi.org/10.1111/j.1365-2141.2007.06643.x.
[365] L. Valenti, E.A. Pulixi, P. Arosio, L. Cremonesi, G. Biasiotto, P. Dongiovanni, M. Maggioni, S. Fargion, A.L. Fracanzani, Relative contribution of iron genes, dysmetabolism and hepatitis C virus (HCV) in the pathogenesis of altered iron regulation in HCV chronic hepatitis, Haematologica 92 (2007) 1037–1042. https://doi.org/10.3324/haematol.11281.
[366] A. Morgado, I. Picanço, S. Gomes, A. Miranda, M. Coucelo, F. Seuanes, M.T. Seixas, L. Romão, P. Faustino, Mutational spectrum of delta-globin gene in the Portuguese population, Eur J Haematol 79 (2007) 422–428. https://doi.org/10.1111/j.1600-0609.2007.00949.x.
[367] D. Manwani, M. Galdass, J.J. Bieker, Altered regulation of beta-like globin genes by a redesigned erythroid transcription factor, Exp Hematol 35 (2007) 39–47. https://doi.org/10.1016/j.exphem.2006.09.004.
[368] Q. Ma, K. Abel, O. Sripichai, J. Whitacre, V. Angkachatchai, W. Makarasara, P. Winichagoon, S. Fucharoen, A. Braun, L.A. Farrer, Beta-globin gene cluster polymorphisms are strongly associated with severity of HbE/beta(0)-thalassemia, Clin Genet 72 (2007) 497–505. https://doi.org/10.1111/j.1399-0004.2007.00897.x.
[369] D. Lavelle, J. Chin, K. Vaitkus, S. Redkar, P. Phiasivongsa, C. Tang, R. Will, M. Hankewych, B. Roxas, M. Singh, Y. Saunthararajah, J. Desimone, Oral decitabine reactivates expression of the methylated gamma-globin gene in Papio anubis, Am J Hematol 82 (2007) 981–985. https://doi.org/10.1002/ajh.21020.
[370] C. Hoppe, W. Klitz, K. D'Harlingue, S. Cheng, M. Grow, L. Steiner, J. Noble, R. Adams, L. Styles, Stroke Prevention Trial in Sickle Cell Anemia (STOP) Investigators, Confirmation of an association between the TNF(-308) promoter polymorphism and stroke risk in children with sickle cell anemia, Stroke 38 (2007) 2241–2246. https://doi.org/10.1161/STROKEAHA.107.483115.
[371] O.P. Dossou-Yovo, C. Lapoumeroulie, M. Hauchecorne, I. Zaccaria, R. Ducrocq, R. Krishnamoorthy, M.C. Rahimy, J. Elion, Variants of the mannose-binding lectin gene in the Benin population: heterozygosity for the p.G57E allele may confer a selective advantage, Hum Biol 79 (2007) 687–697. https://doi.org/10.1353/hub.2008.0012.
[372] S.E. Cox, C. Doherty, S.H. Atkinson, C.V. Nweneka, A.J.C. Fulford, H. Ghattas, K.A. Rockett, D.P. Kwiatkowski, A.M. Prentice, Haplotype association between haptoglobin (Hp2) and Hp promoter SNP (A-61C) may explain previous controversy of haptoglobin and malaria protection, PLoS One 2 (2007) e362. https://doi.org/10.1371/journal.pone.0000362.
[373] O. Cohen-Barak, D.T. Erickson, M.S. Badowski, D.A. Fuchs, C.L. Klassen, D.T. Harris, M.H. Brilliant, Stem cell transplantation demonstrates that Sox6 represses epsilon y globin expression in definitive erythropoiesis of adult mice, Exp Hematol 35 (2007) 358–367. https://doi.org/10.1016/j.exphem.2006.11.009.

[374] X.-W. Chen, Q.-H. Mo, Q. Li, R. Zeng, X.-M. Xu, A novel mutation of -73(A-->T) in the CCAAT box of the beta-globin gene identified in a patient with the mild beta-thalassemia intermedia, Ann Hematol 86 (2007) 653–657. https://doi.org/10.1007/s00277-007-0312-8.

[375] Z. Yi, O. Cohen-Barak, N. Hagiwara, P.D. Kingsley, D.A. Fuchs, D.T. Erickson, E.M. Epner, J. Palis, M.H. Brilliant, Sox6 directly silences epsilon globin expression in definitive erythropoiesis, PLoS Genet 2 (2006) e14. https://doi.org/10.1371/journal.pgen.0020014.

[376] S.P. Singh, S. Gupta, Molecular pathogenesis and clinical variability of homozygous beta0-thalassemia in populations of Jammu region of J&K state (India), Hematology 11 (2006) 271–275. https://doi.org/10.1080/10245330600921956.

[377] D. Lavelle, K. Vaitkus, M. Hankewych, M. Singh, J. DeSimone, Effect of 5-aza-2'-deoxycytidine (Dacogen) on covalent histone modifications of chromatin associated with the epsilon-, gamma-, and beta-globin promoters in Papio anubis, Exp Hematol 34 (2006) 339–347. https://doi.org/10.1016/j.exphem.2005.12.010.

[378] M.I. Lai, J. Jiang, N. Silver, S. Best, S. Menzel, A. Mijovic, S. Colella, J. Ragoussis, C. Garner, M.J. Weiss, S.L. Thein, Alpha-haemoglobin stabilising protein is a quantitative trait gene that modifies the phenotype of beta-thalassaemia, Br J Haematol 133 (2006) 675–682. https://doi.org/10.1111/j.1365-2141.2006.06075.x.

[379] K.S. Kim, V. Rajagopal, C. Gonsalves, C. Johnson, V.K. Kalra, A novel role of hypoxia-inducible factor in cobalt chloride- and hypoxia-mediated expression of IL-8 chemokine in human endothelial cells, J Immunol 177 (2006) 7211–7224. https://doi.org/10.4049/jimmunol.177.10.7211.

[380] C. Hémar, O. Nibourel, P. Maboudou, C. Méreau-Richard, C. Badens, J. Rousseaux, C. Rose, [Beta(o)/beta(o) thalassemia with a mild phenotype], Ann Biol Clin (Paris) 64 (2006) 341–345.

[381] D.M. Dykxhoorn, L.D. Schlehuber, I.M. London, J. Lieberman, Determinants of specific RNA interference-mediated silencing of human beta-globin alleles differing by a single nucleotide polymorphism, Proc Natl Acad Sci U S A 103 (2006) 5953–5958. https://doi.org/10.1073/pnas.0601309103.

[382] V. Chaar, L. Kéclard, M. Etienne-Julan, J.P. Diara, J. Elion, R. Krishnamoorthy, M. Romana, UGT1A1 polymorphism outweighs the modest effect of deletional (-3.7 kb) alpha-thalassemia on cholelithogenesis in sickle cell anemia, Am J Hematol 81 (2006) 377–379. https://doi.org/10.1002/ajh.20574.

[383] G.A. Awandare, C. Ouma, C.C. Keller, T. Were, R. Otieno, Y. Ouma, G.C. Davenport, J.B. Hittner, J.M. Ong'echa, R. Ferrell, D.J. Perkins, A macrophage migration inhibitory factor promoter polymorphism is associated with high-density parasitemia in children with malaria, Genes Immun 7 (2006) 568–575. https://doi.org/10.1038/sj.gene.6364332.

[384] W. Yin, B.T. Kren, C.J. Steer, Site-specific base changes in the coding or promoter region of the human beta- and gamma-globin genes by single-stranded oligonucleotides, Biochem J 390 (2005) 253–261. https://doi.org/10.1042/BJ20050045.

[385] M. Vilatoba, C. Eckstein, G. Bilbao, C.A. Smyth, S. Jenkins, J.A. Thompson, D.E. Eckhoff, J.L. Contreras, Sodium 4-phenylbutyrate protects against liver ischemia reperfusion injury by inhibition of endoplasmic reticulum-stress mediated apoptosis, Surgery 138 (2005) 342–351. https://doi.org/10.1016/j.surg.2005.04.019.

[386] A.A. Sarkar, C. Mukhopadhyay, S. Chandra, S. Banerjee, M.K. Das, U.B. Dasgupta, Co-inheritance of the Hb Sun Prairie mutation with a point mutation at 5'-UTR in the

eastern Indian population, Br J Haematol 129 (2005) 282–286. https://doi.org/10.1111/j.1365-2141.2005.05451.x.
[387] F. Kutlar, D. Mirmow, M. Glendenning, L. Holley, A. Kutlar, Postmortem molecular diagnosis of sickle beta thalassaemia, J Clin Pathol 58 (2005) 548–549. https://doi.org/10.1136/jcp.2004.018127.
[388] J. Johnson, R. Hunter, R. McElveen, X.H. Qian, B.S. Baliga, B.S. Pace, Fetal hemoglobin induction by the histone deacetylase inhibitor, scriptaid, Cell Mol Biol (Noisy-Le-Grand) 51 (2005) 229–238.
[389] M.M. Heeney, Risk of cholelithiasis in sickle cell anemia, Haematologica 90 (2005) 147.
[390] E.V. Haverfield, C.A. McKenzie, T. Forrester, N. Bouzekri, R. Harding, G. Serjeant, T. Walker, T.E.A. Peto, R. Ward, D.J. Weatherall, UGT1A1 variation and gallstone formation in sickle cell disease, Blood 105 (2005) 968–972. https://doi.org/10.1182/blood-2004-02-0521.
[391] T. Gräslund, X. Li, L. Magnenat, M. Popkov, C.F. Barbas, Exploring strategies for the design of artificial transcription factors: targeting sites proximal to known regulatory regions for the induction of gamma-globin expression and the treatment of sickle cell disease, J Biol Chem 280 (2005) 3707–3714. https://doi.org/10.1074/jbc.M406809200.
[392] G. Garewal, R. Das, J. Ahluwalia, R.K. Marwaha, S. Varma, Nucleotide -88 (C-T) promoter mutation is a common beta-thalassemia mutation in the Jat Sikhs of Punjab, India, Am J Hematol 79 (2005) 252–256. https://doi.org/10.1002/ajh.20445.
[393] S.C. Crable, S.M. Hammond, R. Papes, R.K. Rettig, G.-P. Zhou, P.G. Gallagher, C.H. Joiner, K.P. Anderson, Multiple isoforms of the KC1 cotransporter are expressed in sickle and normal erythroid cells, Exp Hematol 33 (2005) 624–631. https://doi.org/10.1016/j.exphem.2005.02.006.
[394] V. Chaar, L. Kéclard, J.P. Diara, C. Leturdu, J. Elion, R. Krishnamoorthy, J. Clayton, M. Romana, Association of UGT1A1 polymorphism with prevalence and age at onset of cholelithiasis in sickle cell anemia, Haematologica 90 (2005) 188–199.
[395] J.C. Barton, P.L. Lee, L.F. Bertoli, E. Beutler, Iron overload in an African American woman with SS hemoglobinopathy and a promoter mutation in the X-linked erythroid-specific 5-aminolevulinate synthase (ALAS2) gene, Blood Cells Mol Dis 34 (2005) 226–228. https://doi.org/10.1016/j.bcmd.2005.01.001.
[396] S.K. Ayesh, W.A. Al-Sharef, S.M. Nassar, N.A. Thawabteh, B.Y. Abu-Libdeh, Prenatal diagnosis of beta-thalassemia in the West Bank and Gaza, Saudi Med J 26 (2005) 1771–1776.
[397] T. Ammosova, R. Berro, F. Kashanchi, S. Nekhai, RNA interference directed to CDK2 inhibits HIV-1 transcription, Virology 341 (2005) 171–178. https://doi.org/10.1016/j.virol.2005.06.041.
[398] A. Adekile, F. Kutlar, K. McKie, A. Addington, D. Elam, L. Holley, B. Clair, A. Kutlar, The influence of uridine diphosphate glucuronosyl transferase 1A promoter polymorphisms, beta-globin gene haplotype, co-inherited alpha-thalassemia trait and Hb F on steady-state serum bilirubin levels in sickle cell anemia, Eur J Haematol 75 (2005) 150–155. https://doi.org/10.1111/j.1600-0609.2005.00477.x.
[399] G.-P. Zhou, C. Wong, R. Su, S.C. Crable, K.P. Anderson, P.G. Gallagher, Human potassium chloride cotransporter 1 (SLC12A4) promoter is regulated by AP-2 and contains a functional downstream promoter element, Blood 103 (2004) 4302–4309. https://doi.org/10.1182/blood-2003-01-0107.
[400] J. Vadolas, H. Wardan, M. Orford, R. Williamson, P.A. Ioannou, Cellular genomic reporter assays for screening and evaluation of inducers of fetal hemoglobin, Hum Mol Genet 13 (2004) 223–233. https://doi.org/10.1093/hmg/ddh023.

[401] X. Qi, T. Hosoi, Y. Okuma, M. Kaneko, Y. Nomura, Sodium 4-phenylbutyrate protects against cerebral ischemic injury, Mol Pharmacol 66 (2004) 899–908. https://doi.org/10.1124/mol.104.001339.
[402] S. Parikh, G. Dorsey, P.J. Rosenthal, Host polymorphisms and the incidence of malaria in Ugandan children, Am J Trop Med Hyg 71 (2004) 750–753.
[403] I.-H. Oh, M.E. Fabry, R.K. Humphries, R. Pawliuk, P. Leboulch, R. Hoffman, R.L. Nagel, C. Eaves, Expression of an anti-sickling beta-globin in human erythroblasts derived from retrovirally transduced primitive normal and sickle cell disease hematopoietic cells, Exp Hematol 32 (2004) 461–469. https://doi.org/10.1016/j.exphem.2004.02.001.
[404] S.F. Ofori-Acquah, M.R.A. Lalloz, G. Serjeant, D.M. Layton, Dominant influence of gamma-globin promoter polymorphisms on fetal haemoglobin expression in sickle cell disease, Cell Mol Biol (Noisy-Le-Grand) 50 (2004) 35–42.
[405] A. Nadkarni, T. Sakaguchi, A. Gorakshakar, S. Phanasgaonkar, R. Kiyama, R. Colah, D. Mohanty, An interplay of alleviating mutations in the clinical phenotype of beta-thalassaemia intermedia, Clin Lab Haematol 26 (2004) 419–422. https://doi.org/10.1111/j.1365-2257.2004.00638.x.
[406] P. Moi, V. Faà, M.G. Marini, I. Asunis, G. Ibba, A. Cao, M.C. Rosatelli, A novel silent beta-thalassemia mutation in the distal CACCC box affects the binding and responsiveness to EKLF, Br J Haematol 126 (2004) 881–884. https://doi.org/10.1111/j.1365-2141.2004.05146.x.
[407] H. Hanawa, P.W. Hargrove, S. Kepes, D.K. Srivastava, A.W. Nienhuis, D.A. Persons, Extended beta-globin locus control region elements promote consistent therapeutic expression of a gamma-globin lentiviral vector in murine beta-thalassemia, Blood 104 (2004) 2281–2290. https://doi.org/10.1182/blood-2004-03-0863.
[408] P.A. Zalloua, E. Aoun, S. Koussa, W.S.Z. Asfahani, A. Taher, The codons 8/9 (+G) mutation found for the first time in the Lebanese population, Hemoglobin 27 (2003) 1–5. https://doi.org/10.1081/hem-120018430.
[409] V. Viprakasit, A.M.J. Kidd, H. Ayyub, S. Horsley, J. Hughes, D.R. Higgs, De novo deletion within the telomeric region flanking the human alpha globin locus as a cause of alpha thalassaemia, Br J Haematol 120 (2003) 867–875. https://doi.org/10.1046/j.1365-2141.2003.04197.x.
[410] S. Sirichotiyakul, R. Saetung, T. Sanguansermsri, Analysis of beta-thalassemia mutations in northern Thailand using an automated fluorescence DNA sequencing technique, Hemoglobin 27 (2003) 89–95. https://doi.org/10.1081/hem-120021541.
[411] Y. Saunthararajah, C.A. Hillery, D. Lavelle, R. Molokie, L. Dorn, L. Bressler, S. Gavazova, Y.-H. Chen, R. Hoffman, J. DeSimone, Effects of 5-aza-2’-deoxycytidine on fetal hemoglobin levels, red cell adhesion, and hematopoietic differentiation in patients with sickle cell disease, Blood 102 (2003) 3865–3870. https://doi.org/10.1182/blood-2003-05-1738.
[412] D.A. Persons, P.W. Hargrove, E.R. Allay, H. Hanawa, A.W. Nienhuis, The degree of phenotypic correction of murine beta -thalassemia intermedia following lentiviral-mediated transfer of a human gamma-globin gene is influenced by chromosomal position effects and vector copy number, Blood 101 (2003) 2175–2183. https://doi.org/10.1182/blood-2002-07-2211.
[413] M. Loyevsky, F. Mompoint, E. Yikilmaz, S.F. Altschul, T. Madden, J.C. Wootton, J. Kurantsin-Mills, O.O. Kassim, V.R. Gordeuk, T.A. Rouault, Expression of a recombinant IRP-like Plasmodium falciparum protein that specifically binds putative plasmodial IREs, Mol Biochem Parasitol 126 (2003) 231–238. https://doi.org/10.1016/s0166-6851(02)00278-5.

[414] W. Li, X. Lao, S. Jai, F. Liang, Q. Mo, J. Ma, X. Xu, [A rare transcription mutation (-90 C-->T) in a Chinese family with beta-thalassemia], Zhonghua Yi Xue Yi Chuan Xue Za Zhi 20 (2003) 468–470.
[415] V. Kalotychou, K. Antonatou, R. Tzanetea, E. Terpos, D. Loukopoulos, Y. Rombos, Analysis of the A(TA)(n)TAA configuration in the promoter region of the UGT1 A1 gene in Greek patients with thalassemia intermedia and sickle cell disease, Blood Cells Mol Dis 31 (2003) 38–42. https://doi.org/10.1016/s1079-9796(03)00118-9.
[416] X.-J. Ji, D. Liu, D.-D. Xu, L. Li, C. Liang, Effect of fetal hemoglobin-stimulating medicines on the interaction of DNA and protein of important erythroid regulatory elements, Biochem Cell Biol 81 (2003) 297–305. https://doi.org/10.1139/o03-058.
[417] M.M. Heeney, T.A. Howard, S.A. Zimmerman, R.E. Ware, UGT1A promoter polymorphisms influence bilirubin response to hydroxyurea therapy in sickle cell anemia, J Lab Clin Med 141 (2003) 279–282. https://doi.org/10.1067/mlc.2003.28.
[418] J.D. Haley, D.E. Smith, J. Schwedes, R. Brennan, C. Pearce, C. Moore, F. Wang, F. Petti, F. Grosveld, S.M. Jane, C.T. Noguchi, A.N. Schechter, Identification and characterization of mechanistically distinct inducers of gamma-globin transcription, Biochem Pharmacol 66 (2003) 1755–1768. https://doi.org/10.1016/s0006-2952(03)00542-2.
[419] K.Y. Fertrin, M.B. Melo, A.M. Assis, S.T.O. Saad, F.F. Costa, UDP-glucuronosyltransferase 1 gene promoter polymorphism is associated with increased serum bilirubin levels and cholecystectomy in patients with sickle cell anemia, Clin Genet 64 (2003) 160–162. https://doi.org/10.1034/j.1399-0004.2003.00113.x.
[420] C. Borgna-Pignatti, F. Rigon, L. Merlo, R. Chakrok, R. Micciolo, L. Perseu, R. Galanello, Thalassemia minor, the Gilbert mutation, and the risk of gallstones, Haematologica 88 (2003) 1106–1109.
[421] T. Ammosova, M. Jerebtsova, M. Beullens, Y. Voloshin, P.E. Ray, A. Kumar, M. Bollen, S. Nekhai, Nuclear protein phosphatase-1 regulates HIV-1 transcription, J Biol Chem 278 (2003) 32189–32194. https://doi.org/10.1074/jbc.M300521200.
[422] J.G. Taylor, D.C. Tang, S.A. Savage, S.F. Leitman, S.I. Heller, G.R. Serjeant, G.P. Rodgers, S.J. Chanock, Variants in the VCAM1 gene and risk for symptomatic stroke in sickle cell disease, Blood 100 (2002) 4303–4309. https://doi.org/10.1182/blood-2001-12-0306.
[423] O. Tanabe, F. Katsuoka, A.D. Campbell, W. Song, M. Yamamoto, K. Tanimoto, J.D. Engel, An embryonic/fetal beta-type globin gene repressor contains a nuclear receptor TR2/TR4 heterodimer, EMBO J 21 (2002) 3434–3442. https://doi.org/10.1093/emboj/cdf340.
[424] M.N. Papadakis, G.P. Patrinos, P. Tsaftaridis, A. Loutradi-Anagnostou, A comparative study of Greek nondeletional hereditary persistence of fetal hemoglobin and beta-thalassemia compound heterozygotes, J Mol Med (Berl) 80 (2002) 243–247. https://doi.org/10.1007/s00109-001-0312-4.
[425] R. Kukreti, D. Dash, V.K. E, S. Chakravarty, S.K. Das, M. De, G. Talukder, Spectrum of beta-thalassemia mutations and their association with allelic sequence polymorphisms at the beta-globin gene cluster in an Eastern Indian population, Am J Hematol 70 (2002) 269–277. https://doi.org/10.1002/ajh.10117.
[426] R. Galanello, S. Barella, S. Satta, L. Maccioni, C. Pintor, A. Cao, Homozygosity for nondeletion delta-beta(0) thalassemia resulting in a silent clinical phenotype, Blood 100 (2002) 1913–1914.
[427] D.W. Emery, E. Yannaki, J. Tubb, T. Nishino, Q. Li, G. Stamatoyannopoulos, Development of virus vectors for gene therapy of beta chain hemoglobinopathies:

flanking with a chromatin insulator reduces gamma-globin gene silencing in vivo, Blood 100 (2002) 2012–2019. https://doi.org/10.1182/blood-2002-01-0219.
[428] M. De Angioletti, G. Lacerra, C. Gaudiano, G. Mastrolonardo, L. Pagano, L. Mastrullo, S. Masciandaro, C. Carestia, Epidemiology of the delta globin alleles in southern Italy shows complex molecular, genetic, and phenotypic features, Hum Mutat 20 (2002) 358–367. https://doi.org/10.1002/humu.10132.
[429] M.B. Chase, S. Fu, S.B. Haga, G. Davenport, H. Stevenson, K. Do, D. Morgan, A.L. Mah, P.E. Berg, BP1, a homeodomain-containing isoform of DLX4, represses the beta-globin gene, Mol Cell Biol 22 (2002) 2505–2514. https://doi.org/10.1128/MCB.22.8.2505-2514.2002.
[430] L. Castilho, M. Rios, J. Pellegrino, S. T O Saad, F. F Costa, Blood group genotyping facilitates transfusion of beta-thalassemia patients, J Clin Lab Anal 16 (2002) 216–220. https://doi.org/10.1002/jcla.10044.
[431] M. Yavarian, C.L. Harteveld, D. Batelaan, L.F. Bernini, P.C. Giordano, Molecular spectrum of beta-thalassemia in the Iranian Province of Hormozgan, Hemoglobin 25 (2001) 35–43. https://doi.org/10.1081/hem-100103068.
[432] E.M. Wood, I. Woolley, Re: Duffy phenotype does not influence the clinical severity of sickle cell disease, Clin Immunol 98 (2001) 383–384. https://doi.org/10.1006/clim.2000.4990.
[433] J. Pellegrino, L. Castilho, M. Rios, C.A. De Souza, Blood group genotyping in a population of highly diverse ancestry, J Clin Lab Anal 15 (2001) 8–13. https://doi.org/10.1002/1098-2825(2001)15:1&%2523x0003c;8::AID-JCLA2&%2523x0003e;3.0.CO;2-8.
[434] R.G. Passon, T.A. Howard, S.A. Zimmerman, W.H. Schultz, R.E. Ware, Influence of bilirubin uridine diphosphate-glucuronosyltransferase 1A promoter polymorphisms on serum bilirubin levels and cholelithiasis in children with sickle cell anemia, J Pediatr Hematol Oncol 23 (2001) 448–451. https://doi.org/10.1097/00043426-200110000-00011.
[435] R. Oner, C. Oner, E. Birben, M. Sözen, F. Gümrük, A. Gürgey, C. Altay, beta-Thalassaemia intermedia in a Turkish girl: homozygosity for G-->A substitution at +22 relative to the beta-globin cap site, Br J Haematol 115 (2001) 90–94. https://doi.org/10.1046/j.1365-2141.2001.03071.x.
[436] S.F. Ofori-Acquah, M.R. Lalloz, D.M. Layton, Nucleotide variation regulates the level of enhancement by hypersensitive site 2 of the beta-globin locus control region, Blood Cells Mol Dis 27 (2001) 803–811. https://doi.org/10.1006/bcmd.2001.0449.
[437] A. Nadkarni, A.C. Gorakshakar, C.Y. Lu, R. Krishnamoorthy, K. Ghosh, R. Colah, D. Mohanty, Molecular pathogenesis and clinical variability of beta-thalassemia syndromes among Indians, Am J Hematol 68 (2001) 75–80. https://doi.org/10.1002/ajh.1156.
[438] Y.P. Chang, R. Littera, R. Garau, K.D. Smith, G.J. Dover, S. Iannelli, E. Cacace, L. Contu, The role of heterocellular hereditary persistence of fetal haemoglobin in beta(0)-thalassaemia intermedia, Br J Haematol 114 (2001) 899–906. https://doi.org/10.1046/j.1365-2141.2001.03042.x.
[439] E. Birben, C. Oner, R. Oner, H. Mergen, A. Yesilipek, F. Gümrük, A. Gürgey, C. Altay, Severe beta-thalassemia in frameshift codon 6 (-A) homozygotes: effects of haplotype on phenotype, Hemoglobin 25 (2001) 441–445. https://doi.org/10.1081/hem-100107884.
[440] M. Qatanani, A. Taher, S. Koussa, R. Naaman, C. Fisher, M. Rugless, J. Old, L. Zahed, Beta-thalassaemia intermedia in Lebanon, Eur J Haematol 64 (2000) 237–244. https://doi.org/10.1034/j.1600-0609.2000.90087.x.

[441] C.K. Mukhopadhyay, B. Mazumder, P.L. Fox, Role of hypoxia-inducible factor-1 in transcriptional activation of ceruloplasmin by iron deficiency, J Biol Chem 275 (2000) 21048–21054. https://doi.org/10.1074/jbc.M000636200.
[442] F. Migot-Nabias, L.E. Mombo, A.J. Luty, B. Dubois, R. Nabias, C. Bisseye, P. Millet, C.Y. Lu, P. Deloron, Human genetic factors related to susceptibility to mild malaria in Gabon, Genes Immun 1 (2000) 435–441. https://doi.org/10.1038/sj.gene.6363703.
[443] H.Y. Lung, I.S. Meeus, R.S. Weinberg, G.F. Atweh, In vivo silencing of the human gamma-globin gene in murine erythroid cells following retroviral transduction, Blood Cells Mol Dis 26 (2000) 613–619. https://doi.org/10.1006/bcmd.2000.0343.
[444] M. Gabbianelli, U. Testa, A. Massa, E. Pelosi, N.M. Sposi, R. Riccioni, L. Luchetti, C. Peschle, Hemoglobin switching in unicellular erythroid culture of sibling erythroid burst-forming units: kit ligand induces a dose-dependent fetal hemoglobin reactivation potentiated by sodium butyrate, Blood 95 (2000) 3555–3561.
[445] M.A. el-Hazmi, A.S. Warsy, Xmn I polymorphic site in Yemeni sickle cell disease patients, J Trop Pediatr 46 (2000) 25–29. https://doi.org/10.1093/tropej/46.1.25.
[446] M.C. Bento, M.L. Ribeiro, E. Cunha, P. Gonçalves, G. Martin-Nuñez, G. Tamagnini, b-thalassemia intermedia resulting from compound heterozigosity for an IVSI-1 (G-A) and a silent 5' UTR +33 (C-G) mutations, Haematologica 85 (2000) 443–444.
[447] S. Zertal-Zidani, T. Merghoub, R. Ducrocq, N. Gerard, D. Satta, R. Krishnamoorthy, A novel C-->A transversion within the distal CCAAT motif of the Ggamma-globin gene in the Algerian Ggammabeta+-hereditary persistence of fetal hemoglobin, Hemoglobin 23 (1999) 159–169. https://doi.org/10.3109/03630269908996160.
[448] H.A. Stirnadel, M. Stöckle, I. Felger, T. Smith, M. Tanner, H.P. Beck, Malaria infection and morbidity in infants in relation to genetic polymorphisms in Tanzania, Trop Med Int Health 4 (1999) 187–193. https://doi.org/10.1046/j.1365-3156.1999.43381.x.
[449] S. Samakoglu, S. Philipsen, F. Grosveld, G. Lüleci, H. Bagci, Nucleotide changes in the gamma-globin promoter and the (AT)xNy(AT)z polymorphic sequence of beta LCRHS-2 region associated with altered levels of HbF, Eur J Hum Genet 7 (1999) 345–356. https://doi.org/10.1038/sj.ejhg.5200284.
[450] S.F. Ofori-Acquah, M.R. Lalloz, D.M. Layton, Localisation of cis regulatory elements at the beta-globin locus: analysis of hybrid haplotype chromosomes, Biochem Biophys Res Commun 254 (1999) 181–187. https://doi.org/10.1006/bbrc.1998.9901.
[451] M.G. Neonato, C.Y. Lu, M. Guilloud-Bataille, C. Lapouméroulie, H. Nabeel-Jassim, D. Dabit, R. Girot, R. Krishnamoorthy, J. Feingold, C. Besmond, J. Elion, Genetic polymorphism of the mannose-binding protein gene in children with sickle cell disease: identification of three new variant alleles and relationship to infections, Eur J Hum Genet 7 (1999) 679–686. https://doi.org/10.1038/sj.ejhg.5200360.
[452] E. Maragoudaki, E. Kanavakis, J. Traeger-Synodinos, C. Vrettou, M. Tzetis, A. Metaxotou-Mavrommati, C. Kattamis, Molecular, haematological and clinical studies of the -101 C --> T substitution of the beta-globin gene promoter in 25 beta-thalassaemia intermedia patients and 45 heterozygotes, Br J Haematol 107 (1999) 699–706. https://doi.org/10.1046/j.1365-2141.1999.01788.x.
[453] Q. Li, D.W. Emery, M. Fernandez, H. Han, G. Stamatoyannopoulos, Development of viral vectors for gene therapy of beta-chain hemoglobinopathies: optimization of a gamma-globin gene expression cassette, Blood 93 (1999) 2208–2216.
[454] C. Kurpad, P. Mukherjee, X.S. Wang, S. Ponnazhagan, L. Li, M.C. Yoder, A. Srivastava, Adeno-associated virus 2-mediated transduction and erythroid lineage-restricted expression from parvovirus B19p6 promoter in primary human

hematopoietic progenitor cells, J Hematother Stem Cell Res 8 (1999) 585–592. https://doi.org/10.1089/152581699319740.
[455] G.V. Dedoussis, K. Sinopoulou, M. Gyparaki, A. Loutradis, Fetal hemoglobin expression in the compound heterozygous state for -117 (G-->A) Agamma HPFH and IVSII-745 (C-->G) beta+ thalassemia: a case study, Am J Hematol 61 (1999) 139–143. https://doi.org/10.1002/(sici)1096-8652(199906)61:2%253C139::aid-ajh12%253E3.0.co;2-7.
[456] J.B. Clegg, D.J. Weatherall, Thalassemia and malaria: new insights into an old problem, Proc Assoc Am Physicians 111 (1999) 278–282. https://doi.org/10.1046/j.1525-1381.1999.99235.x.
[457] F. Ataulfo Gonzalez, P. Ropero, J. Sánchez, C. Rosatellí, R. Galanello, A. Villegas, C-->T mutation at -158 G gamma HPFH associated with 4 bp deletion (-225-222) in the promoter region of the A gamma gene in homozygous beta 0 39 nonsense thalassemia, Haematologica 84 (1999) 90–92.
[458] R. Alami, J.G. Gilman, Y.Q. Feng, A. Marmorato, I. Rochlin, S.M. Suzuka, M.E. Fabry, R.L. Nagel, E.E. Bouhassira, Anti-beta s-ribozyme reduces beta s mRNA levels in transgenic mice: potential application to the gene therapy of sickle cell anemia, Blood Cells Mol Dis 25 (1999) 110–119. https://doi.org/10.1006/bcmd.1999.0235.
[459] G.O. Tadmouri, L. Yüksel, A.N. Başak, HbS/beta(del)-thalassemia associated with high levels of hemoglobins A2 and F in a Turkish family, Am J Hematol 59 (1998) 83–86. https://doi.org/10.1002/(sici)1096-8652(199809)59:1%253C83::aid-ajh16%253E3.0.co;2-2.
[460] N.S. Smetanina, L.H. Gu, T.H. Huisman, Comparison of the relative quantities of gamma-mRNAs and fetal hemoglobin in SS patients with different haplotypes, Acta Haematol 100 (1998) 4–8. https://doi.org/10.1159/000040853.
[461] L.A. Michaels, K. Ohene-Frempong, H. Zhao, S.D. Douglas, Serum levels of substance P are elevated in patients with sickle cell disease and increase further during vaso-occlusive crisis, Blood 92 (1998) 3148–3151.
[462] E. Maragoudaki, C. Vrettou, E. Kanavakis, J. Traeger-Synodinos, A. Metaxotou-Mavrommati, C. Kattamis, Molecular, haematological and clinical studies of a silent beta-gene C-->G mutation at 6 bp 3’ to the termination codon (+1480 C-->G) in twelve Greek families, Br J Haematol 103 (1998) 45–51. https://doi.org/10.1046/j.1365-2141.1998.00966.x.
[463] T. Ikuta, Y.W. Kan, P.S. Swerdlow, D.V. Faller, S.P. Perrine, Alterations in protein-DNA interactions in the gamma-globin gene promoter in response to butyrate therapy, Blood 92 (1998) 2924–2933.
[464] M. Fichera, C. Romano, L. Castiglia, P. Failla, C. Ruberto, S. Amata, D. Greco, C. Cardoso, M. Fontés, A. Ragusa, New mutations in XNP/ATR-X gene: a further contribution to genotype/phenotype relationship in ATR/X syndrome. Mutations in brief no. 176. Online, Hum Mutat 12 (1998) 214.
[465] S. el-Kalla, E. Baysal, Genotype-phenotype correlation of sickle cell disease in the United Arab Emirates, Pediatr Hematol Oncol 15 (1998) 237–242. https://doi.org/10.3109/08880019809028790.
[466] D.C. Tang, D. Ebb, R.C. Hardison, G.P. Rodgers, Restoration of the CCAAT box or insertion of the CACCC motif activates [corrected] delta-globin gene expression, Blood 90 (1997) 421–427.
[467] N.S. Smetanina, L.H. Gu, G. Schilirò, A. Di Cataldo, R. Testa, Z. Jakovlevska, G.D. Efremov, T.H. Huisman, Relative levels of alpha-, beta-, and gamma-mRNA from patients with severe and intermediate beta-thalassemia major, Acta Haematol 97 (1997) 205–210. https://doi.org/10.1159/000203684.

[468] M. Papadakis, E. Papapanagiotou, A. Loutradi-Anagnostou, Scanning method to identify the molecular heterogeneity of delta-globin gene especially in delta-thalassemias: detection of three novel substitutions in the promoter region of the gene, Hum Mutat 9 (1997) 465–472. https://doi.org/10.1002/(SICI)1098-1004(1997)9:5%253C465::AID-HUMU14%253E3.0.CO;2-0.
[469] S. el-Kalla, A.R. Mathews, A significant beta-thalassemia heterogeneity in the United Arab Emirates, Hemoglobin 21 (1997) 237–247. https://doi.org/10.3109/03630269708997384.
[470] I. Bianco, M.P. Cappabianca, E. Foglietta, M. Lerone, G. Deidda, L. Morlupi, P. Grisanti, D. Ponzini, S. Rinaldi, B. Graziani, Silent thalassemias: genotypes and phenotypes, Haematologica 82 (1997) 269–280.
[471] S.K. Ballas, S.P. Cai, T. Gabuzda, F.F. Chehab, Molecular basis of asymptomatic beta-thalassemia major in an African American individual, Am J Med Genet 69 (1997) 196–199.
[472] S. Ren, B.Y. Wong, J. Li, X.N. Luo, P.M. Wong, G.F. Atweh, Production of genetically stable high-titer retroviral vectors that carry a human gamma-globin gene under the control of the alpha-globin locus control region, Blood 87 (1996) 2518–2524.
[473] S. Ohi, B.C. Kim, Synthesis of human globin polypeptides mediated by recombinant adeno-associated virus vectors, J Pharm Sci 85 (1996) 274–281. https://doi.org/10.1021/js950209o.
[474] J.C. Morgan, D.F. Scott, K.D. Lanclos, Two mutations in the locus control region hypersensitivity site-2 (5’ HS-2) of haplotype 19 beta s chromosomes alter binding of trans-acting factors, Am J Hematol 51 (1996) 12–18. https://doi.org/10.1002/(SICI)1096-8652(199601)51:1%253C12::AID-AJH3%253E3.0.CO;2-A.
[475] W.R. Hudgins, E. Fibach, S. Safaya, R.F. Rieder, A.C. Miller, D. Samid, Transcriptional upregulation of gamma-globin by phenylbutyrate and analogous aromatic fatty acids, Biochem Pharmacol 52 (1996) 1227–1233. https://doi.org/10.1016/0006-2952(96)00476-5.
[476] C.L. Harteveld, J.G. Heister, P.C. Giordano, D. Batelaan, P. von Delft, H.L. Haak, P.W. Wijermans, M. Losekoot, L.F. Bernini, An IVS1-116 (A-->G) acceptor splice site mutation in the alpha 2 globin gene causing alpha + thalassaemia in two Dutch families, Br J Haematol 95 (1996) 461–466. https://doi.org/10.1046/j.1365-2141.1996.d01-1926.x.
[477] D. Donze, P.H. Jeancake, T.M. Townes, Activation of delta-globin gene expression by erythroid Krupple-like factor: a potential approach for gene therapy of sickle cell disease, Blood 88 (1996) 4051–4057.
[478] K.J. Takekoshi, Y.H. Oh, K.W. Westerman, I.M. London, P. Leboulch, Retroviral transfer of a human beta-globin/delta-globin hybrid gene linked to beta locus control region hypersensitive site 2 aimed at the gene therapy of sickle cell disease, Proc Natl Acad Sci U S A 92 (1995) 3014–3018. https://doi.org/10.1073/pnas.92.7.3014.
[479] M.C. Rosatelli, V. Faà, A. Meloni, F. Fiorenza, R. Galanello, D. Gasperini, G. Amendola, A. Cao, A promoter mutation, C-->T at position -92, leading to silent beta-thalassaemia, Br J Haematol 90 (1995) 483–485. https://doi.org/10.1111/j.1365-2141.1995.tb05182.x.
[480] C. Camaschella, U. Mazza, A. Roetto, E. Gottardi, A. Parziale, M. Travi, S. Fattore, D. Bacchiega, G. Fiorelli, M.D. Cappellini, Genetic interactions in thalassemia intermedia: analysis of beta-mutations, alpha-genotype, gamma-promoters, and beta-

LCR hypersensitive sites 2 and 4 in Italian patients, Am J Hematol 48 (1995) 82–87. https://doi.org/10.1002/ajh.2830480203.
[481] S.P. Perrine, N.F. Olivieri, D.V. Faller, E.P. Vichinsky, G.J. Dover, G.D. Ginder, Butyrate derivatives. New agents for stimulating fetal globin production in the beta-globin disorders, Am J Pediatr Hematol Oncol 16 (1994) 67–71.
[482] S.P. Perrine, G.H. Dover, P. Daftari, C.T. Walsh, Y. Jin, A. Mays, D.V. Faller, Isobutyramide, an orally bioavailable butyrate analogue, stimulates fetal globin gene expression in vitro and in vivo, Br J Haematol 88 (1994) 555–561. https://doi.org/10.1111/j.1365-2141.1994.tb05073.x.
[483] J.L. Miller, R.E. Donahue, S.E. Sellers, R.J. Samulski, N.S. Young, A.W. Nienhuis, Recombinant adeno-associated virus (rAAV)-mediated expression of a human gamma-globin gene in human progenitor-derived erythroid cells, Proc Natl Acad Sci U S A 91 (1994) 10183–10187. https://doi.org/10.1073/pnas.91.21.10183.
[484] J.H. Kim, R.V. Lebo, S.P. Cai, X. Su, J.H. Chung, W.C. Mentzer, M.S. Golbus, Prenatal diagnosis of unusual hemoglobinopathies, Am J Med Genet 50 (1994) 15–20. https://doi.org/10.1002/ajmg.1320500104.
[485] J.G. Gilman, L. Manca, L. Frogheri, P. Pistidda, L. Guiso, M. Longinotti, B. Masala, Mild beta+(-87)-thalassemia CACCC box mutation is associated with elevated fetal hemoglobin expression in cis, Am J Hematol 45 (1994) 265–267. https://doi.org/10.1002/ajh.2830450316.
[486] D.G. Efremov, A.J. Dimovski, E. Baysal, Z. Ye, A.D. Adekile, M.L. Ribeiro, G. Schiliro, C. Altay, A. Gürgey, G.D. Efremov, Possible factors influencing the haemoglobin and fetal haemoglobin levels in patients with beta-thalassaemia due to a homozygosity for the IVS-I-6 (T-->C) mutation, Br J Haematol 86 (1994) 824–830. https://doi.org/10.1111/j.1365-2141.1994.tb04837.x.
[487] A.D. Adekile, L.H. Gu, E. Baysal, M.Z. Haider, L. al-Fuzae, K.C. Aboobacker, A. al-Rashied, T.H. Huisman, Molecular characterization of alpha-thalassemia determinants, beta-thalassemia alleles, and beta S haplotypes among Kuwaiti Arabs, Acta Haematol 92 (1994) 176–181. https://doi.org/10.1159/000204216.
[488] S.P. Perrine, G.D. Ginder, D.V. Faller, G.H. Dover, T. Ikuta, H.E. Witkowska, S.P. Cai, E.P. Vichinsky, N.F. Olivieri, A short-term trial of butyrate to stimulate fetal-globin-gene expression in the beta-globin disorders, N Engl J Med 328 (1993) 81–86. https://doi.org/10.1056/NEJM199301143280202.
[489] P.I. Motum, A. Kearney, T.J. Hamilton, R.J. Trent, Filipino beta zero thalassaemia: a high Hb A2 beta zero thalassaemia resulting from a large deletion of the 5' beta globin gene region, J Med Genet 30 (1993) 240–244. https://doi.org/10.1136/jmg.30.3.240.
[490] V.E. Laubach, W.J. Ryan, M. Brantly, Characterization of a human alpha 1-antitrypsin null allele involving aberrant mRNA splicing, Hum Mol Genet 2 (1993) 1001–1005. https://doi.org/10.1093/hmg/2.7.1001.
[491] J.G. Gilman, O. Josifovska, S. Erlingsson, P.F. Milner, R.L. Nagel, Direct demonstration that the A gamma T globin gene is linked to the 4 bp promoter deletion in the beta A chromosome of sickle cell traits, Am J Hematol 43 (1993) 312–315. https://doi.org/10.1002/ajh.2830430418.
[492] V. Divoky, E. Baysal, G. Schiliro, S.P. Dibenedetto, T.H. Huisman, A mild type of Hb S-beta(+)-thalassemia [-92(C-->T)] in a Sicilian family, Am J Hematol 42 (1993) 225–226. https://doi.org/10.1002/ajh.2830420216.
[493] K. Sugihara, T. Sugihara, N. Mohandas, R.P. Hebbel, Thrombospondin mediates adherence of CD36+ sickle reticulocytes to endothelial cells, Blood 80 (1992) 2634–2642.

[494] C. Oner, A.J. Dimovski, N.F. Olivieri, G. Schiliro, J.F. Codrington, S. Fattoum, A.D. Adekile, R. Oner, G.T. Yüregir, C. Altay, Beta S haplotypes in various world populations, Hum Genet 89 (1992) 99–104. https://doi.org/10.1007/BF00207052.
[495] P.I. Motum, R. Lindeman, T.J. Hamilton, R.J. Trent, Australian beta zero-thalassaemia: a high haemoglobin A2 beta zero-thalassaemia due to a 12 kb deletion commencing 5' to the beta-globin gene, Br J Haematol 82 (1992) 107–113. https://doi.org/10.1111/j.1365-2141.1992.tb04601.x.
[496] A. Meloni, M.C. Rosatelli, V. Faà, R. Sardu, L. Saba, S. Murru, G.V. Sciarratta, M. Baldi, N. Tannoia, A. Vitucci, Promoter mutations producing mild beta-thalassaemia in the Italian population, Br J Haematol 80 (1992) 222–226. https://doi.org/10.1111/j.1365-2141.1992.tb08904.x.
[497] M. Matsuda, N. Sakamoto, Y. Fukumaki, Delta-thalassemia caused by disruption of the site for an erythroid-specific transcription factor, GATA-1, in the delta-globin gene promoter, Blood 80 (1992) 1347–1351.
[498] M.B. Coleman, J.G. Adams, M.W. Plonczynski, A.H. Harrell, A.M. Walker, V. Fairbanks, M.H. Steinberg, Beta-thalassemia intermedia with exceptionally high hemoglobin A2: relationship to mutations in the beta-gene promoter, Am J Med Sci 304 (1992) 73–78. https://doi.org/10.1097/00000441-199208000-00001.
[499] A.D. Adekile, M.N. Kitundu, L.H. Gu, K.D. Lanclos, O.O. Adeodu, T.H. Huisman, Haplotypes in SS patients from Nigeria; characterization of one atypical beta S haplotype no. 19 (Benin) associated with elevated HB F and high G gamma levels, Ann Hematol 65 (1992) 41–45. https://doi.org/10.1007/BF01715125.
[500] S. Murru, G. Loudianos, M. Deiana, C. Camaschella, G.V. Sciarratta, S. Agosti, M.I. Parodi, P. Cerruti, A. Cao, M. Pirastu, Molecular characterization of beta-thalassemia intermedia in patients of Italian descent and identification of three novel beta-thalassemia mutations, Blood 77 (1991) 1342–1347.
[501] N. Mishima, E.C. Brinson, P.F. Milner, J.G. Gilman, G gamma and A gamma globin genes are identical from -471 of the promoter midway through gamma IVSII in a Benin beta s haplotype associated with elevated fetal hemoglobin, Am J Hum Genet 48 (1991) 1175–1180.
[502] G.B. Leoni, C. Rosatelli, A. Vitucci, M. Addis, A. Loi, N. Tannoia, A. Cao, Molecular basis of beta-thalassemia intermedia in a southern Italian region (Puglia), Acta Haematol 86 (1991) 174–178. https://doi.org/10.1159/000204829.
[503] A.E. Kulozik, A. Bellan-Koch, S. Bail, E. Kohne, E. Kleihauer, Thalassemia intermedia: moderate reduction of beta globin gene transcriptional activity by a novel mutation of the proximal CACCC promoter element, Blood 77 (1991) 2054–2058.
[504] Y. Fukumaki, [Molecular analysis of thalassemia], Rinsho Ketsueki 32 (1991) 587–591.
[505] S. Fattoum, F. Guemira, C. Oner, R. Oner, H.W. Li, F. Kutlar, T.H. Huisman, Beta-thalassemia, HB S-beta-thalassemia and sickle cell anemia among Tunisians, Hemoglobin 15 (1991) 11–21. https://doi.org/10.3109/03630269109072481.
[506] E.P. Economou, S.E. Antonarakis, H.H. Kazazian, G.R. Serjeant, G.J. Dover, Variation in hemoglobin F production among normal and sickle cell adults is not related to nucleotide substitutions in the gamma promoter regions, Blood 77 (1991) 174–177.
[507] P.E. Berg, M. Mittelman, J. Elion, D. Labie, A.N. Schechter, Increased protein binding to a -530 mutation of the human beta-globin gene associated with decreased beta-globin synthesis, Am J Hematol 36 (1991) 42–47. https://doi.org/10.1002/ajh.2830360109.

[508] M.S. Ristaldi, S. Murru, G. Loudianos, L. Casula, S. Porcu, D. Pigheddu, B. Fanni, G.V. Sciarratta, S. Agosti, M.I. Parodi, The C-T substitution in the distal CACCC box of the beta-globin gene promoter is a common cause of silent beta thalassaemia in the Italian population, Br J Haematol 74 (1990) 480–486. https://doi.org/10.1111/j.1365-2141.1990.tb06338.x.
[509] M. Pirastu, M.S. Ristaldi, G. Loudianos, S. Murru, G.V. Sciarratta, M.I. Parodi, D. Leone, S. Agosti, A. Cao, Molecular analysis of atypical beta-thalassemia heterozygotes, Ann N Y Acad Sci 612 (1990) 90–97. https://doi.org/10.1111/j.1749-6632.1990.tb24294.x.
[510] S.P. Perrine, D.V. Faller, P. Swerdlow, B.A. Miller, A. Bank, A.J. Sytkowski, J. Reczek, A.M. Rudolph, Y.W. Kan, Stopping the biologic clock for globin gene switching, Ann N Y Acad Sci 612 (1990) 134–140. https://doi.org/10.1111/j.1749-6632.1990.tb24299.x.
[511] S. Ottolenghi, S. Nicolis, C. Bertini, A. Ronchi, S. Crotta, B. Giglioni, Regulation of gamma-globin expression in hereditary persistence of fetal hemoglobin, Ann N Y Acad Sci 612 (1990) 191–195. https://doi.org/10.1111/j.1749-6632.1990.tb24306.x.
[512] J.F. Codrington, H.W. Li, F. Kutlar, L.H. Gu, M. Ramachandran, T.H. Huisman, Observations on the levels of Hb A2 in patients with different beta-thalassemia mutations and a delta chain variant, Blood 76 (1990) 1246–1249.
[513] S.C. Wong, T.A. Stoming, G.D. Efremov, T.H. Huisman, High frequencies of a rearrangement (+ATA; -T) at -530 to the beta-globin gene in different populations indicate the absence of a correlation with a silent beta-thalassemia determinant, Hemoglobin 13 (1989) 1–5. https://doi.org/10.3109/03630268908998048.
[514] S. Safaya, R.F. Rieder, C.E. Dowling, H.H. Kazazian, J.G. Adams, Homozygous beta-thalassemia without anemia, Blood 73 (1989) 324–328.
[515] M.C. Rosatelli, L. Oggiano, G. Battista Leoni, T. Tuveri, A. Di Tucci, M.T. Scalas, F. Dore, P. Pistidda, A. Massa, M. Longinotti, Thalassemia intermedia resulting from a mild beta-thalassemia mutation, Blood 73 (1989) 601–605.
[516] N. Mishima, J.G. Gilman, L.O. Huey, P.F. Milner, A gamma globin gene with G gamma-like promoter in a benin sickle cell anemia haplotype associated with elevated fetal hemoglobin, Prog Clin Biol Res 316B (1989) 403–408.
[517] I.S. Han, H.J. Huang, Y.T. Zeng, K.D. Lanclos, T.H. Huisman, Identical nucleotide sequences of the 3’A gamma globin gene enhancer elements from four different chromosomes, Blood 73 (1989) 845–848.
[518] E.E. Bouhassira, H. Lachman, R. Krishnamoorthy, D. Labie, R.L. Nagel, A gene conversion located 5’ to the A gamma gene in linkage disequilibrium with the Bantu haplotype in sickle cell anemia, J Clin Invest 83 (1989) 2070–2073. https://doi.org/10.1172/JCI114118.
[519] E.E. Bouhassira, R. Krishnamoorthy, A. Ragusa, C. Driscoll, D. Labie, R.L. Nagel, The enhancer-like sequence 3’ to the A gamma gene is polymorphic in human populations, Blood 73 (1989) 1050–1053.
[520] F. Baklouti, R. Ouazana, C. Gonnet, A. Lapillonne, J. Delaunay, J. Godet, Beta+-thalassemia in cis of a sickle cell gene: occurrence of a promoter mutation on a beta s chromosome, Blood 74 (1989) 1817–1822.
[521] J.G. Gilman, N. Mishima, X.J. Wen, F. Kutlar, T.H. Huisman, Upstream promoter mutation associated with a modest elevation of fetal hemoglobin expression in human adults, Blood 72 (1988) 78–81.
[522] J.G. Gilman, N. Mishima, X.J. Wen, T.A. Stoming, J. Lobel, T.H. Huisman, Distal CCAAT box deletion in the A gamma globin gene of two black adolescents with

elevated fetal A gamma globin, Nucleic Acids Res 16 (1988) 10635–10642. https://doi.org/10.1093/nar/16.22.10635.
[523] J.G. Gilman, Expression of G gamma and A gamma globin genes in human adults, Hemoglobin 12 (1988) 707–716. https://doi.org/10.3109/03630268808991664.
[524] B.A. Miller, N. Olivieri, M. Salameh, M. Ahmed, G. Antognetti, T.H. Huisman, D.G. Nathan, S.H. Orkin, Molecular analysis of the high-hemoglobin-F phenotype in Saudi Arabian sickle cell anemia, N Engl J Med 316 (1987) 244–250. https://doi.org/10.1056/NEJM198701293160504.
[525] C. Doerig, P. Beard, B. Hirt, A transcriptional promoter of the human parvovirus B19 active in vitro and in vivo, Virology 157 (1987) 539–542. https://doi.org/10.1016/0042-6822(87)90297-2.
[526] R.O. Shade, M.C. Blundell, S.F. Cotmore, P. Tattersall, C.R. Astell, Nucleotide sequence and genome organization of human parvovirus B19 isolated from the serum of a child during aplastic crisis, J Virol 58 (1986) 921–936. https://doi.org/10.1128/JVI.58.3.921-936.1986.
[527] R.K. Humphries, G. Dover, N.S. Young, J.G. Moore, S. Charache, T. Ley, A.W. Nienhuis, 5-Azacytidine acts directly on both erythroid precursors and progenitors to increase production of fetal hemoglobin, J Clin Invest 75 (1985) 547–557. https://doi.org/10.1172/JCI111731.

Supplementary Figure S1: PRISMA 2020 flow diagram for studies on non-coding genomic variants in thalassemia and SCD.

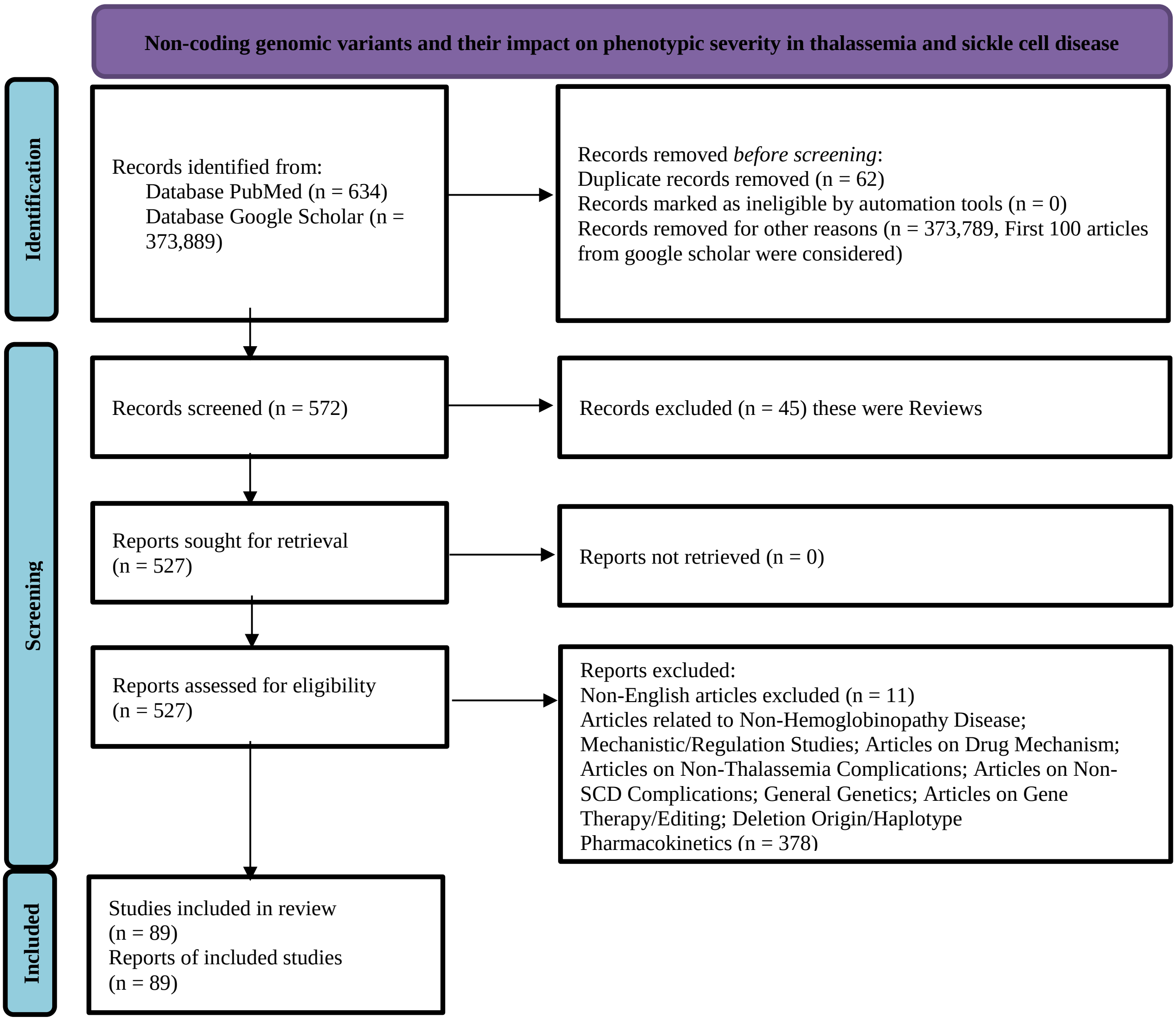

Supplementary Data S2: PRISMA 2020 Checklist and PRISMA 2020 for Abstracts Checklist

PRISMA

PRISMA 2020 Checklist

Section and Topic	Item #	Checklist item	Location where item is reported
**TITLE**			
Title	1	Identify the report as a systematic review.	Yes
**ABSTRACT**			
Abstract	2	See the PRISMA 2020 for Abstracts checklist.	Yes
**INTRODUCTION**			
Rationale	3	Describe the rationale for the review in the context of existing knowledge.	Yes
Objectives	4	Provide an explicit statement of the objective(s) or question(s) the review addresses.	Yes
**METHODS**			
Eligibility criteria	5	Specify the inclusion and exclusion criteria for the review and how studies were grouped for the syntheses.	Yes
Information sources	6	Specify all databases, registers, websites, organisations, reference lists and other sources searched or consulted to identify studies. Specify the date when each source was last searched or consulted.	Yes
Search strategy	7	Present the full search strategies for all databases, registers and websites, including any filters and limits used.	Yes
Selection process	8	Specify the methods used to decide whether a study met the inclusion criteria of the review, including how many reviewers screened each record and each report retrieved, whether they worked independently, and if applicable, details of automation tools used in the process.	Yes
Data collection process	9	Specify the methods used to collect data from reports, including how many reviewers collected data from each report, whether they worked independently, any processes for obtaining or confirming data from study investigators, and if applicable, details of automation tools used in the process.	Confirmed by both investigators. No automation tools used
Data items	10a	List and define all outcomes for which data were sought. Specify whether all results that were compatible with each outcome domain in each study were sought (e.g. for all measures, time points, analyses), and if not, the methods used to decide which results to collect.	Yes
	10b	List and define all other variables for which data were sought (e.g. participant and intervention characteristics, funding sources). Describe any assumptions made about any missing or unclear information.	Yes
Study risk of bias assessment	11	Specify the methods used to assess risk of bias in the included studies, including details of the tool(s) used, how many reviewers assessed each study and whether they worked independently, and if applicable, details of automation tools used in the process.	Limitation added
Effect measures	12	Specify for each outcome the effect measure(s) (e.g. risk ratio, mean difference) used in the synthesis or presentation of results.	Limitation added
Synthesis methods	13a	Describe the processes used to decide which studies were eligible for each synthesis (e.g. tabulating the study intervention characteristics and comparing against the planned groups for each synthesis (item #5)).	Yes
	13b	Describe any methods required to prepare the data for presentation or synthesis, such as handling of missing summary statistics, or data conversions.	Limitation added
	13c	Describe any methods used to tabulate or visually display results of individual studies and syntheses.	Yes<br>Included in Table S1
	13d	Describe any methods used to synthesize results and provide a rationale for the choice(s). If meta-analysis was performed, describe the model(s), method(s) to identify the presence and extent of statistical heterogeneity, and software package(s) used.	Limitation added
	13e	Describe any methods used to explore possible causes of heterogeneity among study results (e.g. subgroup analysis, meta-regression).	Limitation

PRISMA 2020 Checklist

Section and Topic	Item #	Checklist item	Location where item is reported
			added
	13f	Describe any sensitivity analyses conducted to assess robustness of the synthesized results.	Not applicable
Reporting bias assessment	14	Describe any methods used to assess risk of bias due to missing results in a synthesis (arising from reporting biases).	Limitation added
Certainty assessment	15	Describe any methods used to assess certainty (or confidence) in the body of evidence for an outcome.	Yes<br>Included in Table S1
**RESULTS**			
Study selection	16a	Describe the results of the search and selection process, from the number of records identified in the search to the number of studies included in the review, ideally using a flow diagram.	Yes
	16b	Cite studies that might appear to meet the inclusion criteria, but which were excluded, and explain why they were excluded.	Yes
Study characteristics	17	Cite each included study and present its characteristics.	Yes
Risk of bias in studies	18	Present assessments of risk of bias for each included study.	Limitation added
Results of individual studies	19	For all outcomes, present, for each study: (a) summary statistics for each group (where appropriate) and (b) an effect estimate and its precision (e.g. confidence/credible interval), ideally using structured tables or plots.	Limitation added
Results of syntheses	20a	For each synthesis, briefly summarise the characteristics and risk of bias among contributing studies.	Limitation added
	20b	Present results of all statistical syntheses conducted. If meta-analysis was done, present for each the summary estimate and its precision (e.g. confidence/credible interval) and measures of statistical heterogeneity. If comparing groups, describe the direction of the effect.	Limitation added
	20c	Present results of all investigations of possible causes of heterogeneity among study results.	Yes
	20d	Present results of all sensitivity analyses conducted to assess the robustness of the synthesized results.	Yes
Reporting biases	21	Present assessments of risk of bias due to missing results (arising from reporting biases) for each synthesis assessed.	Limitation added
Certainty of evidence	22	Present assessments of certainty (or confidence) in the body of evidence for each outcome assessed.	Limitation added
**DISCUSSION**			
Discussion	23a	Provide a general interpretation of the results in the context of other evidence.	Yes
	23b	Discuss any limitations of the evidence included in the review.	Yes
	23c	Discuss any limitations of the review processes used.	Yes
	23d	Discuss implications of the results for practice, policy, and future research.	Yes
**OTHER INFORMATION**			
Registration and protocol	24a	Provide registration information for the review, including register name and registration number, or state that the review was not registered.	Yes
	24b	Indicate where the review protocol can be accessed, or state that a protocol was not prepared.	Yes

PRISMA

PRISMA 2020 Checklist

Section and Topic	Item #	Checklist item	Location where item is reported
	24c	Describe and explain any amendments to information provided at registration or in the protocol.	Yes
Support	25	Describe sources of financial or non-financial support for the review, and the role of the funders or sponsors in the review.	Yes
Competing interests	26	Declare any competing interests of review authors.	Yes
Availability of data, code and other materials	27	Report which of the following are publicly available and where they can be found: template data collection forms; data extracted from included studies; data used for all analyses; analytic code; any other materials used in the review.	Yes

From: Page MJ, McKenzie JE, Bossuyt PM, Boutron I, Hoffmann TC, Mulrow CD, et al. The PRISMA 2020 statement: an updated guideline for reporting systematic reviews. BMJ 2021;372:n71. doi: 10.1136/bmj.n71. This work is licensed under CC BY 4.0. To view a copy of this license, visit https://creativecommons.org/licenses/by/4.0/

PRISMA 2020 for Abstracts Checklist

Section and Topic	Item #	Checklist item	Reported (Yes/No)
**TITLE**			
Title	1	Identify the report as a systematic review.	Yes
**BACKGROUND**			
Objectives	2	Provide an explicit statement of the main objective(s) or question(s) the review addresses.	Yes
**METHODS**			
Eligibility criteria	3	Specify the inclusion and exclusion criteria for the review.	Yes
Information sources	4	Specify the information sources (e.g. databases, registers) used to identify studies and the date when each was last searched.	Yes
Risk of bias	5	Specify the methods used to assess risk of bias in the included studies.	Limitation added
Synthesis of results	6	Specify the methods used to present and synthesise results.	Yes
**RESULTS**			
Included studies	7	Give the total number of included studies and participants and summarise relevant characteristics of studies.	Yes
Synthesis of results	8	Present results for main outcomes, preferably indicating the number of included studies and participants for each. If meta-analysis was done, report the summary estimate and confidence/credible interval. If comparing groups, indicate the direction of the effect (i.e. which group is favoured).	Yes
**DISCUSSION**			
Limitations of evidence	9	Provide a brief summary of the limitations of the evidence included in the review (e.g. study risk of bias, inconsistency and imprecision).	Yes
Interpretation	10	Provide a general interpretation of the results and important implications.	Yes
**OTHER**			
Funding	11	Specify the primary source of funding for the review.	Yes
Registration	12	Provide the register name and registration number.	Yes

From: Page MJ, McKenzie JE, Bossuyt PM, Boutron I, Hoffmann TC, Mulrow CD, et al. The PRISMA 2020 statement: an updated guideline for reporting systematic reviews. BMJ 2021;372:n71. doi: 10.1136/bmj.n71. This work is licensed under CC BY 4.0. To view a copy of this license, visit https://creativecommons.org/licenses/by/4.0/
